# Association of Country-wide Coronavirus Mortality with Demographics, Testing, Lockdowns, and Public Wearing of Masks

**DOI:** 10.4269/ajtmh.20-1015

**Published:** 2020-10-26

**Authors:** Christopher T. Leffler, Edsel Ing, Joseph D. Lykins, Matthew C. Hogan, Craig A. McKeown, Andrzej Grzybowski

**Affiliations:** 1 Department of Ophthalmology, Virginia Commonwealth University, Richmond, Virginia;; 2 Department of Ophthalmology, Hunter Holmes McGuire VA Medical Center, Richmond, Virginia;; 3 Department of Ophthalmology and Vision Sciences, University of Toronto, Toronto, Ontario, Canada;; 4 Department of Internal Medicine, Virginia Commonwealth University, Richmond, Virginia;; 5 Department of Emergency Medicine, Virginia Commonwealth University, Richmond, Virginia;; 6 School of Medicine, Virginia Commonwealth University, Richmond, Virginia;; 7 Bascom Palmer Eye Institute, Miller School of Medicine, University of Miami, Miami, Florida;; 8 Department of Ophthalmology, University of Warmia and Mazury, Olsztyn, Poland;; 9 Institute for Research in Ophthalmology, Poznan, Poland

## Abstract

We studied sources of variation between countries in per-capita mortality from COVID-19 (caused by the SARS-CoV-2 virus). Potential predictors of per-capita coronavirus-related mortality in 200 countries by May 9, 2020 were examined, including age, gender, obesity prevalence, temperature, urbanization, smoking, duration of the outbreak, lockdowns, viral testing, contact-tracing policies, and public mask-wearing norms and policies. Multivariable linear regression analysis was performed. In univariate analysis, the prevalence of smoking, per-capita gross domestic product, urbanization, and colder average country temperature were positively associated with coronavirus-related mortality. In a multivariable analysis of 196 countries, the duration of the outbreak in the country, and the proportion of the population aged 60 years or older were positively associated with per-capita mortality, whereas duration of mask-wearing by the public was negatively associated with mortality (all *P* < 0.001). Obesity and less stringent international travel restrictions were independently associated with mortality in a model which controlled for testing policy. Viral testing policies and levels were not associated with mortality. Internal lockdown was associated with a nonsignificant 2.4% reduction in mortality each week (*P* = 0.83). The association of contact-tracing policy with mortality was not statistically significant (*P* = 0.06). In countries with cultural norms or government policies supporting public mask-wearing, per-capita coronavirus mortality increased on average by just 16.2% each week, as compared with 61.9% each week in remaining countries. Societal norms and government policies supporting the wearing of masks by the public, as well as international travel controls, are independently associated with lower per-capita mortality from COVID-19.

## INTRODUCTION

The COVID-19 global pandemic caused by infection with SARS-CoV-2 has presented a major public health challenge. For reasons that are not completely understood, the per-capita mortality from COVID-19 varies by several orders of magnitude between countries.^1^ Numerous sources of heterogeneity have been hypothesized. Higher mortality has been observed in older populations and in men.^2,3^ Patient-level behaviors, such as smoking, might also have an impact.^3^ Other potentially relevant factors include economic activity, and environmental variation, such as temperature.^4^ More urban settings and increased population density would be expected to enhance viral transmission.^5^

In addition, public health responses to the COVID-19 pandemic may influence per-capita mortality. Various strategies have been implemented, ranging from robust testing programs to lockdown or stay-at-home orders, to mandates regarding social distancing and face mask usage. Practices with theoretical benefit, such as social distancing, stay-at-home orders, and implementation of mandates regarding use of masks in public spaces, must be assessed quickly, as implementation has the potential to reduce morbidity and mortality.

Mask usage by the public is postulated to decrease infection by blocking the spread of respiratory droplets,^1^ and was successfully implemented during other coronavirus outbreaks (i.e., SARS and Middle East Respiratory Syndrome).^6^ In the context of the ongoing pandemic, we assessed the impact of masks on per-capita COVID-19–related mortality, controlling for the aforementioned factors. We hypothesized that in countries where mask use was either an accepted cultural norm or favored by government policies on a national level, the per-capita mortality might be reduced, as compared with countries which did not advocate masks.

## METHODS

### Data acquisition.

To be included in the study, countries had to 1) have coronavirus mortality data listed in the publicly available Worldometer database on May 9, 2020^7^; 2) have dates of first case and first death reported by the European CDC (which did tabulate worldwide data)^8^; and 3) have an assessment of viral testing through May 9, 2020 by either 3a) report on Worldometer of numbers of coronavirus PCR tests performed^7^ or 3b) testing and lockdown policies graded by the University of Oxford coronavirus government response tracker.^9,10^

Oxford University defined and scored several composite government response indices. The stringency index was defined in terms of containment policy and public information.^9^ The government response index incorporated containment, economic measures, public information, and testing and tracing policies.^9^ The containment and health index was defined in terms of containment measures, public information, and testing and tracing policies.^9^

Archived viral testing data tabulated by Worldometer for April 2020 were also downloaded from the Internet Archive.^11^ Mean temperature in each country for each day of its outbreak, from the start of that country’s outbreak through April 16, 2020, was estimated using the average monthly temperature in the country’s largest city from public sources.^12,13^

Online news reports and government statements, including those cited by a previous review^14^ and a public database,^15^ were searched to identify countries in which the public wore masks early in the outbreak based on tradition,^16–18^ as well as countries in which the national government mandated or recommended mask-wearing by the public before April 16, 2020.

For each country, the population,^19^ fraction of the population aged 60 years and older^20^ and those aged 14 years and younger,^20^ male: female ratio per country,^20^ surface area,^19,20^ gross domestic product per capita,^21^ percent urbanization,^19,22^ prevalence of current smoking among adults,^23–26^ and prevalence of adult obesity^27–46^ were tabulated. Obesity was defined as a body mass index of 30 kg/m^2^.^27–46^ Urbanization is the fraction of the population living in an urban area.^47^ Whether a nation was an isolated political entity on an island was also recorded.

### Statistical analysis.

The prevalence of an infectious process undergoing exponential growth (or decay) appears linear over time when graphed on a logarithmic scale.^1^ Therefore, we postulated that the logarithm of the country-wide infection prevalence would be linearly related with the duration of the outbreak in each country, as defined in the following paragraph. In addition, our analysis postulated that deaths from coronavirus would follow infections with some delay.

On average, the time from infection with the coronavirus to onset of symptoms is 5.1 days,^48^ and the time from symptom onset to death is on average 17.8 days.^49^ Therefore, the time from infection to death is expected to be 23 days.^1,50^ These incubation and mortality times were prespecified.^1,50^ Therefore, the date of each country’s initial infection was estimated as the earlier of 5 days before the first reported infection, or 23 days before the first death.^8,11,51^ Deaths by May 9, 2020 would typically reflect infections beginning 23 days previously (by April 16). Therefore, we recorded the time from the first infection in a country until April 16. To summarize, the duration of the outbreak in the country was defined as the time from the estimated date of first infection (the earlier of 5 days before the first reported case or 23 days before the first death) until April 16.

We also recorded the period of the outbreak: 1) from when public mask-wearing was recommended until April 16, 2) from the mandating of international travel restrictions or quarantine until April 16, and 3) from the start of mandated limits on internal activities (i.e., lockdowns, defined as any closure of schools or workplaces, limits on public gatherings or internal movement, or stay-at-home orders) until April 16. For countries scored by Oxford University, the Oxford data were used to determine the start of international travel restrictions and lockdowns on internal activity. In addition, we calculated the mean time-weighted score for each lockdown and testing policy as graded by the University of Oxford for the duration of the country’s outbreak, from beginning through April 16.^9^ For instance, if the school closure score was one for half the country’s outbreak and two for the other half, then the mean score was 1.5.

Per-capita mortality can be analyzed as a binary outcome (low or high), or as a continuous variable. Each approach has strengths and weaknesses. Analysis of a binary outcome is not unduly influenced by outliers. Countries with extremely low or high mortality are included in the appropriate group, but the exact mortality value does not change the results. Moreover, analysis of a binary outcome facilitates clear communication because one can describe the characteristics of low and high mortality countries. We used the median value as the threshold to separate countries with low and high per-capita mortality.

On the other hand, per-capita mortality is in fact a continuous variable, and the separation of countries less than or greater than a threshold value is somewhat arbitrary, or susceptible to chance variation. Analysis of mortality as a continuous variable uses all the information available, and can appropriately model the exponential growth of an infection. We view the binary and continuous analyses as complementary. When one sees that a univariate association is found with both types of analyses, one gains confidence that the association is not an artifact of the analytic method selected.

In univariate analysis, characteristics of countries with per-capita mortality above the median value (“high-mortality” countries) were compared with the remaining (“low-mortality”) countries by the two-sample *t*-test using groups.

Significant predictors of per-capita coronavirus mortality in the univariate analysis were analyzed by stepwise backward multivariable linear regression analysis. The dependent variable was the logarithm (base 10) of per-capita coronavirus-related mortality. Because of the importance relative to public health, the weeks the country spent in lockdown, with international travel restrictions, and using masks, and per-capita testing levels, were retained in the model. In addition, because of their biological plausibility and presumed importance, urbanization, prevalence of obesity, and average ambient temperature were retained in most of the multivariable models presented in the following text. Statistical analysis was performed with XLSTAT 2020.1 (Addinsoft, New York, NY). An alpha (*P*-value) of 0.05 was deemed to be statistically significant. The study was approved by the Virginia Commonwealth University Office of Research Subjects Protection.

## RESULTS

We studied coronavirus mortality in 200 countries, of which 183 had testing data,^7^ 169 had government policies scored by Oxford University,^9^ and 152 fell into both categories.

The 100 lower-mortality countries had 0.99 deaths per million population, in contrast with an average of 93.3 deaths per million population in the 100 higher-mortality countries (*P* < 0.001, Table 1, Supplemental Table A1). The median value was 3.6 deaths per million population. The same independent variables were found to be statistically significant on univariate analysis, regardless of whether per-capita mortality was considered a binary or continuous variable, as outlined in the following text (Table 1, Supplemental Table A3).

**Table 1:** Characteristics of countries with low and high per-capita coronavirus mortality by May 9, 2020 in 200 countries

	Mean (SD)		*P*-value
	Low mortality	High mortality	
Deaths (per million)	0.99 (1.14)	93.3 (182.7)	< 0.001
Deaths (per capita, log)	−6.47 (0.75)	−4.55 (0.64)	< 0.001
Duration outbreak (weeks)	6.51 (2.87)	7.84 (2.31)	< 0.001
Duration outbreak without masks (weeks)	4.74 (2.33)	6.69 (2.34)	< 0.001
Time without international travel restrictions (weeks).	1.44 (1.96)	2.62 (2.38)	< 0.001
Duration outbreak without internal lockdown (weeks)	1.79 (1.85)	2.83 (2.08)	< 0.001
Temperature, mean (C)	22.2 (7.6)	14.1 (9.1)	< 0.001
Urban population (%)	51.5 (22.6)	70.4 (20.0)	< 0.001
GDP per capita ($)	9,060 (16,960)	27,140 (27,500)	< 0.001
Age 14 years and younger (% of population)	32.4 (9.8)	20.2 (6.6)	< 0.001
Age 60 and older (% of population)	8.8 (5.3)	18.2 (7.9)	< 0.001
Surface area (km^2^, log)	4.97 (1.19)	4.62 (1.36)	0.06
Population (log)	6.81 (1.02)	6.61 (1.05)	0.17
Prevalence males (%)	50.1 (2.1)	50.2 (4.2)	0.95
Smoking prevalence, adult (%)	13.7 (7.9)	18.4 (7.7)	< 0.001
Obesity prevalence, adult (%)	14.6 (9.0)	24.0 (7.3)	< 0.001
Tests per cap. (log) by April 4	−3.73 (1.20)	−2.65 (0.76)	< 0.001
Tests per cap. (log) by April 16	−3.09 (0.87)	−2.31 (0.67)	< 0.001
Tests per cap. (log) by May 9	−2.76 (0.86)	−1.92 (0.62)	< 0.001

GDP = gross domestic product. Durations run from the estimated date of first infection in the country until 23 days before May 9, 2020 (i.e., April 16), or the stated event (mask recommendation or lockdown). Obesity data were available for 196 countries. Testing data were available for 135 countries by April 4, 162 countries by April 16, and 183 countries by May 9.

We found that 19 of 100 low-mortality countries were isolated on islands, compared with 28 of 100 high-mortality countries (*P* = 0.18). Country surface area and population were not associated with coronavirus mortality (Table 1).

### Population characteristics.

Countries with a higher fraction of the population older than 60 years suffered higher coronavirus mortality. Countries with low mortality had on average 8.8% of their population older than 60 years, as compared with 18.2% in the high-mortality countries (*P* < 0.001, Table 1). The proportion of the population which was male was not associated with country-wide mortality (*P* = 0.95, Table 1). Smoking prevalence was on average 13.7% in low-mortality countries and 18.4% in high-mortality countries (*P* < 0.001, Table 1). The prevalence of obesity was on average 14.6% in low-mortality countries and 24.0% in high-mortality countries (*P* < 0.001, Table 1).

### Temperature.

Colder countries were associated with higher coronavirus mortality in univariate analysis. The mean temperature was 22.2 C (SD 7.6 C) in the low-mortality countries and 14.1 C (SD 9.1 C) in the high-mortality countries (*P* < 0.001, Table 1).

### Urbanization.

Urbanization was associated with coronavirus mortality in univariate analysis. In low-mortality countries, on average, 52% of the population was urban, as compared with 70% of the population in the high-mortality countries (*P* < 0.001, Table 1).

## MASKS: EARLY ADOPTION

The WHO initially advised against widespread mask-wearing by the public, as did the U.S. CDC.^1,16^ The WHO reversed course and recommended masks in public on June 5, 2020.^52^

**Figure 1.**
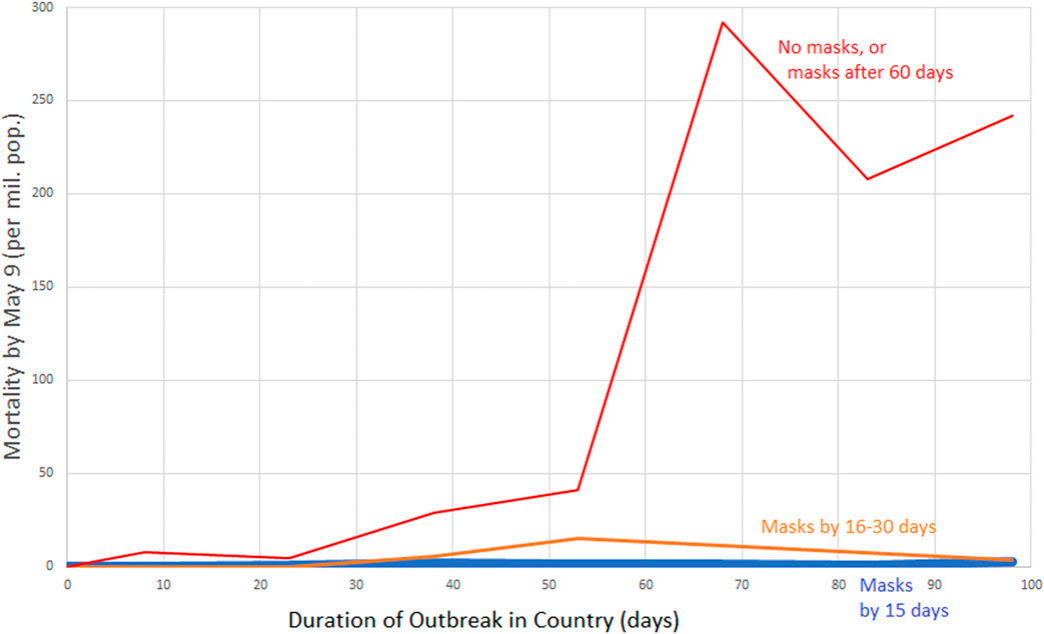
Per-capita mortality by May 9 vs. duration of the outbreak according to whether early masking was adopted. Data grouped by whether country did not recommend masks by April 16, 2020 or recommended them more than 60 days after outbreak onset (red line), recommended masks 16–30 days after onset of the country’s outbreak (orange line), or recommended masks (or traditionally used masks) within 15 days of the outbreak onset (blue line close to the *x*-axis). Country mortality was averaged for the following country groups of infection duration: 0–15 days, 16–30 days, 31–45 days, 46–60 days, 61–75 days, 76–90 days, and 91–105 days. For instance, per-capita mortality for all non-mask or late-masking countries with infection duration between 61 and 75 days was averaged, and graphed at the *x*-value 68 days. Data for graph were derived from 200 countries.

Despite these initial recommendations, a number of countries did favor mask wear by the public early in their outbreak, and such countries experienced low coronavirus-related mortality (Supplemental Tables A1 and A2, Figure 1).^17,53–70^ It is likely that in Mongolia and Laos, both of which reported no coronavirus-related mortality by May 9, the public began wearing masks before any cases were confirmed in their countries (Supplemental Table A1). We identified 24 countries with recommendations or cultural norms favoring mask-wearing by the public within 20 days of the estimated onset of the country’s outbreak,^1^ including Japan, the Philippines, Macau, Hong Kong, Sierra Leone, Cambodia, Timor-Leste, Vietnam, Malaysia, Bhutan, Venezuela, Taiwan, Slovakia, St. Kitts and Nevis, South Korea, Indonesia, Brunei, Grenada, Mozambique, Uzbekistan, Thailand, and Malawi (Supplemental Table A1). The average mortality by May 9 for these 24 early mask-wearing countries was 1.5 per million (SD 2.0). Twenty of the 24 were lower-mortality countries (*P* = 0.001).

An additional 17 countries recommended public masking within 30 days of the estimated onset of their outbreak: São Tomé and Príncipe, Czechia, Dominica, Bangladesh, Zambia, Chad, Benin, Sudan, El Salvador, Antigua and Barbuda, Myanmar, Bosnia and Herzegovina, Côte d’Ivoire, South Sudan, Kenya, Saint Lucia, and Barbados (Supplemental Table A1). The average mortality by May 9 for this group was 8.5 per million (SD 12.4).

Numerous countries recommended masks during the study exposure period, but more than 30 days after the country’s outbreak is estimated to have begun. Public mask wear was widespread in China after January 20 (Supplemental Table A2).^54,55,60,71^ The following recommended public masking in March: Kuwait,^14^ Nepal, Lithuania, the United Arab Emirates (UAE), Slovenia, Iran,^14^ Bulgaria, Ukraine, Austria, the Cayman Islands, and Mauritius (Supplemental Table A2). The following recommended public masking in April (by the 16th): Israel, Germany, Brazil, Cuba, Saint Kitts and Nevis, the United States, Singapore, Turkey, France, Cyprus, Peru, India, Colombia, El Salvador, Malawi, the Ivory Coast,^68^ Estonia, Trinidad and Tobago, Canada, Tunisia, Morocco, Honduras, the Dominican Republic, Ecuador, Paraguay, Panama, Jamaica, Poland, Guatemala, Bahrain, Guyana, Uruguay, South Africa, Spain, Ethiopia, Sri Lanka, Guinea, Nigeria,^72^ Equatorial Guinea, Finland, Luxembourg, Gabon, and Libya (Supplemental Table A2).

Throughout much of East, South, and Southeast Asia, masks were worn by the public as a preventive measure, rather than a policy implemented after evidence emerged of health system overload (Supplemental Tables A1 and A2). The public sometimes implemented masks before government recommendations were issued. For Nepal, India, and Sri Lanka, we did not score the country as mask-wearing until government recommendations were issued, but there was evidence of earlier mask wear in public (Supplemental Table A2).

In parts of the Middle East, such as Saudi Arabia and the UAE, masks were embraced by the public even before government requirements (Supplemental Table A2). As noted earlier, 11 African countries recommended or mandated masks within 31 days of the onset of their outbreak (Supplemental Table A1).

Most countries in Europe and North America failed to embrace masks early in their outbreaks and only adopted mask policies after signs of health system overload became apparent. Only three countries in Europe had government recommendations to wear masks within 31 days of the onset of their outbreak: Slovakia, Czechia, and Bosnia and Herzegovina (Supplemental Table A1).

## GRAPHICAL ANALYSIS OF MASK EFFECT

**Figure 2.**
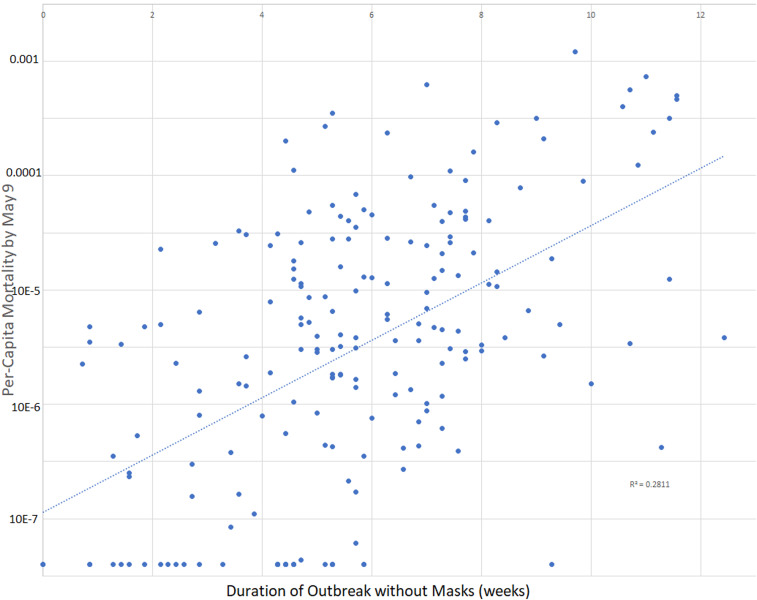
Scatterplot of per-capita mortality by May 9, 2020 as a function of the period of the country’s outbreak without mask recommendations or norms. The dotted line represents the best fit using least-squares linear regression. Data for graph were derived from 200 countries. The start of the outbreak is defined as 5 days before the first case reported, or 23 days before the first death (whichever was earlier). The duration of the outbreak without masks is defined as the time from the start of the country’s outbreak until masks were recommended or until April 16 (whichever came first).

Before the formal statistical analysis, we graphically illustrate the effect of mask wear (Figures 1 and 2). The first figure demonstrates the effect of early mask usage (Figure 1). In the countries not using masks by April 16, or not using them until 60 days after the start of the outbreak, the per-capita mortality by May 9 rises dramatically if the infection has persisted in the country over 60 days (Figure 1, red line). On the other hand, countries in which a mask was used from 16 to 30 days after infection onset had per-capita mortality several orders of magnitude less by May 9 (Figure 1, orange line). When countries recommended masks within 15 days of the onset of the outbreak, the mortality was so low that the curve is difficult to distinguish from the *x*-axis (Figure 1, blue line).

To provide some graphical idea of the scatter of the data when exponential growth is assumed, we graphed per-capita mortality by May 9 on a logarithmic scale as a function of the duration of the country’s outbreak not using masks in all 200 countries (Figure 2). This simple model explained 28.1% of the variation in per-capita mortality.

### Initial multivariable analysis.

An initial multivariable analysis was conducted including all 200 countries. By multivariable linear regression, significant predictors of the logarithm of each country’s per-capita coronavirus mortality included duration of the outbreak in the country, duration of wearing masks (*P* < 0.001), percentage of the population older than 60 years, and urbanization (all *P* ≤ 0.008, Supplemental Table A4). The association of mortality with the timing of international travel restrictions was of borderline statistical significance (*P* = 0.049). The model explained 48.1% of the variation in per-capita mortality (Supplemental Table A4).

Was the association of mask usage with lower mortality an artifact of including countries for which the outbreak had only recently arrived? We repeated the model of Supplemental Table A4 using only the 187 countries for which the outbreak was estimated to have begun at least 60 days before the mortality assessment on May 9 (i.e., by March 17). Once again, masks were significantly associated with lower mortality (*P* < 0.001). Each week that masks were recommended during the outbreak was associated with an 8.1% increase in per-capita mortality (instead of the 55.7% increase seen each week when masks were not recommended). The prevalence of age older than 60 years (*P* < 0.001) and urbanization (*P* = 0.03) were associated with higher per-capita mortality, whereas the time since the start of international travel restrictions continued to be associated with lower mortality (*P* = 0.045). Duration of the lockdown (*P* = 0.83) and temperature (*P* = 0.99) were not associated with mortality. The model explained 48.7% of the variation in per-capita mortality.

We also prepared a multivariable model to predict the logarithm of per-capita coronavirus mortality in the 196 countries with obesity data. In this model, lockdown, obesity, temperature, and urbanization were retained because of their plausibility as important factors (Table 2). By multivariable linear regression, significant predictors of the logarithm of each country’s per-capita coronavirus mortality included duration of the outbreak in the country, duration of wearing masks, and percentage of the population older than 60 years (all *P* < 0.001, Table 2). The associations of obesity and urbanization with increased mortality were not statistically significant (both *P* = 0.10, Table 2). When controlling for the duration of the outbreak in the country, there appeared to be a negative association between mortality and time in lockdown (*P* = 0.83) and time with international travel restrictions (*P* = 0.07), although neither association reached statistical significance (Table 2). The model explained 50.8% of the variation in per-capita mortality.

**Table 2:** Predictors of (log) country-wide per-capita coronavirus mortality by May 9 by multivariable linear regression in 196 countries

	10^coefficient^	Coefficient (SE)	95% CI	*P*-value
Duration in country (weeks)	1.6193	0.209 (0.036)	0.138 to 0.281	< 0.001
Time wearing masks (weeks)	0.7174	−0.144 (0.030)	−0.204 to −0.084	< 0.001
Time in internal lockdown (weeks)	0.9761	−0.0105 (0.050)	−0.109 to 0.088	0.83
Time since the start of international travel restrictions (weeks)	0.8634	−0.0638 (0.035)	−0.133 to 0.005	0.07
Population, age ≥ 60 years (%)	1.1180	0.0485 (0.010)	0.028 to 0.069	< 0.001
Urbanization (%)	1.0139	0.00599 (0.004)	−0.001 to 0.013	0.10
Obesity prevalence (%)	1.0339	0.0145 (0.009)	−0.003 to 0.032	0.10
Temperature, ambient (C)	0.9904	−0.0042 (0.009)	−0.022 to 0.013	0.64
Constant	–	−7.659 (0.396)	−8.44 to −6.88	< 0.001

The duration of outbreak in country was from estimated date of first infection until 23 days before May 9, 2020 (i.e., April 16). Mask and lockdown durations run from the stated event (mask recommendation or lockdown) or estimated date of first infection in the country (whichever was later) until 23 days before May 9, 2020 (i.e., April 16). Model *r*^2^ = 0.508.

In countries not recommending masks, the per-capita mortality tended to increase each week by a factor of 1.619, or 61.9%. By contrast, in countries recommending masks, the per-capita mortality tended to increase each week by a factor of (1.6193) (0.7174) = 1.162, or just 16.2%. With international travel restrictions in place (without masks), the per-capita mortality increased each week by (1.6193) (0.8634) = 1.398, or 39.8%. Under lockdown (without masks), the per-capita mortality increased each week by (1.6193) (0.9761) = 1.581, or 58.1%, that is, slightly less than the baseline condition (Table 2).

A country with 10% more of its population living in an urban environment than another country tended to suffer mortality 14.8% higher (10^0.0599^ = 1.148, Table 2). A country in which the percentage of the population aged 60 years or older is 10% higher than in another country tended to suffer mortality 206% higher (10^0.485^ = 3.06, Table 2). A country with a prevalence of obesity 10% higher tended to suffer mortality 40% higher (10^0.145^ = 1.40, Table 2).

## NUMBERS OF VIRAL TESTS

Among the 183 countries with viral (PCR) testing data by May 9, per-capita testing performed at all three time points was positively associated with per-capita mortality in univariate analysis (all *P* < 0.001, Table 1). By May 9, 2020, low-mortality countries had performed one test for every 575 members of the population, whereas high-mortality countries had performed one test for every 83 members of the population (*P* < 0.001, Table 1).

To the multivariable model (Table 2), we added testing by May 9, using data from 179 countries with both testing and obesity data. Duration of the outbreak in the country, the duration that masks were recommended, and age at least 60 years continued to be significant predictors of per-capita mortality (all *P* ≤ 0.001, Supplemental Table A5). The model explained 52.3% of the variation in per-capita mortality. Each week the infection persisted in a country without masks was associated with a 62.5% increase in per-capita mortality (Supplemental Table A5). By contrast, in countries where masks were recommended, the per-capita mortality tended to increase each week by 19.6% (because [1.6253] [0.7357] = 1.196, Supplemental Table A5). In this model, the prevalence of obesity was associated with increased country-wide per-capita mortality, although the association was not significant (*P* = 0.09). If the prevalence of obesity increased by 10% (e.g., from 10% to 20% of a population), the per-capita mortality tended to increase by 48% (Supplemental Table A5)

In this model, a 10-fold increase (i.e., one logarithm) in per-capita testing tended to be associated with a 25.1% increase in reported per-capita mortality, although the positive association was not statistically significant (*P* = 0.40, Supplemental Table A5).

If early testing lowers mortality, one might expect negative regression coefficients. Testing on April 16 and on May 9 was added to the multivariable model of Table 3, using data from the 158 countries with both obesity and testing data by these dates. Per-capita testing (log) by April 16 was not negatively associated with per-capita mortality (log) by May 9 (coefficient 0.264, 95% CI: −0.323 to 0.851, *P* = 0.38).

**Table 3:** Government policies in 169 countries with low and high per-capita coronavirus mortality by May 9, 2020

	Mean (SD)		*P*-value
	Low mortality	High mortalilty	
School closing (0–3)	2.08 (0.65)	1.84 (0.49)	0.006
Workplace closing (0–3)	1.21 (0.74)	1.34 (0.47)	0.19
Cancel public events (0–2)	1.39 (0.45)	1.21 (0.34)	0.005
Restrictions on gatherings (0–4)	2.00 (0.84)	1.76 (0.87)	0.07
Close public transport (0–2)	0.64 (0.51)	0.58 (0.45)	0.41
Stay-at-home requirements (0–3)	0.84 (0.61)	0.89 (0.46)	0.52
Internal movement restrictions (0–2)	0.92 (0.52)	0.85 (0.38)	0.33
International travel controls (0–4)	2.88 (0.72)	2.43 (0.83)	< 0.001
Income support (0–2)	0.15 (0.24)	0.55 (0.41)	< 0.001
Public information campaigns (0–2)	1.70 (0.36)	1.62 (0.44)	0.19
Testing policy (0–3)	1.12 (0.57)	1.05 (0.48)	0.35
Contact tracing (0–2)	1.08 (0.66)	1.02 (0.60)	0.53
Stringency index (0–100)	53.4 (14.6)	49.4 (12.9)	0.06
Government response index (0–100)	45.9 (11.7)	44.8 (10.7)	0.53
Containment and health index (0–100)	52.0 (13.1)	48.2 (11.7)	0.047
Economic support index (0–100)	11.9 (13.7)	26.0 (16.6)	< 0.001

Government policies were scored by Oxford University.^9^ Characterization as low or high mortality was defined by the median for all 200 countries.

Likewise, testing on April 4 (the earliest archived data) and on May 9 was added to the multivariable model of Table 3, using data from the 131 countries with both obesity and testing data by these dates. Per-capita testing (log) by April 4 was not significantly associated with per-capita mortality (log) by May 9 (coefficient −0.0504, 95% CI: −0.378 to 0.278, *P* = 0.76). Given the coefficient, a 10-fold (one log) increase in early testing would be associated with a (nonsignificant) decrease in per-capita mortality of 11.0%.

Only five countries had performed over one test for every 10 people in the country by May 9, 2020: the Faeroe Islands, Iceland, the Falkland Islands, the UAE, and Bahrain. The highest mortality among this group was 29.0 per million population, seen in Iceland.

## CONTAINMENT AND TESTING POLICIES

For 169 countries, containment, testing, and health policies were scored by Oxford University.^9^ The following countries with mask policies by April 16 were included in this analysis, but not in the previous multivariable model, for lack of data on numbers of tests performed: China, Macau, Cameroon, Sierra Leone, and Sudan. In univariate analysis, scores for school closing, canceling public events, international travel controls, and index of containment and health were significantly associated with lower per-capita mortality (all *P* < 0.05, Table 3). Policies regarding workplace closing, restrictions on gatherings, closing public transport, stay-at-home requirements, internal movement restrictions, public information campaigns, testing, and contact tracing were not significant predictors of mortality (all *P* > 0.05, Table 3). Likewise, overall indices of stringency and government response were not associated with mortality (both *P* > 0.05, Table 3).

A multivariable model in 169 countries found that duration of the infection, duration masks were recommended, prevalence of age at least 60 years, obesity, and international travel restrictions were independently predictive of per-capita mortality (Table 4). The model explained 66.6% of the variation in per-capita mortality. At baseline, each week of the infection in a country without masks was associated with an increase in per-capita mortality of 50.7% (Table 4). By contrast, for each week that masks were worn, the per-capita mortality was associated with a lesser increase of 12.6% each week (given that 1.5072 [0.7471] = 1.126, Table 4).

**Table 4:** Predictors of (log) country-wide per-capita coronavirus mortality by May 9 by multivariable linear regression in 169 countries

	10^coefficient^	Coefficient (SE)	95% CI	*P*-value
Duration in country (weeks)	1.5072	0.1782 (0.031)	0.117 to 0.239	< 0.001
Time wearing masks (weeks)	0.7471	−0.1266 (0.026)	−0.177 to −0.076	< 0.001
Time in lockdown (weeks)	1.0184	0.0079 (0.043)	−0.077 to 0.093	0.85
Time since the start of international travel restrictions (weeks)	0.8283	−0.0818 (0.030)	−0.140 to −0.023	0.006
Population, age ≥ 60 years (%)	1.1725	0.0691 (0.009)	0.051 to 0.087	< 0.001
Urbanization (%)	1.0151	0.0065 (0.003)	−0.0003 to 0.013	0.06
Obesity prevalence (%)	1.0461	0.0196 (0.008)	0.003 to 0.036	0.02
Temperature, ambient (C)	1.0193	0.0083 (0.008)	−0.007 to 0.023	0.28
Testing policy (0–3)	1.0298	0.0127 (0.111)	−0.207 to 0.233	0.91
Contact tracing (0–2)	0.6674	−0.176 (0.092)	−0.357 to 0.006	0.06
Constant	–	−7.885 (0.347)	−8.57 to −7.20	< 0.001

The duration of outbreak in country was from estimated date of first infection until 23 days before May 9, 2020 (i.e., April 16). Mask and lockdown durations run from the stated event (mask recommendation or lockdown) or estimated date of first infection in the country (whichever was later) until 23 days before May 9, 2020 (i.e., April 16). Policies on testing, contact tracing, and international travel controls were scored by Oxford University. Model *r*^2^ = 0.666.

International travel restrictions were scored by Oxford as (0) no measures, (1) screening, (2) quarantine arrivals from high-risk regions, and ban on arrivals from some (3) or all (4) regions. The international travel restrictions were scored as four in Greenland, 3.8 in Bermuda, 3.6 in Israel, 3.5 in Czechia and New Zealand, 3.1 in Taiwan, and 2.9 in Australia, and at the other extreme, were scored as 1.1 in Sweden, and as 0 in Iran, Luxembourg, and the United Kingdom.

International travel restrictions were associated with lower mortality, regardless of whether incorporated in the model as time since onset, or as mean score during the outbreak. We present the model based on the former because of the strength of the association, and for consistency with the models presented previously. The regression analysis suggested that for each week of travel restrictions (without masks), the per-capita mortality increased by 24.8% (given that 1.5072 [0.8283] = 1.248, Table 4).

Per-capita mortality was not significantly associated with policies regarding either testing policy (*P* = 0.91), or contact tracing (*P* = 0.06, Table 4). Testing policy was scored as no policy (0), symptomatic with exposure, travel history, hospitalization, or key occupation (1), all symptomatic (2), or open to anyone (3). Testing policy tended to be positively associated with mortality. Contact tracing was scored as none (0), some cases (1), or all cases (2), and tended to be inversely related with per-capita mortality (although not significantly). As compared with a country with no contact tracing policy, comprehensive contact tracing might be associated with a 55.5% reduction in reported per-capita mortality (given that 10^2×[−0.176]^ = 0.445). However, statistical significance for this association was not demonstrated. Thus, testing and tracing may be important factors, but seem unlikely to account for most of the 100-fold variation in per-capita mortality between low- and high-mortality countries early in the course of the pandemic.

### Survey-modified model.

Surveys of mask-wearing by the public during the exposure period were available for 42 countries (Supplemental Table A2). To determine the influence that actual mask wear, as opposed to mask policies, might have on the model, we scored countries as mask-wearing if at least 50% of the public wore a mask, and non–mask-wearing if less than 50% of the population did so.

Based on surveys, Canada, Finland, France, Germany, and Malawi were not considered mask-wearing countries at any time during the exposure period (ending April 16). By contrast, Italy was scored as mask-wearing beginning March 19, Spain and India beginning March 21, Saudi Arabia beginning April 1, Russia beginning April 4, Singapore beginning April 10, and the United States, Brazil, and Mexico beginning April 12.^54,55^

In this survey-modified model in 200 countries, duration of the outbreak, duration of mask wear, proportion of the population aged 60 years or older, and urbanization were all significant predictors of per-capita mortality (all *P* < 0.01, Supplemental Table A6). Time since the start of international travel restrictions tended to be inversely associated with mortality (*P* = 0.051). Each week that the infection persisted in the country without masks was associated with a 59.9% increase in per-capita mortality. On the other hand, when masks were worn, the per-capita mortality only increased by 9.3% weekly (1.5993) (0.6836) = 1.093 (Supplemental Table A6). The model explained 48.3% of the variance in mortality.

## DISCUSSION

These results confirm that in the first 4 months of 2020, there was marked variation between countries in mortality related to COVID-19. Countries in the lower half of mortality experienced an average COVID-19–related per-capita mortality of 0.99 deaths per million population, in contrast with an average of 93.3 deaths per million in the remaining countries. Depending on the model and dataset evaluated, statistically significant independent predictors of per-capita mortality included urbanization, fraction of the population aged 60 years or older, prevalence of obesity, and duration of the outbreak in the country. In addition, per-capita mortality was inversely (and independently) associated with international travel restrictions and the period of the outbreak subject to cultural norms or government policies favoring mask-wearing by the public.

These results support the universal wearing of masks by the public to suppress the spread of the coronavirus.^1^ Given the low levels of coronavirus mortality seen in the Asian countries which adopted widespread public mask usage early in the outbreak, it seems highly unlikely that masks are harmful.

The variation in national mask norms and recommendations at the start of the pandemic provided a unique opportunity to learn about the benefits of public mask wear. On April 30, 2020, we originally published the finding that the logarithm of per-capita coronavirus mortality is linearly and positively associated with the duration of the outbreak without mask norms or mandates.^50^ This key finding was recently confirmed using mortality data from June 24, 2020 by Goldman Sachs chief economist Hatzius.^73^ Their analysis confirmed that, for prediction of both infection prevalence and mortality, the significance of the duration of mask mandates or norms in the model persists after controlling for age of the population, obesity, population density, and testing policy.^73^ Other work has confirmed that wearing masks during the pandemic can provide substantial economic value.^74^

Although a complete analysis of the later period in the pandemic is beyond the scope of the current model, we might note the coronavirus-related mortality among early-masking countries, on August 9, 2020.^7^ Among the 24 countries which initiated public mask-wearing within 20 days of the onset of their outbreak (Supplemental Table A1), the average coronavirus-related mortality was 4.7 per million (SD 6.1) on August 9. Moreover, among the additional 17 countries which recommended masks within 30 days of the start of their outbreak (Supplemental Table A1), the average coronavirus-related mortality was 26.6 per million (SD 36.2) on August 9. By contrast, the per-capita coronavirus mortality in the United States was 502 per million on August 9.

Currently, almost all countries now recommend masks in crowded, indoor spaces (Supplemental Table A2). Therefore, countries are distinguished primarily not by the stated recommendation, but by their actual mask-wearing. Surveys and observational data of mask-wearing by the public are unavailable for most countries. Our review of the literature is one of the more complete evaluations of the question to date. Available scholarship and surveys do corroborate reports in the news media that mask-wearing was common in public in many Asian countries, including Japan, the Philippines, Hong Kong, Vietnam, Malaysia, Taiwan, Thailand, China, Indonesia, India, Myanmar, and Bangladesh (Supplemental Table A2). Internet search data are consistent with interest in masks developing much earlier in the course of the pandemic in Asia than elsewhere.^75,76^ Mask-wearing was widespread in some low-mortality countries even before, or in the absence of, a formal government recommendation.

Public mask-wearing is best assessed by direct observation, rather than by surveys. A low fraction of respondents reporting mask wear in a given week might still represent high compliance if few of them visited a crowded indoor space. Conversely, a high fraction reporting mask wear in a given week might represent poor compliance if the respondents only wore a mask during a portion of their outings, wore the mask incorrectly, or were less than forthright with their responses. Available observations do confirm high levels of mask wear in East Asia, with heterogeneity in some western regions.

In addition, it is likely that the policies favoring mask-wearing in parts of the Middle East, Africa, Latin America, and the Caribbean were markers of a general cultural acceptance of masks that helped to limit the spread of the virus. Had there been adequate survey data to fully reflect the early wearing of masks in these regions, it is possible that the association of masks with lower mortality would be even stronger.

Conversely, in Western countries which had no tradition of mask-wearing, and which only recommended (rather than mandated) mask-wearing by the public, such as the United States, the practice has been steadily increasing, but change has not been immediate.

Much of the randomized controlled data on the effect of mask-wearing on the spread of respiratory viruses relate to influenza. One recent meta-analysis of 10 trials in families, students, or religious pilgrims found that the relative risk for influenza with the use of face masks was 0.78, a 22% reduction, although the findings were not statistically significant.^77^ Combining all the trials, there were 29 cases in groups assigned to wear masks, compared with 51 cases in control groups.^77^ The direct applicability of these results to mask-wearing at the population level is uncertain. For instance, there was some heterogeneity in methods of the component trials, with one trial assigning mask-wearing to the person with a respiratory illness, another to his close contacts, and the remainder to both the ill and their contacts.^77^ Mask-wearing was inconsistent. The groups living together could not wear a mask when bathing, sleeping, eating, or brushing teeth.^78–80^ In one of the studies reviewed, parents wore a mask during the day, but not at night when sleeping next to their sick child.^80^ In a different trial, students were asked to wear a mask in their residence hall for at least 6 hours daily (rather than all the time).^78^ The bottom line is that it is nearly impossible for people to constantly maintain mask wear around the people with whom they live. By contrast, wearing a mask when on public transit or shopping is quite feasible. In addition, as an infection propagates through multiple generations in the population, the benefits multiply exponentially. Even if one accepts that masks would only reduce transmissions by 22%, then after 10 cycles of the infection, mask-wearing would reduce the level of infection in the population by 91.7%, as compared with a non–mask-wearing population, at least during the period of exponential growth (because 0.78^10^ = 0.083). It is highly unlikely that entire countries or populations will ever be randomized to either wear, or not wear, masks. Public policies can only be formulated based on the best evidence available.

Some countries which used masks were better able to maintain or resume normal business and educational activities. For instance, in Taiwan, schools reopened on February 21, 2020, with parents directed to purchase four to five masks per week for each child.

Limits on international travel were significantly associated with lower per-capita mortality from coronavirus. On the other hand, nationwide policies to ban large gatherings and to close schools or businesses tended to be associated with lower mortality, although not in a statistically significant fashion. However, businesses, schools, and individuals made decisions to limit contact, independent of any government policies. The adoption of numerous public health policies at the same time can make it difficult to tease out the relative importance of each.

Our findings are consistent with observations that obesity is associated with negative outcomes, including intensive care unit admission and mechanical ventilation, in the setting of COVID-19.^81–83^ Of course, some of the observed association of obesity with mortality may be the result of unknown confounders.

Colder average monthly temperature was not associated with higher levels of COVID-19 mortality when accounting for other independent variables. One reason that outdoor temperature might have limited association with the spread of the virus is that most viral transmission occurs indoors.^84^ We acknowledge that using the average temperature in the country’s largest city during the outbreak does not model the outbreak as precisely as modeling mortality and temperature separately in each of the thousands of cities around the world. However, to a first approximation, our method did serve to control for whether the country’s climate was tropical, temperate, or polar, and whether the outbreak began in late Winter (Northern Hemisphere) or late summer (Southern Hemisphere). Environmental factors which could influence either human behavior or the stability and spread of virus particles are worthy of further study.

Presumably, high levels of testing might identify essentially all coronavirus-related deaths, and still higher levels of testing, combined with contact tracing, might lower mortality. Statistical support for the benefit of mass testing could not be demonstrated. It seems likely that countries which test at a low level are missing many cases. We identified just five countries (Iceland, the Faeroe Islands, the UAE, the Falkland Islands, and Bahrain) which had tested more than one-tenth of their population by May 9. All five countries had a mortality of 29 per million (one in 34,480 people) or less. The degree to which these results would apply to larger, less isolated, or less wealthy countries is unknown. Statistical support for benefit of high levels of testing might be demonstrated if additional and more diverse countries are able to test at this level. The benefits of contact-tracing policies with respect to mortality were of marginal statistical significance (*P* = 0.06).

The model identified predictors of coronavirus-related mortality early in the course of the pandemic. It is conceivable that additional predictors of mortality might emerge with time. For instance, policies regarding internal lockdown and contact tracing might be associated with mortality as the pandemic progresses if these policies require a longer period to reach their full effect. Conversely, international travel restrictions might become less important over time, because if the virus becomes widespread within a country then closing the border will have a lesser impact.

One limitation of our study is that the ultimate source of mortality data is often from governments which may not have the resources to provide a full accounting of their public health crises, or an interest in doing so. Countries may vary in the accuracy of their reporting. It should be noted that the benefit of wearing masks persisted in a model which excluded data from China (Supplemental Table A5).

We acknowledge that country-wide analyses are subject to the ecologic fallacy. There is potential for confounding at the ecologic level, and information bias at both the individual and ecologic levels.

However, multiple studies of coronavirus morbidity and mortality have treated countries as the unit of analysis.^85–87^ Assessment at the population level is particularly suitable for assessment of the effect of masks. If masks offer source control by blocking the spread of respiratory droplets, then those who wear masks are protecting those who do not wear masks.

We modeled the growth of the pandemic as an exponential curve (which is linear with time on a logarithmic scale) because infectious diseases are often modeled as obeying exponential processes early in their course.^88^ We recognize that all mathematical models are merely idealizations of more complicated dynamics. Future modeling work could explore the utility of nonlinear terms (e.g., quadratic and cubic), particularly as the pandemic progresses.

Available surveys suggest that there can be a delay between recommendations and changes in actual mask wear. Similarly, there might be lag periods between changes in other policies or risks and corresponding changes in infection levels which were not accounted for by the model.

The source for mortality and testing data we selected is publicly available,^7^ has been repeatedly archived,^11^ contains links to the source government reports for each country, agrees with other coronavirus aggregator sites,^89^ and has been used in other scholarly works.^85,90–96^ We presented the per-capita mortality data in Supplemental Table A2. One might question whether any of these data sites or governments provide a complete and accurate picture of coronavirus mortality. But we must remember that this information does not exist in a vacuum. Independent sources confirm when mortality has been high. Social media alerted the world to the outbreaks in Wuhan, Iran, Italy, and New York. News reports have used aerial photography to confirm the digging of graves in Iran, New York, and Brazil. Long lines were seen to retrieve remains at crematoria in Wuhan. Mortuary facilities were inadequate to meet the demand in New York, and Guayaquil. Conversely, signs of health system overload have been noted to be absent in the countries reporting low mortality. The health systems in Hong Kong, Taiwan, Japan, and South Korea are believed to be transparent. Reporters in Vietnam have called hospitals and funeral homes to confirm the absence of unusual levels of activity. Therefore, although no source is perfect, we believe that the data we used are consistent with observations from nongovernmental sources, and comparable in reliability to that in other scholarly works.

It is not the case that countries which reported no deaths due to coronavirus simply were not exposed to the virus. All 200 countries analyzed did report COVID-19 cases. Several countries which traditionally use masks and sustained low mortality (or none) are close to and have strong travel links to China. Some of these countries reported cases early in the global pandemic (Supplemental Table A1). Community transmission has been described in Vietnam.

In summary, older age of the population, urbanization, obesity, and longer duration of the outbreak in a country were independently associated with higher country-wide per-capita coronavirus mortality. International travel restrictions were associated with lower per-capita mortality. However, other containment measures, testing and tracing polices, and the amount of viral testing were not statistically significant predictors of country-wide coronavirus mortality, after controlling for other variables. By contrast, societal norms and government policies supporting mask-wearing by the public were independently associated with lower per-capita mortality from COVID-19. The use of masks in public is an important and readily modifiable public health measure.

## Supplemental appendix tables

Supplemental materials
