# Supplementary material for: Association of Country-wide Coronavirus Mortality with Demographics, Testing, Lockdowns, and Public Wearing of Masks
Source: Am J Trop Med Hyg. 2020 Oct 26;103(6):2400–11. doi: 10.4269/ajtmh.20-1015 (PMC7695060; doi:10.4269/ajtmh.20-1015)
Supplement: Supplementary file 1 [file tpmd201015.SD1.docx]

**Supplemental Appendix.**

**Supplemental Table A1**. **Countries in which masks were widely used by the public or recommended by the government within 30 days of the estimated local onset of the outbreak, by timeliness of mask-wearing**.

|  | Mask Delay (days) | First Case Date | Mortality (per mil.), by May 9. | Comment. |
| --- | --- | --- | --- | --- |
| Mongolia | 0 | Mar. 10 | 0.0 | Wearing masks began in January,^YS218^ and was mandated on transport by mid-February.^S220^ |
| Laos | 0 | Mar. 24 | 0.0 | Health officials in Laos advised mask-wearing by Mar. 6.^S190,S191^ |
| Japan | 5 | Jan. 16 | 4.8 | Public masking was manifest by Jan. 16.^S177,S178,S179^ MWP was >60% by Mar. 14.^54^ |
| Philippines | 5 | Jan. 30 | 6.4 | Masks were used extensively by Jan. 30.^S262^ MWP was 60% by Feb. 24.^55^ |
| Macau | 6 | Jan. 22 | 0.0 | By Jan. 23, the government was distributing masks to the public.^S203^ |
| Hong Kong | 6 | Jan. 23^S147^ | 0.5 | Masks were recommended on transport on Jan. 24.^16,S146^ MWP was 73% by Jan. 21.^S148^ |
| Sierra Leone | 6 | Mar. 31^S287^ | 2.3 | Masks were recommended in public on Apr. 1.^S288^ Compliance has been incomplete.^S289^ |
| Cambodia | 6 | Jan. 27^S54^ | 0.0 | Masks were widely used in public by Jan. 28.^S55,S56^ |
| Vietnam | 9 | Jan. 23 | 0.0 | Masks were widely used by Jan. 27.^S359,S360^ and mandated on Mar. 16.^S362^ MWP was >85% by Mar. 12.^54^ |
| Malaysia | 10 | Jan. 25 | 3.3 | Masks were widely used by Jan. 30,^S208^ and 55% wore a mask in public by Feb. 24.^55^ |
| Bhutan | 10 | Mar. 6^S37^ | 0.0 | On Mar. 11, the Ministry of Health advised wearing of masks in “a crowded place”.^S38^ |
| Venezuela | 10 | Mar. 13^S355^ | 0.4 | The president demonstrated mask wear on Mar. 13, and required masks in transport.^S354,S355^ |
| Taiwan | 11 | Jan. 21 | 0.3 | By Jan. 27, the government had to control mask distribution.^S319^ By Feb. 25, over 80% of the public wore a mask when out.^55^ |
| Timor-Leste | 12 | Mar. 21^S328^ | 0.0 | Masks were required in stores and other venues on Mar. 28.^S329^ |
| Slovakia | 13 | Mar. 7 | 4.8 | Masks were mandated in shops and transit on Mar. 15.^S294^ |
| St. Kitts and Nevis | 14 | Mar. 24^S271^ | 0.0 | On Apr. 2, the Chief Medical Officer recommended wearing masks in public.^S272^ |
| South Korea | 15 | Jan. 20 | 5.0 | Children were advised to wear masks at school by Jan. 30.^S301^ Stores were selling out of masks by Feb. 3.^S302^ |
| Indonesia | 15 | Mar. 2^S156^ | 3.5 | The public scrambled to buy face masks in early February.^S155^ By Feb. 24, 54% of adults wore a mask in public.^55^ |
| Brunei | 18 | Mar. 9^S48^ | 2.3 | On Mar. 22, Sultan Hassanal Bolkiah advised the people to wear masks in public.^S49^ |
| Grenada | 18 | Mar. 21^S136^ | 0.0 | Masks were recommended on Apr. 3,^S137^ and mandated on Apr. 6.^S138^ |
| Mozambique | 18 | Mar 22^S224^ | 0.0 | Masks in public were recommended on Apr. 4,^S225^ and mandated on Apr. 8.^S226^ |
| Uzbekistan | 19 | Mar. 15^S352^ | 0.3 | Masks were mandated on Mar. 25.^S353^ |
| Thailand | 20 | Jan. 13 | 0.8 | Masks were recommended by Jan. 28,^S323-S326^ and worn by 73% by Feb. 24.^55^ |
| Malawi | 20 | Apr. 2 | 0.2 | Masks were mandated on Apr. 4,^S206^ with poor compliance in one area.^S207^ |
| São Tomé and Príncipe | 21 | Apr. 6^S278^ | 22.8 | A public mask mandate effective Apr. 24 was announced Apr. 22.^65^ |
| Czechia | 23 | Mar. 1^S85^ | 25.8 | Masks were required in public on Mar. 19.^S86^ |
| Dominica | 23 | Mar. 22^S91^ | 0.0 | Masks were recommended by the president on Apr. 9^S92^ and mandated on Apr. 25.^S95^ |
| Bangladesh | 24 | Mar. 8^S21^ | 1.3 | Surveys reported mask-wearing in 60% by Mar. 19,^S23^ and 99% during April.^S24^ |
| Zambia | 24 | Mar. 18 | 0.4 | Masks were recommended on Apr. 4,^S366^ and mandated on Apr. 16.^S368^ |
| Chad | 24 | Mar. 19^S56^ | 1.9 | On Apr. 13, the president mandated masks, effective Apr. 14.^68,S57^ |
| Benin | 26 | Mar. 16^S33^ | 0.2 | Masks were recommended in public on Apr. 6,^S34^ mandated on Apr. 7.^S35,S36^ |
| Sudan | 27 | Mar. 12 | 1.5 | Free masks were dispensed by Mar. 16^S313,S314^ and were widely adopted.^S315^ |
| El Salvador | 27 | Mar. 18^S108^ | 2.6 | The president recommended masks on Apr. 4,^S109^ and mandated them on Apr. 14.^S111^ |
| Antigua and Barbuda | 28 | Mar. 13^S11^ | 30.6 | Masks were required in all public spaces on Apr. 5.^S12^ |
| Myanmar | 28 | Mar. 23^S227^ | 0.1 | From Mar. 3-20, 72% reported willingness to wear masks in crowds.^S233^ Masks were recommended on Apr. 5.^S230^ |
| Bosnia and Herzegovina | 29 | Mar. 5^S41^ | 31.1 | Masks were required in public by Mar. 29.^S42,S43^ |
| Côte d'Ivoire | 29 | Mar. 11^S173^ | 0.8 | On Apr. 4, senior health officials recommended masks when in public.^68,S174^ |
| South Sudan | 29 | Apr. 5^S304^ | 0.0 | Masks were recommended in public on Apr. 29.^S305^ |
| Kenya | 30 | Mar. 12^S184^ | 0.6 | Masks were mandated on public transport on Apr. 2.^S186,S187^ By May 5, 89% had worn a face mask in the previous week in Nairobi.^S188^ |
| Saint Lucia | 30 | Mar. 13^S274^ | 0.0 | On Apr. 7, face masks were recommended when shopping.^S275,S276^ |
| Barbados | 30 | Mar. 17^S25^ | 24.4 | Masks were required when shopping by Apr. 11,^S26-S28^ and on buses by May 11.^S29^ |

The delay was the number of days from the start of the outbreak until masks were recommended by the government or became widespread due to cultural norms. The estimated start of the outbreak was 5 days before the first infection was reported, or 23 days before the first death (whichever was first).

**Supplemental Table A2. Per-capita COVID-19 Mortality by May 9, Date of Mask Recommendation or Widespread Use Based on Cultural Norms, and Comments on Mask Wear Prevalence (MWP) in Public.**

| **Country.** | **COVID-19 Mortality (per M. pop.) by May 9.** | **Date Masks Recommended or Widely Used by Cultural Norms.** | **Comments on Mask Wear Prevalence (MWP) in Public.** |
| --- | --- | --- | --- |
| Afghanistan | 3.0 |  | Early in the pandemic, masks were noted to be “somewhat common”.^S2^ By March 29, 2020, the Taliban had begun distributing masks to the public.^S1^ |
| Albania | 10.8* | 6/11/2020 |  |
| Algeria | 11.3* | 5/18/2020 |  |
| Andorra | 621.2* | 4/17/2020 |  |
| Angola | 0.1 | 4/23/2020 |  |
| Antigua & Barbuda | 30.6* | 4/5/2020 | Masks were required in all public spaces on April 5.^S12^ |
| Argentina | 6.6* | 4/18/2020 |  |
| Armenia | 14.8* | 5/14/2020 |  |
| Aruba | 28.1* |  |  |
| Australia | 3.8* |  | MWP was 10% by Mar. 15, peaked at 27% by Apr. 19, and was 17% on Jun. 5.^55^ A survey confirmed MWP <25% in March and early April.^54^ Masks were mandated in public in the Melbourne area, effective July 22.^S15^ |
| Austria | 68.3* | 3/30/2020 | A mask mandate in shops was announced on Mar. 30, with the expectation masks would be available by Apr. 1.^S16^ A mask mandate on transit was announced Apr. 6.^S17^ |
| Azerbaijan | 3.1 | 5/1/2020 |  |
| Bahamas | 28.0* | 4/19/2020 |  |
| Bahrain | 4.7* | 4/9/2020 |  |
| Bangladesh | 1.3 | 3/19/2020 | The first death occurred on Mar. 18.^S22^ From Mar. 11-19, 2020, when students age 17 to 28 were asked if they were wearing a surgical face mask in public, 53.8% responded “yes” and an additional 6.6% responded “occasionally”.^S23^ A survey from Mar. 29 to Apr. 29 found that 98.7% reported wearing a face mask in crowded places.^S24^ |
| Barbados | 24.4* | 4/11/2020 | By April 11, cloth face masks were required when shopping.^S26-S28^ Masks were mandatory on buses by May 11.^S29^ |
| Belarus | 13.3* | 6/5/2020 | Masks were recommended on transport on June 5.^S30^ |
| Belgium | 740.4* | 4/24/2020 | From Apr. 4-19, MWP increased from 30 to 37%.^S371^ On Apr. 24, the Prime Minister mandated masks on public transport effective May 4.^S31^ |
| Belize | 5.0* | 5/1/2020 |  |
| Benin | 0.2 | 4/6/2020 | Masks were recommended in public on April 6,^S34^ mandated on April 7,^S35^ and enforced by police beginning April 8.^S36^ |
| Bermuda | 112.4* |  |  |
| Bhutan | 0.0 | 3/11/2020 | On Mar. 11, the Ministry of Health advised wearing of masks in “a crowded place”.^S38^ |
| Bolivia | 9.8* | 5/3/2020 |  |
| Bosnia & Herzegov. | 31.1* | 3/29/2020 | Masks were required in public by March 29.^S42,S43^ |
| Botswana | 0.4 | 5/1/2020 |  |
| Brazil | 50.1* | 4/2/2020 | The first case was reported on Feb. 26.^S45^ The MWP was 25% by Mar. 14, 28% by Mar. 21, 39% by Apr. 4, and 56% by Apr. 12.^54^ On Apr. 2, the Ministry of Health recommended cloth masks when leaving the house.^S46^ |
| British Virgin Is. | 33.1* | 4/28/2020 |  |
| Brunei | 2.3 | 3/22/2020 |  |
| Bulgaria | 13.0* | 3/30/2020 | Masks were recommended in public on Mar. 30.^S50^ |
| Burkina Faso | 2.3 | 4/20/2020 |  |
| Burundi | 0.1 | 7/7/2020 |  |
| Cabo Verde | 3.6* | 5/5/2020 |  |
| Cambodia | 0.0 | 1/28/2020 | Masks were widely used in public by Jan. 28.^S55,S56^ |
| Cameroon | 4.1* | 4/9/2020 | The first cases were identified on Mar. 6.^S59^ From Mar. 10-18, 93.5% of the public viewed masks as protective, and 21.7% had purchased them.S59 In Northern Cameroon, from Mar. 1-28, only 13% wore a mask outside the home.^S60^ From Apr. 1-25, 83.6% wore a mask at gatherings.^S61^ On Apr. 9, masks were mandated beginning Apr. 13.^68,S57,S58^ |
| Canada | 124.3* | 4/6/2020 | Masks were recommended in public on Apr. 6.^S62^ MWP was 6% on Mar. 17, 18% on Apr. 6, 16% on Apr. 13, 31% on Apr. 20, 41% on Apr. 27, 49% on May 26, and 58% on Jun. 11.^55^ In another survey, MWP was <30% in March and early April.^54^ |
| Carib. Netherlands | 0.0 |  |  |
| Cayman Is. | 15.2* | 3/30/2020 |  |
| Central Afric. Rep. | 0.0 | 6/12/2020 |  |
| Chad | 1.9 | 4/13/2020 | On Apr. 13, the president mandated masks (or turban, veil) effective Apr. 14.^68,S67^ On Apr. 14, the government backtracked on enforcement due to lack of supplies.^S70^ Penalties for noncompliance were announced on May 7.^S68^ |
| Channel Is. | 235.8* |  |  |
| Chile | 15.9* | 4/6/2020 | On Apr. 6, the Minister of Health mandated masks on transport, starting Apr. 8.^S71^ The public was invited to make cloth masks.^S71^ |
| China | 3.2 | 1/20/2020 | The first cases had begun by Dec. 1, 2019. By Jan. 20, many in Beijing were wearing masks.^S72^ Masks were required in public in Wuhan on Jan. 22.^S73^ From Jan. 23-25, 30 regions in China mandated masks in public.^60,71^ Masks were ordered throughout China when around others in public on Jan. 31.^S74^ MWP remained between 82% and 90% between Feb. 24 and Jun. 22.^55^ A survey confirmed MWP from 80-90% from Mar. 12 to Apr. 14.^54^ |
| Colombia | 8.7* | 4/4/2020 | On Apr. 3, a reporter in Bogotá noted that 90% of people on the street were wearing masks.^S77^ On Apr. 4, masks were mandated nationally on transport and in shops.^S76-S79^ |
| Congo (Brazzaville) | 1.8 | 4/30/2020 |  |
| Costa Rica | 1.2 | 6/22/2020 |  |
| Croatia | 21.2* | 4/24/2020 |  |
| Cuba | 6.5* | 4/2/2020 |  |
| Curacao | 6.1* |  |  |
| Cyprus | 12.4* | 4/3/2020 |  |
| Czechia | 25.8* | 3/19/2020 | Masks were required in public on March 19.^S86^ |
| Dem. Rep. Congo | 0.4 | 7/21/2020 | In a city not yet affected by the pandemic from Apr. 17 to May 11, 61% were aware of the value of wearing a mask, 27% had worn one since the pandemic began, and 65% felt wearing a mask was difficult.^S89^ |
| Denmark | 90.8* | 8/15/2020 | MWP was ≤10% from Mar. 16 to Jun. 9.^55^ On Aug. 15, masks were mandated on transport, effective Aug. 22.^S87^ |
| Djibouti | 3.0 | 5/10/2020 |  |
| Dominica | 0.0 | 4/9/2020 | The Prime Minister and Health Minister wore masks during a Mar. 30 interview.^S93^ When Dr. Adis King demonstrated mask-wearing to the legislative assembly on Apr. 7, all in attendance wore masks.^S94^ The president recommended masks in public on Apr. 9.^S92^ Others,^S97^ including the state epidemiologist,^S98^ repeated this in coming days. On Apr. 21, a physician estimated MWP of 95%.^S96^ Masks were mandated on transport on Apr. 25.^S95^ |
| Dominican Republic | 35.5* | 4/6/2020 |  |
| Ecuador | 97.3* | 4/7/2020 | The first case was reported on Feb. 29 in a traveler arriving on Feb. 14.^S101^ The first death was reported on Mar. 13.^S102^ By Apr. 3, in Guayaquil, mortuary facilities were overwhelmed, and bodies were left on the streets.^S103^ On Apr. 7, the Interior Minister mandated masks in public.^S104^ |
| Egypt | 5.0* | 5/31/2020 | In Mar. 2020, 76.4% of adults understood the value of wearing a mask in public, but only 36.4% agreed that they did so.^S107^ By Mar. 20, mask prices soared, and volunteer organizations advocated public masking.^S105^ Masks were mandated in public on May 31.^S106^ |
| El Salvador | 2.6 | 4/4/2020 | The first death was reported Mar. 31. President Bukele recommended universal mask wear on Apr. 4.^S109^ Masks were mandated in San Salvador on Apr. 7.^S110^ On Apr. 11, the president mandated masks, effective Apr. 14.^S111^ |
| Equatorial Guinea | 2.9 | 4/14/2020 |  |
| Eritrea | 0.0 |  |  |
| Estonia | 45.2* | 4/5/2020 |  |
| Eswatini | 1.7 | 5/30/2020 |  |
| Ethiopia | 0.0 | 4/11/2020 | From Mar. 2-Apr. 10, 75.7% of chronic disease patients agreed that it was important to wear a mask outside the home.^S116^ From Mar. 20-24, 87% of the public believed wearing a mask could prevent spread of the virus, but only 14% had done so recently.^S117^ From Apr. 1-15, in southern Ethiopia, 84% believed wearing a mask was protective, 160 respondents (36%) had been to a crowded place recently, and 129 respondents (29%) had worn a mask when leaving home recently.^S118^ Masks were mandated in public on Apr. 11.^S115^ In a survey from Apr. 15-22, 84% believed a mask could provide protection from coronavirus, 137 people (40%) had gone to a crowded place after the onset of the pandemic, and 82 people (24%) had worn a mask outside the home.^S119^ |
| Faeroe Islands | 0.0 |  |  |
| Falkland Islands | 0.0 |  |  |
| Fiji | 0.0 |  |  |
| Finland | 47.8* | 4/14/2020 | Masks were worn by ≤10% of the public from Mar. 16 to Jun. 9.^55^ Public mask wear was recommended on Apr. 14.^S120^ |
| France | 403.1* | 4/3/2020 | The first case was reported on Jan. 24,^S121^ and the first death on Feb. 14, of a man who arrived on Jan. 16.^S122^ MWP was 5% on Mar. 10, 22% on Mar. 27, 25% on Apr. 3, 38% on Apr. 10, 43% on Apr. 17, 56% on May 1, 76% on May 20, and 75% on June 12.^55^ MWP <50% in early April was confirmed in a survey.^54^ On Apr. 3, the Académie Nationale de Médecine recommended masks in public.^S123^ On May 7, nationwide, including overseas departments, masks were mandated on transport, starting May 11.^S370^ |
| French Polynesia | 0.0 |  |  |
| Gabon | 3.6 | 4/15/2020 |  |
| Gambia | 0.4 | 7/17/2020 | The public widely sought masks early in the outbreak.^S124,S125^ |
| Georgia | 2.5 | 4/17/2020 |  |
| Germany | 90.1* | 4/1/2020 | The first case was reported Jan. 27. The patient had contact with a colleague from China beginning Jan. 19.^S128^ On Mar. 31, masks were mandated in Jena.^S129^ The Robert Koch Institute recommended masks in public on April 1.^S130^ MWP was 7% on Mar. 30, 14% on Apr. 6, 17% on Apr. 13, 24% on Apr. 20, and 62%-64% from May 4 to June 18.^55^ A survey confirmed MWP ≤20% in March and early April.^54^ All German states mandated mask wear in public by Apr. 22.^S131^ |
| Ghana | 0.7 | 4/19/2020 | From Mar. 27-29, in 43 transport stations, masks were worn by many people at 1 station, and by a few people at 27 stations.^S134^ On Apr. 19, the president mandated masks in public.^S132,S133^ |
| Gibraltar | 0.0 |  |  |
| Greece | 14.5* | 4/27/2020 |  |
| Greenland | 0.0 |  |  |
| Grenada | 0.0 | 4/3/2020 | On Apr. 3, the Ministry of Health recommended all wear a mask to “prevent asymptomatic people from transmitting the disease”.^S137^ Masks were mandated outside the home on Apr. 6.^S138^ |
| Guatemala | 1.3 | 4/9/2020 |  |
| Guinea | 0.8 | 4/13/2020 |  |
| Guinea-Bissau | 1.5 | 5/11/2020 |  |
| Guyana | 12.7* | 4/9/2020 |  |
| Haiti | 1.1 | 5/4/2020 |  |
| Honduras | 10.8* | 4/6/2020 |  |
| Hong Kong | 0.5 | 1/24/2020 | Surgical masks were traditionally used, and recommended on public transport and in crowded places, on Jan. 24.^16,S146^ MWP was 73% the week of Jan. 21, and 98% from mid-February to May.^S148^ In Feb. 2020, 95% of pedestrians were observed to wear masks, and 94% believed mass masking controls the outbreak.^S149^ A poll found MWP ≥85% from Feb. 25 to Jun. 22.^55^ |
| Hungary | 41.9* | 4/29/2020 |  |
| Iceland | 29.3* | 7/30/2020 | On Jul. 30, masks were mandated on transit, effective Jul. 31.^S151^ |
| India | 1.5 | 4/4/2020 | The first case was diagnosed on Jan. 30.^S152^ The Health Ministry recommended homemade face masks on Apr. 4.^S153^ MWP was 60% from Mar. 12-14, 67% from Mar. 19-21, and 73%-76% from Mar. 26 to Apr. 12.^54^ Another poll showed MWP of 43% on Mar. 16, 46% on Mar. 20, 65% on Mar. 27, 71% on Apr. 3, 79% on Apr. 10, and 81-84% from Apr. 17 to May 1.^55^ In March, 75% of the public agreed masks should be worn by asymptomatic people, and 77% agreed the N95 mask was most protective.^S154^ |
| Indonesia | 3.5 | 2/24/2020 | The first death occurred on Mar. 3.^S157^ The public scrambled to buy face masks in early February.^S155^ MWP was 54% on Feb. 24, 47% on Mar. 9, 59% on Mar. 23, 71% on Mar. 30, 79% on Apr. 13, 81% on Apr. 20, and from 82%-84% from May 4 to Jun. 9.^55^ During March and April, 76% of students wore a mask outside the home.^S159^ Masks were mandated in public on Apr. 5.^S158^ |
| Iran | 78.4* | 3/29/2020 | The first reported infections were two deaths announced on Feb. 19.^S160^ By Mar. 12, satellite imagery demonstrated mass graves in Qom.^S161^ Many, even within the government, have questioned the official mortality figures.^S162,S164,S165^ The Iranian Health Ministry on Mar. 24 recommended masks only if symptomatic or caring for the sick (personal communication, Linnea I. Laestadius, June 7, 2020).^14^ Masks in gyms, parks, and public transit were recommended by the Ministry by Mar. 29.^14^ A survey from Feb. 25 to Apr. 25 found 64% of the public wore a mask and gloves in crowded places.^S163^ |
| Iraq | 2.7 | 4/19/2020 |  |
| Ireland | 292.8* | 5/15/2020 |  |
| Isle of Man | 270.5* |  |  |
| Israel | 28.5* | 4/1/2020 |  |
| Italy | 502.7* | 4/28/2020 | The first cases were reported on Jan. 31, among a family who arrived on Jan. 23.^S169^ By Mar. 10, all intensive care beds were taken in Lombardy, and not have enough ventilators were available.^S170^ MWP was 26% on Mar. 11, 59% on Mar. 19, and 85-89% from Apr. 16 to Jun. 10.^55^ A poll confirmed MWP >50% for the first time from Mar. 19-21.^54^ Lombardy (Apr. 5) and Tuscany (Apr. 6) required masks in April.^S171^ An Apr. 28 nationwide mask mandate in shops and transport was effective May 4.^S172^ |
| Ivory Coast | 0.8 | 4/4/2020 | On Apr. 4, health officials recommended masks in public.^68,S174^ |
| Jamaica | 3.0 | 4/8/2020 |  |
| Japan | 4.8* | 1/16/2020 | Public use of masks is traditional.^16^ In winter, 64% of adults habitually wore a mask.^17^ Public masking was manifest by Jan. 16 when the first case was announced.^S177-S179^ The government recommended masks in “confined, badly ventilated spaces”.^16^ MWP was >60% by Mar. 14, >75% by Apr. 12.^54^ A poll found MWP of 62% by Mar. 17, 76% by Apr. 13, 81% by Apr. 20, and 86% by May 4.^55^ |
| Jordan | 0.9 | 4/27/2020 | From Mar. 19-21, MWP was 39.8% among university students.^S182^ King Abdullah recommended masks when shopping on Apr. 27.^S180^ |
| Kazakhstan | 1.7 | 5/26/2020 |  |
| Kenya | 0.6 | 4/2/2020 | The first case announced Mar. 12 had arrived on Mar. 5.^S184^ The first death was on Mar. 26, of a man who arrived on Mar. 13.^S185^ Masks were mandated on transport on Apr. 2, and more broadly in public on Apr. 4.^S186-S187^ On May 5, 89% had worn a mask in the previous week, and 73% always did so outside the home.^S188^ |
| Kuwait | 11.5* | 3/23/2020 |  |
| Kyrgyzstan | 1.8 | 5/10/2020 |  |
| Laos | 0.0 | 3/6/2020 | Health officials in Laos advised mask-wearing by Mar. 6.^S190^ The public began wearing masks before any cases were reported in the country.^S191^ |
| Latvia | 9.5* | 4/27/2020 |  |
| Lebanon | 3.8* | 4/25/2020 | The first case was reported Feb. 21.^S193^ Masks were popular among the public from mid-March to early April,^S194,S195^ and were recommended by the health minister on Apr. 25.^S195^ |
| Liberia | 4.0* | 4/24/2020 |  |
| Libya | 0.4 | 4/16/2020 |  |
| Liechtenstein | 26.2* | 5/15/2020 |  |
| Lithuania | 18.0* | 3/26/2020 | Masks were recommended Mar. 26,^S200^ and mandated on Apr. 8.^S201^ |
| Luxembourg | 161.3* | 4/15/2020 |  |
| Macao | 0.0 | 1/23/2020 | Mask use is traditional. By Jan. 23, the government had implemented a mask distribution program.^S203^ |
| Madagascar | 0.0 | 4/20/2020 |  |
| Malawi | 0.2 | 4/4/2020 | The first death was on Apr. 7.^S205^ The public was required to wear masks on Apr. 4.^S206^ In Karonga, from Apr. 25 to May 23, MWP was 22% in urban areas, and 5% in rural areas.^S207^ |
| Malaysia | 3.3 | 1/30/2020 | Masks were used by the public by Jan. 30.^S208^ MWP was 55% on Feb. 24, 69% on Mar. 23, 82% on Apr 6, and 85-88% from May 4 to Jun. 8.^55^ |
| Maldives | 5.5* | 5/19/2020 |  |
| Mali | 1.8 | 5/10/2020 |  |
| Malta | 11.3* | 5/1/2020 |  |
| Mauritania | 0.2 | 5/6/2020 |  |
| Mauritius | 7.9* | 3/31/2020 |  |
| Mayotte | 40.3* | 5/11/2020 |  |
| Mexico | 26.0* | 5/5/2020 | The MWP was 17% on Mar. 17 , 37% on Apr. 6, 46% on Apr. 13, 60% on Apr. 20, and 67% on Apr. 27.^55^ A poll found MWP of 31% by Mar. 14, 36% by Mar. 21, 46% by Apr. 4, and 58% by Apr. 9.^54^ Although some states mandated masks earlier, the relevant federal minister recommended masks on May 5.^S215,S216^ |
| Moldova | 39.9* | 5/7/2020 |  |
| Mongolia | 0.0 | 1/31/2020 | The public began wearing masks in January.^S218^ The mayor of Ulaanbaatar ordered organizations to implement mask-wearing on Jan. 27, 2020.^S219^ Public mask wearing was common by mid-February when transport service was denied for those without a mask.^S220^ |
| Montenegro | 12.7* | 4/30/2020 |  |
| Montserrat | 200.3* | 4/29/2020 |  |
| Morocco | 5.0* | 4/6/2020 |  |
| Mozambique | 0.0 | 4/4/2020 | Masks were recommended by health authorities on Apr. 4,^S225^ and required on transport or in gatherings on Apr. 8.^S226^ |
| Myanmar | 0.1 | 4/5/2020 | The first death occurred on Mar. 31.^S228^ From Mar. 3-20, 72% of adults were confident they would wear a surgical mask when visiting a crowded area.^S233^ On Apr. 5, the Ministry of Health recommended masks in crowded places.^S230^ On Apr. 7, State Counsellor Daw Aung San Suu Kyi announced that she would make a mask for herself.^S231^ By Apr. 16, some regions mandated masks in public.^S232^ A Ministry of Health survey from May 7-23, found a MWP of 80%.^S229^ |
| Namibia | 0.0 | 5/2/2020 |  |
| Nepal | 0.0 | 3/25/2020 | Facemasks are traditionally seen in urban centers due to air pollution.^S240^ The first case of COVID-19 was reported on Jan. 13, in a traveler from Wuhan.^S241^ No subsequent cases were reported until the second week of March.^S241^ By Jan. 29, all students at some schools were wearing masks.^S235^ By Feb. 3, pharmacies were selling out of masks.^S236^ Tailors began sewing cloth masks.^S240^ By Feb. 8, “a majority” of the public was wearing masks.^S237^ The recommendation to wear masks in public was formalized on Mar. 25.^S238^ The Ministry of Health distributed masks to children and elderly in shelters by Mar. 25.^S239^ The fraction agreeing that asymptomatic people should wear masks was 83% at the end of March,^S241^ and 96% from May 15-Jun. 20.^S242^ |
| Netherlands | 316.4* | 5/6/2020 | From Apr. 1-19, MWP was 7%.^S371^ On May 6, the prime minister mandated masks on transport, effective Jun. 1.^S243^ |
| New Caledonia | 0.0 | 4/30/2020 |  |
| New Zealand | 4.4* | 8/6/2020 | The government announced on Aug. 6, 2020 that in the event of community transmission, public use of masks would be appropriate.^S244^ |
| Nicaragua | 0.8 |  |  |
| Niger | 1.9 | 5/12/2020 |  |
| Nigeria | 0.6 | 4/14/2020 | Masks were required in public on Apr. 14.^S249,S248^ From May 7-18, 65% of had worn a mask outside the home recently.^S250^ |
| North Macedonia | 43.7* | 4/23/2020 |  |
| Norway | 40.4* | 8/14/2020 | MWP was ≤10% from Mar. 16 to Jun. 9.^55^ On Aug. 14, masks were recommended (but not mandated) on transport.^S252^ |
| Oman | 3.3 | 5/18/2020 |  |
| Pakistan | 2.9 | 5/31/2020 | In March 2020, 78% in Sargodha favored wearing a mask.^S255^ From Apr. 1-12, 80% supported a mask mandate when outside the home.^S256^ Masks were mandated in crowded spaces on May 31.^S254^ |
| Palestine | 0.4 | 5/5/2020 |  |
| Panama | 54.9* | 4/7/2020 |  |
| Papua New Guinea | 0.0 | 4/24/2020 |  |
| Paraguay | 1.4 | 4/7/2020 |  |
| Peru | 55.0* | 4/3/2020 |  |
| Philippines | 6.4* | 1/30/2020 | Masks were used extensively as early as Jan. 30.^S262^ In a poll, 60% indicated wearing a mask in public on Feb. 24, 76% on Mar. 23, and 81%-84% from Mar. 30 through Jun. 22.^55^ Masks were mandated on Apr. 2. |
| Poland | 20.7* | 4/9/2020 | On Apr. 9, masks were mandated in public effective Apr. 16, and mask vending machines were installed.^S263^ From Apr. 12-14, 60.4% of students age 18-27 wore a mask in the previous 7 days.^S264^ |
| Portugal | 110.4* | 4/27/2020 |  |
| Qatar | 4.5* | 4/22/2020 |  |
| Réunion | 0.0 | 5/7/2020 |  |
| Romania | 48.8* | 4/22/2020 |  |
| Russia | 12.5* | 5/11/2020 | The first cases were reported on Jan. 31.^S268^ The MWP was 11% by Mar. 14, 19% by Mar. 21, 36% by Mar. 28, 57% by Apr. 4, and 59% by Apr. 12.^54^ On May 11, masks were mandated in shops and transport.^S269^ |
| Rwanda | 0.0 | 4/18/2020 |  |
| Saint Kitts & Nevis | 0.0 | 4/2/2020 | On Apr. 2, Chief Medical Officer Dr. Hazel Laws recommended wearing a mask in public on the grounds that masks could block droplets, and viral particles could remain suspended for 3 hours.^S272^ Masks were mandated in public on Apr. 7.^S273^ |
| Saint Lucia | 0.0 | 4/7/2020 | Masks were recommended when shopping by the chief medical officer on Apr. 7.^S275,S276^ |
| San Marino | 1208.3* | 4/17/2020 |  |
| São Tomé & Príncipe | 22.8* | 4/22/2020 | On Apr. 22, masks were mandated in public beginning Apr. 24.^65^ |
| Saudi Arabia | 6.9* | 4/28/2020 | The first case was announced Mar. 2.^S279^ MWP was 35% on Mar. 18, 54% on Apr. 1, 59% on Apr. 14, 63% on May 4, and 72% on Jun. 3.^55^ From Apr. 2-5, during lockdown, 16.9% had worn a mask even without symptoms.^S282^ Public mask-wearing was recommended on Apr. 28,^S280^ and mandated on May 30.^S281^ From May 28 to Jun. 30, 87% of nursing students had recently worn a mask when going out.^S283^ |
| Senegal | 1.0 | 4/17/2020 |  |
| Serbia | 24.4* | 4/29/2020 | Masks were mandated in gyms and salons on Apr. 29.^S284^ In April 2020, 60% of the public were willing to wear a mask during a pandemic, and when asked if they wore masks, the mean response was 3.25 (SD 1.6) on a 1 to 5 scale (where 4=“agree”, 5=“strongly agree”).^S285^ |
| Seychelles | 0.0 | 6/9/2020 |  |
| Sierra Leone | 2.3 | 4/1/2020 | Masks were recommended in public on Apr. 1.^S288^ Compliance has been incomplete.^S289^ |
| Singapore | 3.4 | 4/3/2020 | On Apr. 3, the government stopped discouraging mask-wearing by the public, and distributed masks.^S290-S292^ MWP was 27% on Mar. 27, 37% on Apr. 3, 73% on Apr. 10, 85% on Apr. 17, and 90% from Apr. 24 to Jun. 19.^55^ |
| Sint Maarten | 349.8* | 4/18/2020 |  |
| Slovakia | 4.8* | 3/15/2020 | Masks were mandated in shops and transit on Mar. 15,^S294^ and more broadly in public on Mar. 25.^S295^ |
| Slovenia | 48.6* | 3/29/2020 | Masks were mandated in indoor public spaces by Mar. 29.^S296^ |
| Somalia | 3.0 |  | MWP was 51% from Apr. 23-May 7, and 56% from Jul. 7-29.^S297^ |
| South Africa | 3.1 | 4/10/2020 | From Apr. 8-24, 86% agreed masks could prevent infection.^S298^ Health officials recommended masks in public on Apr. 10.^S299^ |
| South Korea | 5.0* | 1/30/2020 | Use of masks is traditional.^16^ The alert level was raised from yellow to orange on Jan. 27.^60^ Children were advised to wear masks at school by Jan. 30.^S301^ By Feb. 2, mask sales increased 373 times year-over-year.^60^ Stores were selling out of masks by Feb. 3.^S302^ A superspreader event in mid-February was associated with a religious group which did not use masks at their gatherings.^61^ An initial mask shortage was alleviated at the end of February when the government began to control mask distribution.^S303^ On Feb. 22, the government instructed the wearing of masks in the epidemic area.^60^ |
| South Sudan | 0.0 | 4/29/2020 | On Apr. 29, the High Level Task Force approved the use of locally-manufactured cloth masks in public.^S305^ |
| Spain | 566.3* | 4/11/2020 | The first case was reported on Jan. 31,^S306^ and the first death on Feb. 13.^S307^ MWP was 5% on Mar. 12, 25% on Mar. 19, 42% on Mar. 25, 56% on Apr. 8, 65% on Apr. 16, 72% on Apr. 30, and from 84%-87% from May 20-Jun. 12.^55^ Masks were mandated in transit on Apr. 11.^S308^ A poll found MWP of 50% by Mar. 21, 53% by Apr. 4, and 61% by Apr. 12.^54^ |
| Sri Lanka | 0.4 | 4/11/2020 | The public bought masks at the end of January.^S309^ Masks were mandated in public on Apr. 11.^S310^ |
| St. Vincent & Gren. | 0.0 | 4/26/2020 |  |
| Sudan | 1.5 | 3/16/2020 | The first death occurred on Mar. 12. Masks were dispensed by pharmacists for free by Mar. 16.^S313,S314^ From Mar. 25 to Apr. 4, of 2336 adults, 703 (30.1%) had been to a crowded area, and 1153 (49.4%) had worn a mask outside the home in the previous few days.^S315^ |
| Suriname | 1.7 | 5/31/2020 |  |
| Sweden | 318.8* |  | MWP was ≤10% from Mar. 16 to Jun. 9.^55^ |
| Switzerland | 211.4* | 6/15/2020 | The chief of the Communicable Diseases Department recommended masks on transport on Jun. 15.^S317^ By Jun. 18, 6% of transport riders did so.^S317^ |
| Syria | 0.2 |  | From Mar. 19-21, 52% of university students agreed masks should be worn outside, and 25% did so at least sometimes.^S318^ |
| Taiwan | 0.3 | 1/27/2020 | Use of masks is traditional. By Jan. 24, Taiwan banned the export of surgical masks.^S320,S321^ By Jan. 27, the government limited mask exports and sales from pharmacies to those for personal use.^S319^ On Jan. 28, the government began releasing 6 million masks daily, with each resident able to purchase 3 masks weekly.^S320^ MWP was >80% from Feb. 25 to Jun. 22.^55^ |
| Tanzania | 0.4 |  |  |
| Thailand | 0.8 | 1/28/2020 | Masks, including N95 masks, are traditionally worn outdoors to combat smog.^S322^ The government handed out masks and recommended masks in public to prevent coronavirus by Jan. 28.^S323-S326^ The recommendation of cloth masks for the public was reaffirmed by the Ministry of Public Health on Mar. 3.^S327^ Enforcement of a mask mandate on transport began Mar. 26.^S327^ MWP was 73% by Feb. 24, 80% by Mar. 23, and 84-89% from Mar. 30 to Jun. 22.^55^ During March 2020, a survey found masks were worn “all the time” by 14% of COVID-19 cases and 24% of controls, and “some of the time” by 38% of cases and 15% of controls.^S327^ |
| Timor-Leste | 0.0 | 3/28/2020 | Masks were required in stores and other venues on Mar. 28.^S329^ |
| Togo | 1.2 | 4/19/2020 |  |
| Trinidad & Tobago | 5.7* | 4/5/2020 |  |
| Tunisia | 3.8* | 4/6/2020 |  |
| Turkey | 44.3* | 4/3/2020 |  |
| Turks and Caicos | 25.8* | 4/30/2020 |  |
| Uganda | 0.0 | 5/1/2020 |  |
| Ukraine | 8.6* | 3/30/2020 | Masks were recommended in public on Mar. 30.^S337,S338^ |
| United Arab Emir. | 18.7* | 3/27/2020 | The first cases were reported Jan. 29.^S339^ By Feb. 29, mask usage had become “more prominent”, but the Ministry of Health and Community Protection advised that N95 masks should be reserved for medical personnel, and could cause “respiratory illness” if worn by the public.^S340^ On Mar. 27, masks were mandated when indoors.^S341^ MWP was 39% on Mar. 18, 44% on Mar. 25, 63% on Apr. 1 and 78%-81% from Apr. 14-Jun. 17.^55^ |
| United Kingdom | 465.3* | 5/11/2020 | The first cases were reported on Jan. 31.^S342^ MWP was 2% by Mar. 20, 11% by Apr. 17, 20% by May 1, and 27% by Jun. 17.^55^ A survey found MWP <20% from Mar. 12-Apr. 12.^54^ In England, masks were recommended on transport and in shops on May 11,^S343^ and mandated on transport on Jun. 4, effective Jun. 15.^S344^ |
| United States | 241.8* | 4/3/2020 | The first case arrived on Jan. 15, and sought care on Jan. 19.^S347^ The U.S. C.D.C. recommended public mask wear on the evening of April 3.^S348^ The MWP was 7% on Mar. 2, 5% on Mar. 17, 17% on Mar. 30, 29% on Apr. 6, 49% on Apr. 13, 58% on Apr. 20, 63% on Apr. 27, 68% on May 26, and 66% on Jun. 8.^55^ In a survey, MWP was 32% from Apr. 2-4, and 50% from Apr. 9-12.^54^ In a survey, from Apr. 14-20, 36% of adults always wore a mask outside the home, and 32% sometimes did.^S345^ In Vermont, from May 16-30, 76% of people entering businesses actually wore a mask.^S349^ In Wisconsin from June 3-9, 42% of shoppers actually wore a mask.^S350^ In a 3-county area in Colorado, actual weekly mask wear from Apr. 26-Jul. 12 ranged from 56%-90%. After a Jul. 17 state mask mandate, MWP from Jul. 19 to Aug. 2 was 88%-97%.^S346^ |
| Uruguay | 5.2* | 4/10/2020 |  |
| Uzbekistan | 0.3 | 3/25/2020 | The first death was on Mar. 29. Masks were mandated on Mar. 25.^S353^ |
| Venezuela | 0.4 | 3/13/2020 | The first death was announced on Mar. 26.^S357^ The president demonstrated wearing of masks on Mar. 13.^S354,S355^ Masks were required in any public space by Mar. 17.^S356,S357^ |
| Vietnam | 0.0 | 1/27/2020 | Masks were widely used by Jan. 27,^S359,S360^ and mandated on Mar. 16. MWP was 85-90% from Mar. 12 to Apr. 14.^54^ A poll found MWP of 59% on Mar. 23, and 79%-87% from Mar. 30-Jun. 8.^55^ From Mar. 31-Apr. 6, 99.5% used a mask when outside.^S363^ |
| Yemen | 0.2 |  | The niqab is worn by 90% of women.^S365^ Local doctors believe it might reduce transmission of the virus.^S365^ |
| Zambia | 0.4 | 4/4/2020 | The first death was recorded on Apr. 2. On Apr. 4, masks were recommended for the public “at all times” by the Zambian Minister of Health.^S366^ This spurred the manufacture of cloth masks.^S367^ On Apr. 16, masks were mandated in public.^S368^ |
| Zimbabwe | 0.3 | 5/1/2020 |  |

* Denotes per-capita mortality above the median value (high-mortality countries).

**Supplemental Table A3. Predictors of (log) Country-wide Per-capita Coronavirus Mortality by May 9 by Univariate (Unadjusted) Linear Regression in 200 Countries.**

|  | 10^coefficient^ | Coefficient (SE) | 95% CI | p value |
| --- | --- | --- | --- | --- |
| Duration of outbreak (weeks) | 1.4291 | 0.155 (0.030) | 0.097 to 0.213 | <0.001 |
| Duration of outbreak without masks (weeks) | 1.7800 | 0.250 (0.028) | 0.194 to 0.307 | <0.001 |
| Duration of outbreak before international travel restrictions (weeks) | 1.5329 | 0.186 (0.035) | 0.116 to 0.255 | <0.001 |
| Duration of outbreak without lockdown (weeks) | 1.6123 | 0.207 (0.039) | 0.131 to 0.284 | <0.001 |
| Temperature, mean (C) | 0.8821 | -0.054 (0.008) | -0.071 to -0.038 | <0.001 |
| Urban population (%) | 1.0478 | 0.0203 (0.003) | 0.014 to 0.027 | <0.001 |
| GDP per capita ($1000) | 1.0432 | 0.0184 (0.003) | 0.012 to 0.025 | <0.001 |
| Age 14 & under (% of pop.) | 0.8748 | -0.058 (0.007) | -0.072 to -0.044 | <0.001 |
| Age 60 & over (% of pop.) | 1.1963 | 0.078 (0.009) | 0.061 to 0.095 | <0.001 |
| Surface area (million km^2^) | 1.1357 | 0.055 (0.044) | -0.032 to 0.143 | 0.21 |
| Population (million) | 1.0003 | 0.00012 (0.0006) | -0.001 to 0.001 | 0.84 |
| Prevalence males (%) | 0.9757 | -0.011 (0.025) | -0.061 to 0.039 | 0.67 |
| Smoking prevalence, adult (%) | 1.0531 | 0.022 (0.010) | 0.002 to 0.043 | 0.03 |
| Obesity prevalence, adult (%) | 1.1273 | 0.052 (0.008) | 0.036 to 0.068 | <0.001 |
| Tests per cap. (log) by May 9 | 3.6901 | 0.567 (0.075) | 0.419 to 0.715 | <0.001 |

Durations run from the estimated date of first infection in the country until 23 days before May 9, 2020 (i.e. April 16), or the stated event (mask recommendation or lockdown). Obesity data available for 196 countries. Testing data available for 183 countries by May 9.

**Supplemental Table A4. Predictors of (log) Country-wide Per-capita Coronavirus Mortality by May 9 by Multivariable Linear Regression in 200 Countries.**

|  | 10^coefficient^ | Coefficient (SE) | 95% CI | P |
| --- | --- | --- | --- | --- |
| Duration in country (weeks) | 1.5980 | 0.2036 (0.037) | 0.131 to 0.277 | <0.001 |
| Time wearing masks (weeks) | 0.6860 | -0.1636 (0.030) | -0.223 to -0.104 | <0.001 |
| Time since start of international travel restrictions (weeks) | 0.8516 | -0.0698 (0.035) | -0.139 to 0.0002 | 0.049 |
| Time in internal lockdown (weeks) | 1.0194 | 0.00833 (0.051) | -0.093 to 0.109 | 0.87 |
| Population, age≥60 (%) | 1.1367 | 0.0556 (0.010) | 0.035 to 0.076 | <0.001 |
| Urbanization (%) | 1.0188 | 0.00808 (0.003) | 0.002 to 0.014 | 0.008 |
| Temperature (C) | 0.9989 | -0.00046 (0.009) | -0.018 to 0.017 | 0.96 |
| Constant | -- | -7.66 (0.395) | -8.44 to -6.88 | <0.001 |

Duration of outbreak in country from estimated date of first infection until 23 days before May 9, 2020 (i.e. April 16). Mask and lockdown durations run from the stated event (mask recommendation or lockdown) or estimated date of first infection in the country (whichever was later) until 23 days before May 9, 2020 (i.e. April 16). Model r^2^=0.481.

**Supplemental Table A5. Predictors of (log) Country-wide Per-capita Coronavirus Mortality by May 9 by Multivariable Linear Regression in 179 Countries.**

|  | 10^coefficient^ | Coefficient (SE) | 95% CI | P |
| --- | --- | --- | --- | --- |
| Duration in country (weeks) | 1.6253 | 0.211 (0.038) | 0.136 to 0.286 | <0.001 |
| Time wearing masks (weeks) | 0.7357 | -0.133 (0.032) | -0.197 to -0.069 | <0.001 |
| Time in lockdown (weeks) | 0.9865 | -0.0059 (0.054) | -0.113 to 0.102 | 0.91 |
| International travel controls (time since start, weeks) | 0.8672 | -0.0619 (0.038) | -0.136 to 0.012 | 0.10 |
| Population, % age 60 or over | 1.0909 | 0.0378 (0.012) | 0.015 to 0.061 | 0.001 |
| Urbanization (%) | 1.0135 | 0.00580 (0.004) | -0.002 to 0.013 | 0.13 |
| Obesity prevalence (%) | 1.0399 | 0.0170 (0.010) | -0.002 to 0.036 | 0.09 |
| Temperature (C) | 0.9823 | -0.0078 (0.009) | -0.026 to 0.010 | 0.40 |
| Testing (log per cap., by May 9) | 1.2514 | 0.0974 (0.116) | -0.131 to 0.325 | 0.40 |
| Constant | -- | -7.319 (0.623) | -8.55 to -6.09 | <0.001 |

Based on 179 countries with both obesity and testing data by May 9. Duration of outbreak in country from estimated date of first infection until 23 days before May 9, 2020 (i.e. April 16). Mask and lockdown durations run from the stated event (mask recommendation or lockdown) or estimated date of first infection in the country (whichever was later) until 23 days before May 9, 2020 (i.e. April 16). Model r^2^=0.523.

**Supplemental Table A6. Predictors of (log) Country-wide Per-capita Coronavirus Mortality by May 9 by Multivariable Linear Regression in 200 Countries, with Mask Wear Determined by Recommendations and Surveys (When Available).**

|  | 10^coefficient^ | Coefficient (SE) | 95% CI | P |
| --- | --- | --- | --- | --- |
| Duration in country (weeks) | 1.5993 | 0.204 (0.037) | 0.131 to 0.277 | <0.001 |
| Time wearing masks (weeks) | 0.6836 | -0.165 (0.030) | -0.224 to -0.106 | <0.001 |
| Time in lockdown (weeks) | 0.9021 | 0.0090 (0.051) | -0.092 to 0.110 | 0.86 |
| Time since start of international travel controls (weeks) | 0.8529 | -0.0691 (0.035) | -0.139 to 0.0004 | 0.051 |
| Population, age≥60 (%) | 1.1367 | 0.0556 (0.010) | 0.035 to 0.076 | <0.001 |
| Urbanization (%) | 1.0185 | 0.00796 (0.003) | 0.002 to 0.014 | 0.009 |
| Temperature | 0.9988 | -0.00052 (0.009) | -0.018 to 0.017 | 0.95 |
| Constant | -- | -7.658 (0.393) | -8.434 to -6.882 | <0.001 |

` Duration of outbreak in country from estimated date of first infection until 23 days before May 9, 2020 (i.e. April 16). Mask and lockdown durations run from the stated event (mask recommendation or lockdown) or estimated date of first infection in the country (whichever was later) until 23 days before May 9, 2020 (i.e. April 16). Model r^2^=0.483.

**Supplemental References by Country**.

**Afghanistan**.

S1. Smith SS. Service Delivery in Taliban-Influenced Areas of Afghanistan. United States Institute of Peace. April 2020. Available from: <https://www.usip.org/sites/default/files/2020-04/20200430-sr_465-_service_delivery_in_taliban_influenced_areas_of_afghanistan-sr.pdf> Accessed June 28, 2020.

S2. Mousavi SH, Abdi M, Zahid SU, Wardak K. Coronavirus disease 2019 (COVID-19) outbreak in Afghanistan: Measures and challenges. Infect Control Hosp Epidemiol. 2020 May 15 : 1–2.

**Albania**.

S3. Albania’s prime minister threatens return to lockdown: ‘The economy falls and rises. The dead are not resurrected.’ Associated Press. June 12, 2020. Available from: <https://www.marketwatch.com/story/albanias-prime-minister-threatens-return-to-lockdown-the-economy-falls-and-rises-the-dead-are-not-resurrected-2020-06-12> Accessed August 8, 2020.

**Algeria**.

S4. No author listed. COVID-19: Algeria imposes wearing of masks. Africa News. May 19, 2020. Available from: <https://www.africanews.com/2020/05/19/covid-19-algeria-imposes-wearing-of-masks/> Accessed August 1, 2020.

**Andorra**.

S5. Ignacio P. En Andorra ya se permite hacer ejercicio: mascarilla obligatoria y el ciclismo sigue prohibido. Brujulabike. April 18, 2020. Available from: <https://www.brujulabike.com/andorra-permite-ejercicio-mascarilla-obligatoria-prohibido-ciclismo/> Accessed July 6, 2020.

**Angola**.^S373^

S6. No author listed. Angola: COVID-19 - Angola Detects Seven New Positive Cases. All Africa. May 9, 2020. Available from: <https://allafrica.com/stories/202005110170.html> Accessed July 6, 2020.

S7. No author listed. Angola reports first two confirmed COVID-19 cases. Xinhua. March 21, 2020. Available from: <http://www.china.org.cn/world/Off_the_Wire/2020-03/21/content_75842926.htm> Accessed July 7, 2020.

S8. No author listed. Covid-19 faz as primeiras duas vitimas em Angola. Platina Line. March 29, 2020. Available from: <https://web.archive.org/web/20200330145319/http://platinaline.com/covid-19-as-primeiras-duas-vitimas-angola/> Accessed July 7, 2020.

S9. No author listed. Covid-19: Use of a mask becomes mandatory. Agencia Angola Press. April 16, 2020. Available from:

<http://www.angop.ao/angola/en_us/noticias/saude/2020/3/16/Covid-Use-mask-becomes-mandatory,a665b68b-834b-4758-b3c5-b856a7854911.html> Accessed July 7, 2020.

S10. No author listed. Angola: Authorities extend state of emergency to May 10. Garda. April 24, 2020. <https://www.garda.com/crisis24/news-alerts/336006/angola-authorities-extend-state-of-emergency-to-may-10-update-6> Accessed July 7, 2020.

**Antigua & Barbuda**.

S11. De Shong D. Antigua and Barbuda records first case of the novel coronavirus. Loop News Barbados. March 13, 2020. Available from: <http://www.loopnewsbarbados.com/content/antigua-and-barbuda-records-first-case-coronavirus-4> Accessed June 17, 2020.

S12. No author listed. Mandatory wearing of face masks will soon be enforceable. Antigua Nice. April 6, 2020. Available from: <http://www.antiguanice.com/v2/client.php?id=943&news=12425> Accessed May 29, 2020.

**Argentina**.

S13. No author listed. Argentina: Health Alert. US Embassy in Argentina. April 18, 2020. Available from: <https://ar.usembassy.gov/argentina-health-alert-8/> Accessed July 6, 2020.

**Armenia**.

S14. Harutyunyan A. Wearing face masks in public transport to be compulsory in Armenia starting May 18. Armenpress. May 14, 2020. Available from: <https://armenpress.am/eng/news/1015331/> Accessed July 6, 2020.

**Australia**.

S15. No author listed. Victorian coronavirus cases climb as Government makes masks mandatory for Melbourne and Mitchell Shire. July 18, 2020. Available from: <https://www.abc.net.au/news/2020-07-19/victoria-coronavirus-cases-climb-masks-become-mandatory/12470424> Accessed August 8, 2020.

**Austria**.

S16. ORF. Regierung verschärft Maßnahmen. March 30, 2020. Available from: <https://orf.at/stories/3159909/> Accessed May 15, 2020.

S17. No author listed. Austria widening face-mask requirement while loosening lockdown. Reuters. April 6, 2020. Available from: <https://www.reuters.com/article/health-coronavirus-austria-masks/austria-widening-face-mask-requirement-while-loosening-lockdown-idUSV9N28601G> Accessed May 15, 2020.

**Azerbaijan**.

S18. No author listed. People not using medical masks to be penalized: Raids launched. AZTV. May 8, 2020. Available from: <http://www.aztv.az/en/news/6905/people-not-using-medical-masks-to-be-penalized-raids-launched> Accessed July 6, 2020.

**Bahamas**.

S19. No author listed. Bahamas PM’s National Press Conference: Update on COVID-19 Response – April 19th, 2020. Eleuthera News. Available from: <http://eleutheranews.com/?p=22315> Accessed July 6, 2020.

**Bahrain**.

S20. No author listed. Bahrain makes face masks compulsory in public, allows shops to re-open. Arab News. April 9, 2020. Available from: <https://www.arabnews.com/node/1656026/middle-east> Accessed May 30, 2020.

**Bangladesh**.

S21. Paul R. Bangladesh confirms its first three cases of coronavirus. Reuters. March 8, 2020. Available from: <https://www.reuters.com/article/us-health-coronavirus-bangladesh-idUSKBN20V0FS> Accessed August 2, 2020.

S22. No author listed. Coronavirus: Bangladesh confirms first death, 4 new cases. Bangla News. March 18, 2020. Available from: <https://web.archive.org/web/20200326113612/https://www.banglanews24.com/english/national/article/83210/Coronavirus-Bangladesh-confirms-first-death-4-new-cases> Accessed August 2, 2020.

S23. Wadood A, Mamun AS, Rafi A, Islam K, Mohd S, Lee LL, Hossain G. Knowledge, attitude, practice and perception regarding COVID-19 among students in Bangladesh: Survey in Rajshahi University. medRxiv (2020).

S24. Ferdousa MZ, Islama MS, Sikdera MT, Md AS. Knowledge, attitude, and practice regarding COVID-19 outbreak in Bangladesh: An online-based cross-sectional study.

**Barbados**.

S25. Broomes K. Barbados records two cases of COVID-19. Nation News. March 17, 2020. Available from: <https://www.nationnews.com/nationnews/news/244434/barbados-records-covid-19> Accessed August 2, 2020.

S26. No author listed. Shopping Schedule During COVID -19 Curfew. Barbados Government Information Service. April 11, 2020. Available from: <https://gisbarbados.gov.bb/blog/shopping-schedule-during-covid-19-curfew/> Accessed July 8, 2020.

S27. No author listed. COVID-19: Curfew extended for Barbados, but with relaxed restrictions. April 11, 2020. Available from: <https://www.looptt.com/content/covid-19-curfew-extended-barbados-relaxed-restrictions-5> Accessed July 8, 2020.

S28. Charles J, Tavel J, Wyss J, Gamez Torres N. COVID-19 continues to surge in Latin America, Caribbean. Miami Herald. May 1, 2020, as updated May 31, 2020. Available from: <https://web.archive.org/web/20200531181056/https://www.miamiherald.com/news/nation-world/world/americas/haiti/article241249651.html> Accessed July 8, 2020.

S29. No author listed. Face mask required to travel on PSVs. Nation News [Barbados]. May 12, 2020. Available from: <https://www.nationnews.com/nationnews/news/245494/mask-required-travel-psvs> Accessed July 6, 2020.

**Belarus**.

S30. No author listed. Минздрав призывает белорусов соблюдать дистанцирование. Belta. June 5, 2020. Available from: <https://www.belta.by/society/view/minzdrav-prizyvaet-belorusov-sobljudat-distantsirovanie-393694-2020/> Accessed September 26, 2020.

**Belgium.**

S31. Dellanna A. Belgian government pledges free masks for everyone as part of its COVID-19 lockdown exit strategy. Euronews. April 25, 2020. Available from: <https://www.euronews.com/2020/04/25/belgian-government-pledges-free-masks-for-everyone-as-part-of-its-covid-19-lockdown-exit-s> Accessed July 6, 2020.

**Belize**.

S32. No author listed. Belize Makes Face Masks Mandatory in Public. Caribbean Culture and Lifestyle. May 2, 2020. Available from: <https://medium.com/@caribbeanlifestylebelize/belize-makes-face-masks-mandatory-in-public-bd3b3f4f8e88> Accessed July 6, 2020.

**Benin**.

S33. No author listed. More African countries confirm first coronavirus cases as Jack Ma pledges aid. Reuters. March 16, 2020. Available from: <https://www.reuters.com/article/us-health-coronavirus-africa/somalia-liberia-benin-and-tanzania-confirm-first-coronavirus-cases-idUSKBN2131IA> Accessed June 17, 2020.

S34. Coronavirus: Benin recommends use of face mask. Pana Press. April 6, 2020. Available from: <https://www.panapress.com/Coronavirus-Benin-recommends-use-a_630636073-lang2.html> Accessed June 17, 2020.

S35. Agence France Presse. Benin Orders Citizens To Don Anti-virus Masks. April 7, 2020. Available from: <https://www.barrons.com/news/benin-orders-citizens-to-don-anti-virus-masks-01586263504> Accessed June 17, 2020.

S36. Benin Police Enforce Mask Wearing In Bid To Stop Virus. Agence France Presse. April 8, 2020. Available from: <https://www.barrons.com/news/benin-police-enforce-mask-wearing-in-bid-to-stop-virus-01586363106> Accessed May 30, 2020.

**Bhutan**.

S37. No author listed. Bhutan confirms first coronavirus case. Economic Times. March 6, 2020. Avaliable from: <https://economictimes.indiatimes.com/news/international/world-news/bhutan-confirms-first-coronavirus-case/articleshow/74506428.cms> Accessed June 22, 2020.

S38. Ministry of Health, Royal Government of Bhutan. When to use a mask. March 11, 2020. Available from: <https://www.facebook.com/MoHBhutan/posts/when-to-use-maskcoronavirus-covid/2986288851432711/> Accessed June 22, 2020.

**Bolivia**.

S39. Navajas M. Pilares de la “Acción COVID-19”. Ministerio de Salud. May 3, 2020. Available from: <https://www.minsalud.gob.bo/4122-pilares-de-la-accion-covid-19-ministerio-de-salud> Accessed August 8, 2020.

S40. No author listed. COVID-19 Information. U.S. Embassy. May 9, 2020. Available from: <https://web.archive.org/web/20200509084728/https://bo.usembassy.gov/covid-19-information/> Accessed August 8, 2020.

**Bosnia & Herzegovina**.

S41. No author listed. Bosnia confirms its first case of coronavirus. N1 News. March 5, 2020. Available from: <http://ba.n1info.com/English/NEWS/a414110/Bosnia-confirms-its-first-case-of-Coronavirus.html> Accessed June 14, 2020.

S42. No author listed. Bosnia’s Federation entity loosens curfew, introduced mandatory masks. N1 News. March 29, 2020. Available from: <http://ba.n1info.com/English/NEWS/a420560/Bosnia-s-Federation-entity-loosens-curfew-introduced-mandatory-masks.html> Accessed June 10, 2020.

S43. Health Alert: U.S. Embassy Sarajevo, Bosnia and Herzegovina. Available from: <https://ba.usembassy.gov/health-alert-u-s-embassy-sarajevo-bosnia-and-herzegovina-march-30-2020/> Accessed May 6, 2020.

**Botswana**.

S44. No author listed. May 1: Face masks compulsory in public, shared spaces. Africa News [Botswana]. May 1, 2020. Available from: <https://www.africanews.com/2020/07/03/botswana-president-in-self-isolation-after-namibia-trip/> Accessed July 6, 2020.

**Brazil**.

S45. No author listed. Brasil confirma primeiro caso do novo coronavírus. Folha de S. Paulo. February 26, 2020. Available from: <https://www1.folha.uol.com.br/equilibrioesaude/2020/02/brasil-confirma-primeiro-caso-do-novo-coronavirus.shtml> Accessed June 26, 2020.

S46. No author listed. Máscaras caseiras podem ajudar na prevenção contra o Coronavírus. Ministério da Saúde do Brasil. April 2, 2020. Available from: <https://web.archive.org/web/20200403110200/https://www.saude.gov.br/noticias/agencia-saude/46645-mascaras-caseiras-podem-ajudar-na-prevencao-contra-o-coronavirus> Accessed August 8, 2020.

**British Virgin Islands**.

S47. Soverall AJ. BVI residents advised on the best way to wear face masks in public. Ministry of Health & Social Development. April 28, 2020. Available from: <https://bvi.gov.vg/media-centre/bvi-residents-advised-best-way-wear-face-masks-public> Accessed July 7, 2020.

**Brunei**.

S48. Brunei reports more coronavirus cases. The Star. March 10, 2020. Available from: <https://web.archive.org/web/20200312164055/https://www.thestar.com.my/news/regional/2020/03/10/brunei-reports-more-coronavirus-cases> Accessed July 6, 2020.

S49. No author listed. Sultan: Brunei to expand virus testing capacity with new virology lab. The Star. March 22, 2020. Available from: <https://www.thestar.com.my/news/regional/2020/03/22/sultan-brunei-to-expand-virus-testing-capacity-with-new-virology-lab> Accessed July 6, 2020.

**Bulgaria**.

S50. Borissov B. Здравният министър даде заден за задължителните маски. March 31, 2020. Available from:

<https://www.segabg.com/hot/category-bulgaria/gledayte-na-zhivo-vutreshniyat-i-zdravniyat-ministur-razyasnyavat-merkite/> Accessed May 15, 2020.

**Burkina Faso**.

S51. No author listed. COVID-19 Alert: Burkina Faso Updates State of Emergency Measures as of April 20. Worldaware. April 21, 2020. Available from: <https://www.worldaware.com/covid-19-alert-burkina-faso-updates-state-emergency-measures-april-20> Accessed July 6, 2020.

**Burundi**.

S52. No author listed. Burundi's new coronavirus policy: Mass testing in Bujumbura city. July 7, 2020. Aljazeera. Available from: <https://www.aljazeera.com/news/2020/07/burundi-coronavirus-policy-mass-testing-bujumbura-city-200707072204472.html> Accessed August 8, 2020.

**Cabo Verde**.

S53. No author listed. Coronavirus | Cabo Verde – Miscellaneous. ICLG. May 5, 2020. Available from: <https://iclg.com/briefing/12092-coronavirus-cabo-verde-miscellaneous> Accessed July 6, 2020.

**Cambodia**.

S54. Khan S. Cambodia Confirms First Coronavirus Case. VOA Khmer. January 27, 2020. Available from: <https://www.voanews.com/science-health/coronavirus-outbreak/cambodia-confirms-first-coronavirus-case> Accessed May 9, 2020.

S55. Khan S. Cambodian Businesses Embrace Protective Measures Against Coronavirus. VOA Khmer. March 30, 2020. Available from: <https://www.voacambodia.com/a/cambodian-businesses-embrace-protective-measures-against-coronavirus/5352023.html> Accessed May 9, 2020.

S56. Deviller S, Rungjirajittranon M. Fig leaf or first defence? Deploying flimsy masks against virus. Medical Xpress. January 28, 2020. Available from: <https://medicalxpress.com/news/2020-01-fig-leaf-defence-deploying-flimsy.html> Accessed June 6, 2020.

**Cameroon**.^S373^

S57. Unah L, Mussa C. Masks, bans and questions: Inside Cameroon's COVID-19 response. April 23, 2020. Available from: <https://www.aljazeera.com/news/2020/04/masks-bans-questions-cameroon-covid-19-response-200422134140013.html> Accessed May 26, 2020.

S58. No author listed. Cameroon: Authorities announce mandatory face mask use in public places as of April 13. Garda. April 10, 2020. Available from: <https://www.garda.com/crisis24/news-alerts/331246/cameroon-authorities-announce-mandatory-face-mask-use-in-public-places-as-of-april-13-update-4> Accessed July 7, 2020.

S59. Nicholas T, Mandaah FV, Esemu SN, Vanessa AB, Gilchrist KT, Vanessa LF, Shey ND. COVID-19 knowledge, attitudes and practices in a conflict affected area of the South West Region of Cameroon. May-Aug 2020; 36 https://www.panafrican-med-journal.com/content/series/35/2/34/full/ Accessed June 28, 2020.

S60. Davy AA, Victor AD, Valery NN. Socio-Eco-nomic Household Surveys on the Application of Basic Preven-tive Measures Dictated by the WHO to Stop the Spread of COVID-19 in the Northern Zone of Cameroon. Res Rev Infect Dis. 2020;3(1):44-8.

S61. Akwa TE, Muthini MJ, Ning TR. Assessing the Perceptions and Awareness of COVID-19 (Coronavirus) in Cameroon. European Journal of Medical and Educational Technologies. Apr 25, 2020. Available from: https://papers.ssrn.com/sol3/papers.cfm?abstract_id=3628380 Accessed June 29, 2020.

**Canada**.

S62. Chase S. Theresa Tam offers new advice: Wear a non-medical face mask when shopping or using public transit. The Globe and Mail. April 6, 2020. Available from: <https://www.theglobeandmail.com/canada/article-tam-offers-new-advice-wear-a-non-medical-mask-when-shopping-or-using/> Accessed May 7, 2020.

**Cayman Islands**.

S63. No author listed. Wearing Masks & Face Coverings In The Cayman Islands. Cayman Islands Government April 6 2020. Available from: <https://www.exploregov.ky/coronavirus-blog/when-why-to-wear-a-mask> Accessed July 7, 2020.

S64. Young K. Cayman embraces homemade face masks. Cayman Compass. April 1, 2020. Available from: <https://www.caymancompass.com/2020/04/01/cayman-embraces-homemade-face-masks/> Accessed July 7, 2020.

**Central African Republic**.

S65. Kabatanya M. Wearing face masks now compulsory in CAR. UN Missions. June 14, 2020. Available from: <https://minusca.unmissions.org/en/wearing-face-masks-now-compulsory-car> Accessed August 8, 2020.

**Chad**.^S373^

S66. No author listed. Chad confirms first case of coronavirus: government statement. Reuters. March 19, 2020. Available from: <https://www.reuters.com/article/us-health-coronavirus-chad/chad-confirms-first-case-of-coronavirus-government-statement-idUSKBN2162LO> Accessed July 7, 2020.

S67. Presidence de la Republique du Tchad. Communique du Gouvernement. April 13, 2020. Available from: <https://www.presidence.td/fr-news-4198.html> Accessed July 7, 2020.

S68. No author listed. Chad: Authorities announce nationwide COVID-19 restrictions from May 7. Garda. May 11, 2020. Available from: <https://www.garda.com/crisis24/news-alerts/340916/chad-authorities-announce-nationwide-covid-19-restrictions-from-may-7-update-9> Accessed July 7, 2020.

S69. No author listed. Chad Isolates Capital N'Djamena Due To Coronavirus. Barron’s. May 6, 2020. Available from: <https://www.barrons.com/news/chad-isolates-capital-n-djamena-due-to-coronavirus-01588790404> Accessed August 2, 2020.

S70. No author listed. In several countries in central Africa, the mask becomes mandatory. Times Famous. April 15, 2020. Available from: <https://timesfamous.com/worldnews/dans-plusieurs-pays-dafrique-centrale-le-masque-devient-obligatoire/> Accessed July 7, 2020.

**Chile**.

S71. No author listed. Chile afirma estar en una 'guerra' por los recursos contra el COVID-19. Diario Libre. April 6, 2020. Available from: <https://www.diariolibre.com/actualidad/internacional/chile-afirma-estar-en-una-guerra-por-los-recursos-contra-el-covid-19-OJ18118776> Accessed May 7, 2020.

**China**.

S72. Wu H, Yu S. Masks on, Chinese start holiday travels as alarm mounts over mystery virus. Reuters. Jan. 20, 2020. Available from: <https://www.reuters.com/article/us-china-health-pneumonia-masks/masks-on-chinese-start-holiday-travels-as-alarm-mounts-over-mystery-virus-idUSKBN1ZJ0VU> Accessed May 26, 2020.

S73. No author listed. All people in public places in Wuhan required to wear masks, local government says. China Daily. Jan. 22, 2020. Available from: <http://www.chinadaily.com.cn/a/202001/22/WS5e285cada310128217272d84.html> Accessed May 26, 2020.

S74. Epidemic Prevention and Control Group. CDC. Notice regarding the issuance of guidelines for the protection of people with different risks of new coronavirus infection and guidelines for the use of pneumonia masks for the prevention of new coronavirus infection. Jan. 31, 2020. Available from: <http://www.nhc.gov.cn/jkj/s7916/202001/a3a261dabfcf4c3fa365d4eb07ddab34.shtml> Accessed May 26, 2020.

S75. No author listed. Wuhan doctor, colleague of virus whistleblower Li Wenliang, dies from coronavirus. Global Times. March 9, 2020. Available from: <https://www.globaltimes.cn/content/1182009.shtml> Accessed July 31, 2020.

**Colombia**.

S76. No author listed. Which countries have made wearing face masks compulsory? Al Jazeera News. May 5, 2020. Available from: <https://www.aljazeera.com/news/2020/04/countries-wearing-face-masks-compulsory-200423094510867.html> Accessed May 7, 2020.

S77. Hide S. Coronavirus in Colombia: April 3 update. Bogota Post. April 3, 2020. Available from: <https://thebogotapost.com/coronavirus-in-colombia-april-3-update/45493/> Accessed June 24, 2020.

S78. Peckham R. Colombia President Ivan Duque announced April 4 that his administration likely will decide early this week whether and how strict the current national Coronavirus quarantine will extend beyond the presumptive April 13 expiration. Medellin Herald. April 5, 2020. Available from: <https://www.medellinherald.com/ln/item/861-colombia-president-to-define-post-april-13-quarantine-measures-this-week-mask-mandate-now-national> Accessed June10, 2020.

S79. Hide S. Coronavirus in Colombia: April 5 update. Bogota Post. April 5, 2020. Available from: <https://thebogotapost.com/coronavirus-in-colombia-april-5-update/45624/> Accessed June 24, 2020.

**Congo (Brazzaville)**.

S80. Shaban AR. Congo Republic extends coronavirus lockdown. Africa News. May 2, 2020. Available from: <https://www.africanews.com/2020/05/02/congo-republic-extends-coronavirus-lockdown//> Accessed July 6, 2020.

**Costa Rica**.

S81. No author listed. Masks will be mandatory in common areas from Saturday. Paradise Products Costa Rica. June 22, 2020. Available from: <https://www.paradiseproductscr.com/2020/06/22/masks-will-be-mandatory-in-common-areas-from-saturday/> Accessed August 8, 2020.

**Croatia**.

S82. Rogulj D. COVID-19 in Croatia: Recommendations for Public Transportation. Croatia News. April 25, 2020. Available from: <https://www.total-croatia-news.com/travel/43138-croatia> Accessed July 6, 2020.

**Cuba**.

S83. Cuba Suspends International Passenger Flights. Xinhua in Telesur. Available from: <https://www.telesurenglish.net/news/Cuba-Suspends-International-Passenger-Flights-20200402-0012.html> Accessed May 29, 2020.

**Cyprus**.

S84. Agapiou G. Coronavirus: govt adviser says imperative to wear masks in public spaces. Cyprus Mail. April 3, 2020. Available from: <https://cyprus-mail.com/2020/04/03/coronavirus-govt-adviser-says-imperative-to-wear-masks-in-public-spaces/> Accessed May 7, 2020.

**Czechia**.

S85. No author listed. V Česku jsou tři lidé nakažení koronavirem. Předtím byli v Itálii. ČT24. Česka Televize 24. March 1, 2020. Available from: <https://ct24.ceskatelevize.cz/domaci/3056228-v-cesku-jsou-tri-lide-nakazeni-koronavirem> Accessed June 14, 2020.

S86. Veronika B. Could Czech’s Measure to Fight Coronavirus Save Thousands of Lives? Prague Morning. April 4, 2020. Available from: <https://www.praguemorning.cz/could-czechs-measure-to-fight-coronavirus-save-thousands-of-lives-2/> Accessed May 7, 2020.

**Denmark**.

S87. No author listed. Denmark makes masks compulsory on public transport. AFP/TheLocal. August 15, 2020. Available from: <https://www.thelocal.dk/20200815/denmark-makes-masks-compulsory-on-public-transport> Accessed September 26, 2020.

**Democratic Republic of the Congo**.

S88. No author listed. DR Congo Ends Virus Health Emergency, Borders To Reopen. Agence France Presse. July 21, 2020. Available from: <https://www.barrons.com/news/dr-congo-ends-virus-health-emergency-borders-to-reopen-01595399404> Accessed August 8, 2020.

S89. Mbiya BM, Djeugoue SL, Kanda EL, Mbuyi DK, Malundu TB, Mushiya RC, Disashi GT. Coronavirus-19 in the Democratic Republic of Congo: Public Views, Attitudes, and Beliefs in an Unaffected Area: The Case of the City of Mbujimayi. Available from: https://www.preprints.org/manuscript/202006.0317/v1 Accessed June 29, 2020.

**Djibouti**.

S90. No author listed. Republic of Djibouti: COVID-19 Situation Report #7, 10 May 2020. Reliefweb. 11 May 2020. Available from: <https://reliefweb.int/report/djibouti/republic-djibouti-covid-19-situation-report-7-10-may-2020> Accessed July 6, 2020.

**Dominica**.

S91. Baptiste D. Dominica records first case of coronavirus. Loop Jamaica. March 22, 2020. Available from: <http://www.loopjamaica.com/content/watch-dominica-records-first-case-coronavirus-3> Accessed July 6, 2020.

S92. No author listed. COVID-19: Dominica’s president welcomes bi-partisan support for state of emergency and curfew extension. Dominica News Online. April 9, 2020. Available from: <https://dominicanewsonline.com/news/homepage/homepage-carousel/covid-19-dominicas-president-welcomes-bi-partisan-support-for-state-of-emergency-and-curfew-extension/> Accessed July 6, 2020.

S93. No author listed. COVID-19 is a global war says Dominican PM Dr Skerrit on DBS. Wic News. March 31, 2020. Available from: <https://wicnews.com/caribbean/dominica/covid-19-global-war-says-dominican-pm-dr-skerrit-dbs-291326849/> Accessed July 6, 2020.

S94. King A. Guidance for wearing, removing, and discarding protective masks. GIS Dominica. April 7, 2020. Available from: <https://m.facebook.com/watch/?v=867718350318637&_rdr> Accessed July 6, 2020.

S95. No author listed. 3 Covid cases left in Dominica. Dominica Daily. April 25, 2020. Available from: <https://www.thedominicadaily.com/2020/04/3-covid-cases-left-in-dominica.html> Accessed July 6, 2020.

S96. Dominica has done well in preventing the spread of COVID-19 – Dr. Sam Christian. Dominica News Online. April 21, 2020. Available from: <https://dominicanewsonline.com/news/homepage/homepage-carousel/sam-christian-says-dominica-has-done-well-in-preventing-the-spread-of-covid-19/> Accessed July 6, 2020.

S97. No author listed. Dominica: International organizations caution against stigmatization of people affected by COVID-19. International Organization for Migration. April 20, 2020. Available from:

<https://www.programamesoamerica.iom.int/en/news/dominica-international-organizations-caution-against-stigmatization-people-affected-covid-19> Accessed July 7, 2020.

S98. No author listed. Nine recover from COVID-19 in Dominica. Dominica News Online. April 22, 2020. Available from: <https://dominicanewsonline.com/news/homepage/news/nine-recover-from-covid-19-in-dominica/> Accessed July 7, 2020.

**Dominican Republic**.

S99. No author listed. In the absence of medical face masks, headscarves, recommends Minister of Public Health. Dominican Today. April 6, 2020. Available from: <https://dominicantoday.com/dr/local/2020/04/06/in-the-absence-of-medical-face-masks-headscarves-recommends-minister-of-public-health/> Accessed July 7, 2020.

S100. No author listed. El uso de mascarillas en el país será obligatorio en espacios públicos y lugares de trabajo. Listin Diario. April 16, 2020. Available from: <https://listindiario.com/economia/2020/04/16/613491/el-uso-de-mascarillas-en-el-pais-sera-obligatorio-en-espacios-publicos-y-lugares-de-trabajo> Accessed May 7, 2020.

**Ecuador**.

S101. No author listed. Ministerio de Salud confirma primer caso de coronavirus en Ecuador. El Comercio. February 29, 2020. Available from: <https://www.elcomercio.com/actualidad/salud-confirma-primer-caso-coronavirus.html> Accessed June 27, 2020.

S102. No author listed. Ecuador confirma primera muerte por coronavirus. Reuters. March 13, 2020. Available from: <https://www.infobae.com/america/agencias/2020/03/13/ecuador-confirma-primera-muerte-por-coronavirus/> Accessed June 27, 2020.

S103. Armus T. Bodies of coronavirus victims are left on the streets in Ecuador's largest city. San Antonio News Express. April 3, 2020. Available from: <https://www.expressnews.com/news/article/Bodies-of-coronavirus-victims-are-left-on-the-15176423.php> Accessed June 24, 2020.

S104. No author listed. Face masks now required in public; New virus cases trend down; Prostitution ruled non-essential; Ecuador, Peru target border crossings. Cuenca High Life. April 7, 2020. Available from: <https://cuencahighlife.com/face-masks-now-required-in-public-new-virus-cases-trend-down-prostitution-ruled-non-essential-ecuador-peru-target-border-crossings/> Accessed June 24, 2020.

**Egypt**.

S105. Fouly M. Feature: Young Egyptians launch campaign to provide face masks, raise coronavirus awareness in streets. Xinhua. March 20, 2020. Available from: <http://www.xinhuanet.com/english/2020-03/21/c_138902950.htm> Accessed June 19, 2020.

S106. Morsi A. Masks in public places: ‘Even a piece of cloth will do’. Ahram Online. June 2, 2020. Available from: <http://english.ahram.org.eg/NewsContent/50/1201/370399/AlAhram-Weekly/Egypt/Masks-in-public-places-%E2%80%98Even-a-piece-of-cloth-will.aspx> Accessed July 7, 2020.

S107. Samir Abdelhafiz A, Mohammed Z, Ibrahim ME, Ziady HH, Alorabi M, Ayyad M, Sultan EA. Knowledge, perceptions, and attitudes of Egyptians towards the novel coronavirus disease (COVID-19). Journal of Community Health. April 21, 2020. Available from: https://link.springer.com/content/pdf/10.1007/s10900-020-00827-7.pdf Accessed June 19, 2020.

**El Salvador**.

S108. Gómez R. Primer caso de COVID-19 en El Salvador pudo haber entrado por punto ciego en Metapán. La Prensa Grafica. March 18, 2020. Available from: <https://www.laprensagrafica.com/elsalvador/Primer-caso-de-COVID-19-en-El-Salvador-pudo-haber-entrado-por-punto-ciego-en-Metapan-se-ha-activado-cerco-sanitario-por-48-horas-en-ese-municipio-20200318-0064.html>

S109. Corchado A. El Salvador duplicará pruebas para detectar coronavirus. AS. April 4, 2020. Available from: <https://us.as.com/us/2020/04/05/tikitakas/1586044839_590985.html> Accessed July 8, 2020.

S110. Delcid M. Disponen el uso obligatorio de mascarillas en San Salvador para prevenir coronavirus. CNN Espanol. April 8, 2020. Available from: <https://cnnespanol.cnn.com/2020/04/08/disponen-el-uso-obligatorio-de-mascarillas-en-san-salvador-para-prevenir-coronavirus/> Accessed July 8, 2020.

S111. No author listed. Coronavirus.- El Salvador obliga al uso de mascarillas fuera de las viviendas por el coronavirus. Notiamerica. April 12, 2020. Available from: <https://www.notimerica.com/sociedad/noticia-coronavirus-salvador-obliga-uso-mascarillas-fuera-viviendas-coronavirus-20200412072353.html> Accessed July 8, 2020.

**Equatorial Guinea**.

S112. Health Alert: Equatorial Guinea, Government Extends COVID-19 Containment Measures. Overseas Security Advisory Council. April 15, 2020. Available from: <https://www.osac.gov/Country/EquatorialGuinea/Content/Detail/Report/f079c408-dbc7-421c-b6b1-1873c40040dd> Accessed May 7, 2020.

**Estonia**.

S113. No author listed. Prime minister: We are unfortunately still in coronavirus deepening phase. ERR News. April 5, 2020. Available from: <https://news.err.ee/1073236/prime-minister-we-are-unfortunately-still-in-coronavirus-deepening-phase> Accessed June 24, 2020.

**Eswatini**.

S114. No author listed. Coronavirus – Eswatini: COVID-19 update, 30 May 2020. CNBC. May 31, 2020. Available from: <https://www.cnbcafrica.com/africa-press-office/2020/05/31/coronavirus-eswatini-covid-19-update-30-may-2020/> Accessed August 8, 2020.

**Ethiopia**.

S115. Samuel G. Ethiopia Outlaws Handshakes, Obliges Masks in Public Places. Addis Fortune. April 12, 2020.  Available from: <https://addisfortune.news/ethiopia-outlaws-handshakes-obliges-masks-in-public-places/> Accessed May 30, 2020.

S116. Akalu Y, Ayelign B, Molla MD. Knowledge, Attitude and Practice Towards COVID-19 Among Chronic Disease Patients at Addis Zemen Hospital, Northwest Ethiopia. Infection and Drug Resistance. 2020 Jun 24;13:1949-60.

S117. Kebede Y, Yitayih Y, Birhanu Z, Mekonen S, Ambelu A. Knowledge, perceptions and preventive practices towards COVID-19 among Jimma University Medical Center visitors, Southwest Ethiopia. Researchsquare. doi: 10.21203/rs.3.rs-25865/v1

S118. Kassaw C. The Psychological Impact of COVID-19 Pandemic among Communities Living in Dilla Town, Ethiopia, April 2020. June 30, 2020. Available from: https://www.preprints.org/manuscript/202006.0356/v1 Accessed July 31, 2020.

S119. Bekele D, Tolossa T, Tsegaye R, Teshome W. The knowledge and practice towards COVID-19 pandemic prevention among residents of Ethiopia. An online cross-sectional study. BioRxiv. 2020 Jan 1.

**Finland**.

S120. Finland encourages use of face masks in policy turnaround. Medical Press. April 14, 2020. Available from: <https://medicalxpress.com/news/2020-04-finland-masks-policy-turnaround.html> Accessed May 7, 2020

**France**.

S121. Coronavirus en France : le parcours des trois patients. Franceinfo. January 25, 2020. Available from: <https://www.francetvinfo.fr/sante/maladie/coronavirus/coronavirus-en-france-le-parcours-des-trois-patients_3799837.html> Accessed June 20, 2020.

S122. No author listed. Coronavirus: First death confirmed in Europe. BBC News. February 15, 2020. Available from: <https://www.bbc.com/news/world-europe-51514837> Accessed June 20, 2020.

S123. Coronavirus: U-turn on face masks in France and the United States. France24. April 4, 2020. Available from: <https://www.france24.com/en/20200404-new-york-is-in-a-race-against-time-against-virus-as-trump-says-masks-are-voluntary> Accessed June 20, 2020.

**French Guayana**.^S370^

**Gabon**.^S372,S373^

**Gambia**.

S124. Gambia: COVID-19 and the mad rush for face masks! African Press Agency News. March 18, 2020. Available from: http://apanews.net/mobile/uneInterieure_EN.php?id=4937708 Accessed May 7, 2020.

S125. Gambia Ministry of Health. The Gambia COVID-19 Outbreak Situational Report. May 26, 2020. Available from: <http://www.moh.gov.gm/wp-content/uploads/2020/05/Gambia_The_COVID-19_Sitreps-26-05-2020.pdf> Accessed June 10, 2020.

S126. No author listed. Gambia: Government Spokesperson - Masks Wearing Is Compulsory in the Gambia. Allafrica. July 18, 2020. Available from: <https://allafrica.com/stories/202007200236.html> Accessed August 8, 2020.

**Georgia**.

S127. U.S. Embassy in Georgia. Alert: Prohibition on car traffic starting 12 noon Friday, April 17. April 17, 2020. Available from: <https://ge.usembassy.gov/alert-prohibition-on-car-traffic-starting-12-noon-friday-april-17/> Accessed July 7, 2020.

**Germany**.

S128. No author listed. Bayerische Behörden bestätigen ersten Fall in Deutschland. Der Spiegel. January 28, 2020. Available from: <https://www.spiegel.de/wissenschaft/medizin/corona-virus-erster-fall-in-deutschland-bestaetigt-a-19843b8d-8694-451f-baf7-0189d3356f99> Accessed June 20, 2020.

S129. Chambers M. German city introduces face masks for shoppers as coronavirus spreads. Reuters. March 31, 2020. Available from: <https://www.reuters.com/article/us-health-coronavirus-germany-masks/german-city-introduces-face-masks-for-shoppers-as-coronavirus-spreads-idUSKBN21I10K> Accessed May 7, 2020.

S130. No author listed. ‘They could reduce the risk': Germany's public health institute updates stance on face masks. The Local de. April 2, 2020. Available from: <https://www.thelocal.de/20200402/latest-face-masks-in-public-could-help-to-reduce-spread-of-coronavirus-says-germanys-robert-koch-institute> Accessed June 20, 2020.

S131. No author listed. Coronavirus: Germany's states make face masks compulsory. BBC News. April 22, 2020. Available from: <https://www.bbc.com/news/world-europe-52382196> Accessed July 7, 2020.

**Ghana**.

S132. No author listed. Every Ghanaian must wear masks. ETV Ghana. April 20, 2020. Available from: <https://www.etvghana.com/every-ghanaian-must-wear-masks-nana-addo/> Accessed July 6, 2020.

S133. Ghana Health Service. Ministerial directive on wearing masks in public places to prevent transmission of COVID-19. Ghana Health Service. April 25, 2020. Available from: <https://ghanahealthservice.org/covid19/downloads/covid_19_nose_mask.pdf> Accessed June 29, 2020.

S134. Bonful H, Addo-Lartey A, Aheto J, Sarfo B, Aryeetey R. Limiting Spread of COVID-19 in Ghana: Compliance audit of selected transportation stations in the Greater Accra region of Ghana. medRxiv. 2020.

**Greece**.

S135. No author listed. Στους 136 οι νεκροί – Υποχρεωτική η χρήση μάσκας σε ΜΜΜ και κλειστούς χώρους. The Press Project. April 27, 2020. Available from: <https://thepressproject.gr/stous-136-i-nekri-ypochreotiki-i-chrisi-maskas-se-mmm-ke-klistous-chorous/> Accessed July 7, 2020.

**Grenada**.

S136. Wong M. Grenada records first COVID-19 case. loopnewsbarbados. March 22, 2020. Available from: <http://www.loopnewsbarbados.com/content/grenada-records-first-covid-19-case-4> Accessed June 17, 2020.

S137. Ministry of Health Grenada. Be COVID-19 smart—wear a mask. April 3, 2020. Available from: <https://www.facebook.com/HealthGrenada/posts/be-covid19-smart-wear-a-maskmasks-are-effective-in-helping-to-slow-the-spread-of/738937303305031/> Accessed June 17, 2020.

S138. Cabinet of Grenada. Emergency Powers (Covid-19) (No. 3) Regulations, 2020. April 6, 2020. Available from: <https://www.nowgrenada.com/2020/04/emergency-powers-covid-19-no-3-regulations-2020/> Accessed June 17, 2020.

**Guadeloupe**.^S370^

**Guatemala**.

S139. Health Alert: Guatemala, Government Mandates Wearing of Masks in Public Spaces. Oversea Security Advisory Council. April 9, 2020. Available from: <https://www.osac.gov/Country/Guatemala/Content/Detail/Report/01cbc8c3-6795-4fc8-a941-1867c560f0df> Accessed May 7, 2020.

**Guinea**.

S140. Conde A. #COVID19 Le port de masque communautaire ou bavette est maintenant OBLIGATOIRE pour tout citoyen à compter du samedi 18 avril 2020. Twitter. April 13, 2020. Available from: <https://twitter.com/alphacondepresi/status/1249793942368980996> Accessed July 18, 2020.

**Guinea-Bissau**.

S141. No author listed. Guinea-Bissau's president extends state of emergency untill May 26. Xinhua. May 11, 2020. Available from: <http://www.xinhuanet.com/english/2020-05/12/c_139048499.htm> Accessed July 7, 2020.

**Guyana**.

S142. Charles J, Tavel J, Wyss J, Gamez Torres N. Latin America and Caribbean yet to hit coronavirus surge; most are tightening measures. Miami Herald. April 17, 2020. Available from: <https://web.archive.org/web/20200418093401/https://www.miamiherald.com/news/nation-world/world/americas/haiti/article241249651.html> Accessed July 7, 2020.

**Haiti**.

S143. Charles J. Prime minister orders Haitians to wear masks in public and orders firms to make more masks. Miami Herald. May 5, 2020. Available from: <https://www.miamiherald.com/news/nation-world/world/americas/haiti/article242507476.html> Accessed July 8, 2020.

S144. Charles J, Tavel J, Wyss J, Gamez Torres N. As region eases restrictions on fighting coronavirus, PAHO expresses concerns. Miami Herald. May 01, 2020 [May 7 update] Available from: <https://web.archive.org/web/20200516122255/https://www.miamiherald.com/news/nation-world/world/americas/haiti/article241249651.html> Accessed July 8, 2020.

**Honduras**.

S145. Archeta K. Honduras makes it mandatory to wear face masks in public. COVID-19 World News. April 7, 2020. Available from: <https://covid19data.com/2020/04/07/honduras-makes-it-mandatory-to-wear-face-masks-in-public/> Accessed May 7, 2020.

**Hong Kong**.

S146. Department of Health, Hong Kong. Latest recommendations by Scientific Committee on Emerging and Zoonotic Diseases and Scientific Committee on Infection Control after reviewing cases of novel coronavirus infection. January 24, 2020. Available from: <https://www.info.gov.hk/gia/general/202001/24/P2020012400762.htm> Accessed May 9, 2020.

S147. Cheung E. China coronavirus: death toll almost doubles in one day as Hong Kong reports its first two cases. South China Morning Post. January 22, 2020. Available from: <https://www.scmp.com/news/hong-kong/health-environment/article/3047193/china-coronavirus-first-case-confirmed-hong-kong> Accessed May 9, 2020.

S148. Wu P, Tsang TK, Wong JY, et al. Suppressing COVID-19 transmission in Hong Kong: an observational study of the first four months. Available from: <https://assets.researchsquare.com/files/rs-34047/v1/e26fcc0f-8101-4007-9c2a-6fb1b24bc8ea.pdf> Accessed June 19, 2020.

S149. Tam VC, Tam SY, Poon WK, Law HK, Lee SW. A reality check on the use of face masks during the COVID-19 outbreak in Hong Kong. Lancet. 2020 May 1;22.

**Hungary**.

S150. Szakacs G. Hungary eases coronavirus restrictions outside Budapest. National Post. April 29, 2020. Available from: <https://nationalpost.com/pmn/health-pmn/hungary-eases-coronavirus-restrictions-outside-budapest-pm> Accessed July 8, 2020.

**Iceland**.

S151. Hafstað V. No author listed. New Restrictions Take Effect in Iceland Tomorrow. Iceland Monitor. July 30, 2020. Available from: <https://icelandmonitor.mbl.is/news/politics_and_society/2020/07/30/new_restrictions_take_effect_in_iceland_tomorrow/> Accessed September 26, 2020.

**India**.

S152. Reid D. India confirms its first coronavirus case. CNBC. January 30, 2020. Available from: <https://www.cnbc.com/2020/01/30/india-confirms-first-case-of-the-coronavirus.html> Accessed June 19, 2020.

S153. Thacker T. Amid outbreak, Health Ministry recommends homemade face masks. Economic times. Apr 4, 2020. Available from:

<https://economictimes.indiatimes.com/industry/healthcare/biotech/healthcare/amid-outbreak-health-ministry-recommends-homemade-face-masks/articleshow/74978175.cms> Accessed May 29, 2020.

S154. Gudi SK, Undela K, Venkataraman R, Mateti UV, Chhabra M, Nyamagoud S, Tiwari KK. Knowledge and Beliefs towards Universal Safety Precautions to flatten the curve during Novel Coronavirus Disease (nCOVID-19) Pandemic among general Public in India: Explorations from a National Perspective. Available from: https://www.medrxiv.org/content/10.1101/2020.03.31.20047126v1 Accessed July 31, 2020.

**Indonesia**.

S155. Soeriaatmadja W. Coronavirus: Price of a box of N95 masks cost more than a gram of gold in Indonesia. Straits Times. February 10, 2020. Available from: <https://www.straitstimes.com/asia/se-asia/coronavirus-price-of-a-box-n95-masks-cost-more-than-a-gram-of-gold-in-indonesia>

S156. No author listed. Indonesia confirms first cases of coronavirus. Bangkok Post. March 2, 2020. Available from: <https://www.bangkokpost.com/world/1869789/indonesia-confirms-first-cases-of-coronavirus> Accessed June 19, 2020.

S157. Afriyadi AD. Telkom Buka Suara Ada Karyawannya Meninggal Positif Corona. Detik Finance. March 16, 2020. Available from: <https://finance.detik.com/berita-ekonomi-bisnis/d-4940364/telkom-buka-suara-ada-karyawannya-meninggal-positif-corona> Accessed June 19, 2020.

S158. Yulisman L. Coronavirus: Indonesia makes face masks compulsory as death toll nears 200. Straits Times. April 5, 2020. Available from:

<https://www.straitstimes.com/asia/se-asia/coronavirus-indonesia-orders-citizens-to-wear-masks-as-infections-rise> Accessed May 29, 2020.

S159. Ardan M, Rahman FF, Geroda GB. The influence of physical distance to student anxiety on COVID-19, Indonesia. Journal of Critical Reviews. 2020;7(17):1126-32.

**Iran**.

S160. No author listed. Two Iranians die after testing positive for coronavirus. Reuters. February 19, 2020. Available from: <https://www.cnbc.com/2020/02/19/two-iranians-die-after-testing-positive-for-coronavirus.html> Accessed June 27, 2020.

S161. Borger J. Satellite images show Iran has built mass graves amid coronavirus outbreak. The Guardian. March 12, 2020. Available from: <https://www.theguardian.com/world/2020/mar/12/coronavirus-iran-mass-graves-qom> Accessed June 27, 2020.

S162. Wright R. How Iran became a new epicenter of the coronavirus outbreak. The New Yorker. 2020 Feb 28.

S163. Kakemam E, Ghoddoosi-Nejad D, Chegini Z, Momeni K, Salehinia H, Hassanipour S, Ameri H, Arab-Zozani M. Knowledge, attitudes, and practices among the general population during COVID-19 outbreak in Iran: A national cross-sectional survey. medRxiv. 2020.

S164. Tuite AR, Bogoch II, Sherbo R, Watts A, Fisman D, Khan K. Estimation of coronavirus disease 2019 (COVID-19) burden and potential for international dissemination of infection from Iran. Annals of Internal Medicine. 2020 May 19;172(10):699-701.

S165. Zhuang Z, Zhao S, Lin Q, Cao P, Lou Y, Yang L, He D. Preliminary estimation of the novel coronavirus disease (COVID-19) cases in Iran: A modelling analysis based on overseas cases and air travel data. International Journal of Infectious Diseases. 2020 May 1;94:29-31.

**Iraq**.

S166. No author listed. Covid-19: Iraq announces changes to curfew, other restrictions. Government of Iraq. April 20, 2020. Available from: <https://gds.gov.iq/covid-19-iraq-announces-changes-to-curfew-other-restrictions/> Accessed July 8, 2020.

**Ireland**.

S167. No author listed. Taoiseach advises public to wear face coverings on public transport and in retail stores. JournalIE. May 15, 2020. Available from: <https://www.thejournal.ie/face-coverings-on-public-transport-and-in-retail-outlets-5098881-May2020/> Accessed July 8, 2020.

**Israel**.

S168. Staff T. Netanyahu urges wearing masks outside; announces stipends for kids, elderly. The Times of Israel. April 1, 2020. Available from: <https://www.timesofisrael.com/netanyahu-tells-israelis-to-wear-masks-outside-gives-stipends-for-kids-elderly> Accessed May 8, 2020.

**Italy**.

S169. Severgnini C. Coronavirus, primi due casi in Italia «Sono due cinesi in vacanza a Roma» Sono arrivati a Milano il 23 gennaio. Corriere Della Sera. January 31, 2020. Available from: <https://www.corriere.it/cronache/20_gennaio_30/coronavirus-italia-corona-9d6dc436-4343-11ea-bdc8-faf1f56f19b7.shtml?refresh_ce-cp> Accessed June 20, 2020.

S170. Godin M. Why Is Italy's Coronavirus Outbreak So Bad? Time. March 10, 2020. Available from: <https://time.com/5799586/italy-coronavirus-outbreak/> Accessed June 20, 2020.

S171. No author listed. Coronavirus: Lombardy, Tuscany make face masks compulsory. April 6, 2020. ANSA. Available from: <https://www.ansa.it/english/news/2020/04/06/coronavirus-lombardy-makes-face-masks-compulsory_a852ffdb-a0dd-4c55-a725-e852c5a2fc43.html> Accessed June 20, 2020.

S172. No author listed. UPDATE: When and where do you need to wear a face mask in Italy? The Local IT. April 28, 2020. Available from: <https://www.thelocal.it/20200428/coronavirus-where-should-you-wear-a-face-mask-in-italy> Accessed July 8, 2020.

**Ivory Coast**.^S66^

S173. No author listed. Ivory Coast confirms first case of coronavirus. Reuters. Daily Sabah. March 11, 2020. Available from: <https://www.dailysabah.com/world/ivory-coast-confirms-first-case-of-coronavirus/news> Accessed June 13, 2020.

S174. No author listed. Coronavirus: Ivory Coast protesters target testing centre. BBC News. April 6, 2020. Available from: <https://www.bbc.com/news/world-africa-52189144> Accessed June 18, 2020.

**Jamaica**.

S175. Mundle T. PM Announces Order For Wearing Of Masks. Jamaica Information Service. April 9, 2020. Available from: <https://jis.gov.jm/pm-announces-order-for-wearing-of-masks/> Accessed July 12, 2020.

S176. Mundle T. All Jamaicans Must Wear A Mask In Public – PM. Jamaica Information Service. April 21, 2020. Available from: <https://jis.gov.jm/all-jamaicans-must-wear-a-mask-in-public-pm/> Accessed July 8, 2020.

**Japan**.

S177. Takahashi. Amid virus outbreak, Japan stores scramble to meet demand for face masks. Japan Times. Available from: <https://www.japantimes.co.jp/news/2020/01/31/national/coronavirus-japan-surgical-masks/#.Xrc8Z2hKhPY> Accessed May 9, 2020.

S178. Kawai Y. New China virus spurs 24-hour output of surgical masks in Japan. Nikkei Asian Review. January 18, 2020. Available from:

<https://asia.nikkei.com/Business/Business-trends/New-China-virus-spurs-24-hour-output-of-surgical-masks-in-Japan> Accessed June 6, 2020.

S179. No author listed. Face masks and hand sanitizers in short supply across Asia amid deadly virus panic. Japan Times. January 24, 2020. Available from: <https://www.japantimes.co.jp/news/2020/01/24/business/face-masks-hand-sanitizers-asia-coronavirus/#.Xtv0JWhKhPY> Accessed June 6, 2020.

**Jordan**.

S180. No author listed. King meets with governors, urges alleviating burden on citizens by monitoring prices. Jordan Times. April 27, 2020. Available from: <https://www.jordantimes.com/news/local/king-meets-governors-urges-alleviating-burden-citizens-monitoring-prices> Accessed July 8, 2020.

S181. No author listed. Epidemiological situation in Jordan is stable. Jordan Times. April 29, 2020. Available from: <https://menafn.com/1100093174/Epidemiological-situation-in-Jordan-is-stable> Accessed July 8, 2020.

S182. Olaimat AN, Aolymat I, Elsahoryi N, Shahbaz HM, Holley RA. Attitudes, Anxiety, and Behavioral Practices Regarding COVID-19 among University Students in Jordan: A Cross-Sectional Study. Am J Trop Med Hygiene. 2020 July 8, 2020. Available from: <http://www.ajtmh.org/docserver/fulltext/10.4269/ajtmh.20-0418/tpmd200418.pdf?expires=1596199449&id=id&accname=guest&checksum=37D8DD443CB1475F07A83B5F6BEA76DC> Accessed July 31, 2020.

**Kazakhstan**.

S183. No author listed. Wearing face masks in public places obligatory – President. Kazinform. May 26, 2020. Available from: <https://www.inform.kz/en/wearing-face-masks-in-public-places-obligatory-president_a3654072> Accessed July 8, 2020.

**Kenya**.

S184. Ministry of Health, Republic of Kenya. First case of coronavirus disease confirmed in Kenya. March 13, 2020. Available from: <https://www.health.go.ke/first-case-of-coronavirus-disease-confirmed-in-kenya/> Accessed June 20, 2020.

S185. Munde C. First Kenyan dies of Covid-19 - CS Kagwe. The Star. March 26, 2020. Available from: <https://www.the-star.co.ke/covid-19/2020-03-26-first-kenyan-dies-of-covid-19-cs-kagwe/> Accessed June 20, 2020.

S186. Muraya J. Masks Are Not Optional, Kagwe Tells Passengers As Virus Threat Heightened. Capitol News. April 3, 2020. Available from: <https://www.capitalfm.co.ke/news/2020/04/masks-are-not-optional-kagwe-tells-passengers-as-virus-threat-heightened/> Accessed June 20, 2020.

S187. Muraya J. Kenya: Masks Now Mandatory in Public Places, Kenya Declares. All Africa. April 5, 2020. Available from: <https://allafrica.com/stories/202004060049.html> Accessed May 29, 2020.

S188. Austrian K, Abuya T. We wanted to know how coronavirus affects Nairobi’s slum residents. What we found. The Conversation. May 5, 2020. Available from: <https://theconversation.com/we-wanted-to-know-how-coronavirus-affects-nairobis-slum-residents-what-we-found-137621> Accessed June 20, 2020.

**Kuwait**^14^

**Kyrgyzstan**.

S189. No author listed. COVID-19 alert: Kyrgyzstan lifts state of emergency in major urban areas, maintains several restrictions as of May 11. WorldAware. May 11, 2020. Available from: <https://www.worldaware.com/covid-19-alert-kyrgyzstan-lifts-state-emergency-major-urban-areas-maintains-several-restrictions> Accessed July 9, 2020.

**Laos**.

S190. Uy MH. Lao morning news for March 6. AEC News. March 6, 2020. Available from: <https://aecnewstoday.com/2020/lao-morning-news-for-march-6-3/> Accessed June 6, 2020.

S191. No author listed. Laos reports no case of COVID-19, to import face masks from Vietnam. Vietnam Times. March 20, 2020. Available from: <https://vietnamtimes.org.vn/laos-reports-no-case-of-covid-19-to-import-face-masks-from-vietnam-18566.html> Accessed May 9, 2020.

**Latvia**.

S192. No author listed. Latvian PM urges everyone to wear face masks in public places. Latvian Public Broadcasting. April 28, 2020. Available from: <https://eng.lsm.lv/article/society/health/latvian-pm-urges-everyone-to-wear-face-masks-in-public-places.a357722/> Accessed July 9, 2020.

**Lebanon**.

S193. Sly L. Lebanon is in a big mess. But on coronavirus, it’s doing something right. Washington Post. April 22, 2020. Available from: <https://www.washingtonpost.com/world/middle_east/lebanon-is-in-a-big-mess-but-on-coronavirus-its-doing-something-right/2020/04/21/a024496a-83e0-11ea-81a3-9690c9881111_story.html> Accessed June 24, 2020.

S194. No author listed. Lebanon divided over face masks in virus battle. Arab News. April 5, 2020. Available from: <https://www.arabnews.com/node/1653251/middle-east> Accessed May 8, 2020.

S195. Houssari N. Lebanese must wear face masks despite coronavirus lockdown transition period. Arab News. April 25, 2020. Available from: <https://arab.news/nzbdk> Accessed June 24, 2020.

S196. Houssari N. Lebanon issues fines to enforce wearing of face masks. Arab News. May 9, 2020. Available from: <https://www.arabnews.com/node/1681831/middle-east> Accessed August 2, 2020.

**Liberia**.

S197. No author listed. Pres. Weah Extends "STAY HOME" Order Under State of Emergency. Executive Mansion [Liberia]. April 24, 2020. Available from: <https://www.emansion.gov.lr/2press.php?news_id=5144&related=7&pg=sp> Accessed July 9, 2020.

**Libya**.

S198. Karabacak S. Libya to impose 10-day curfew to combat COVID-19. Anadolu Agency. April 16, 2020. Available from: <https://www.aa.com.tr/en/africa/libya-to-impose-10-day-curfew-to-combat-covid-19/1806641> Accessed May 8, 2020.

**Liechtenstein**.

S199. No author listed. Measures Taken in Liechtenstein in Response to the Coronavirus Pandemic. Embassy of the Principality of Liechtenstein. July 3, 2020. Available from: <http://www.liechtensteinusa.org/article/measures-taken-in-liechtenstein-in-response-to-the-coronavirus-pandemic> Accessed July 9, 2020.

**Lithuania**.

S200. No author listed. Lithuania to keep quarantine in place until April 13. Baltic News Network. March 26, 2020. Available from: <https://bnn-news.com/lithuania-to-keep-quarantine-in-place-until-april-13-211764> Accessed June 24, 2020.

S201. Jačauskas I. Lithuanian government extends quarantine, makes facemasks mandatory. LRT English. April 8, 2020. Available from: <https://www.lrt.lt/en/news-in-english/19/1161456/lithuanian-government-extends-quarantine-makes-facemasks-mandatory> Accessed May 8, 2020.

**Luxembourg**.

S202. Oglesby K. Construction back in action, masks mandatory from Monday. Luxembourg Times. April 15, 2020. Available from: <https://luxtimes.lu/luxembourg/40429-construction-back-in-action-masks-mandatory-from-monday> Accessed July 9, 2020.

**Macao**.

S203. Chung K. Macau confirms second patient infected with Chinese coronavirus. South China Morning Post. Available from:

<https://www.scmp.com/news/china/article/3047337/macau-confirms-second-patient-infected-chinese-coronavirus> Accessed May 26, 2020.

**Madagascar**.

S204. No author listed. Madagascar: COVID-19 lockdown measures begin to ease in major cities April 20. Garda World. April 21, 2020. Available from: <https://www.garda.com/crisis24/news-alerts/334481/madagascar-covid-19-lockdown-measures-begin-to-ease-in-major-cities-april-20-update-6> Accessed July 9, 2020.

**Malawi**.

S205. No author listed. Just In: Malawi registers first COVID-19 death. Face of Malawi. April 7, 2020. Available from: <https://www.faceofmalawi.com/2020/04/covid-19-first-death-in-malawi/> Accessed June 6, 2020.

S206. Chilunga Z. Malawi: Mutharika Urges Malawi Unity and 'Steadfast' in COVID-19 Fight - Announce New Measures to Stop Spread of Outbreak. Nyasa Times. April 4, 2020. Available from: <https://allafrica.com/stories/202004060182.html> Accessed May 29, 2020.

S207. Banda J, Dube A, Brumfield S, Amoah A, Crampin A, Reniers G, Helleringer S. Knowledge and behaviors related to the COVID-19 pandemic in Malawi. medRxiv. 2020 Jan 1.

**Malaysia**.

S208. Harun HN, Yusof TA, Solhi F. Demand for face masks, hand sanitisers soars. New Straits Times. January 30, 2020. Available from:

<https://www.nst.com.my/news/nation/2020/01/561250/demand-face-masks-hand-sanitisers-soars> Accessed May 9, 2020.

**Maldives**.

S209. Hadi AA. Reopening Maldives: Safe resort licenses, testing tourists for COVID-19. Sun. May 19, 2020. Available from: <https://en.sun.mv/60440> Accessed July 10, 2020.

**Mali**.

S210. No author listed. Health Alert – U.S. Embassy Bamako, Mali – May 11, 2020. Available from: <https://ml.usembassy.gov/health-alert-u-s-embassy-bamako-mali-may-11-2020/> Accessed July 10, 2020.

**Malta**.

S211. No author listed. Masks to be required when shopping or on the bus. Times of Malta. May 1, 2020. Available from: <https://timesofmalta.com/articles/view/masks-to-be-required-when-shopping-or-on-the-bus.789347> Accessed July 10, 2020.

**Mauritania**.

S212. No author listed. Mauritania Eases Pandemic Restrictions. Barrons. May 7, 2020. Available from: <https://www.barrons.com/news/mauritania-eases-pandemic-restrictions-01588842004> Accessed July 10, 2020.

**Mauritius**.

S213. Réouverture des supermarchés et boutiques : voici ce qu'il faut retenir. Le DefiMedia Group. March 31, 2020. Available from: <https://defimedia.info/reouverture-des-supermarches-et-boutiques-voici-ce-quil-faut-retenir> Accessed May 8, 2020.

**Mayotte**.

S214. Département de Mayotte. Covid-19: un masque pour tous les Mahorais. May 11, 2020. Available from: <https://www.facebook.com/conseildepartementalMayotte/posts/1615212958643116/> Accessed July 10, 2020.

**Mexico**.

S215. No author listed. Face masks are not necessarily effective, nor are they convenient. Coronavirus point man repeats reservations over value of face masks. Mexico News Daily. April 28, 2020. Available from: <https://mexiconewsdaily.com/news/coronavirus/coronavirus-point-man-repeats-reservations-over-value-of-face-masks/> Accessed July 10, 2020.

S216. Mexico announces 'new normality' in plan to reopen economy. Aljazeera. May 13, 2020. Available from: <https://www.aljazeera.com/news/2020/05/mexico-announces-normality-plan-reopen-economy-200512231541957.html> Accessed July 10, 2020.

**Moldova**.

S217. No author listed. COVID-19 Information. U.S. Embassy in Moldova. July 9, 2020. Available from: <https://md.usembassy.gov/u-s-citizen-services/covid-19-information/> Accessed July 10, 2020.

**Mongolia**

S218. Baljmaa.T. SEC: MNT 150,000 fine for not wearing face masks. April 14, 2020. Available from: <https://www.montsame.mn/en/read/222259> Accessed May 9, 2020.

S219. Amarsaikhan S. Ulaanbaatar City Mayor's Order A/108. January 27, 2020. Available from: <https://ulaanbaatar.mn/Home/Docdetail?dataID=47003> Accessed August 1, 2020.

S220. Erkhembayar R, Dickinson E, Badarch D, Narula I, Thomas GN, Ochir C, Manaseki-Holland S. Early policy actions and emergency response to the COVID-19 pandemic in Mongolia: experiences and challenges. Lancet Global Health. July 23, 2020. Available from: https://doi.org/10.1016/S2214-109X(20)30295-3 Accessed July 31, 2020.

**Montenegro.**

S221. Stjepčević A. COVID-19 in Montenegro: No New Daily Cases, Update April 30, 2020. Total Montenegro News. April 30, 2020. Available from: <https://www.total-montenegro-news.com/news/5504-covid-19-in-montenegro-no-new-daily-cases-update-april-30-2020> Accessed July 10, 2020.

**Montserrat**.

S222. Farrell JE. COVID 19 Weekly Message by Premier, Hon. Joseph E. Farrell [of Montserrat] – Measures from May 1 -May 7, 2020. April 29, 2020. Available from: <http://www.gov.ms/covid-19-weekly-message-by-premier-hon-joseph-e-farrell-measures-from-may-1-may-7-2020/> Accessed July 10, 2020.

**Morocco**.

S223. Morocco makes face masks compulsory due to coronavirus. Reuters. April 6, 2020. Available from: <https://www.reuters.com/article/us-health-coronavirus-morocco/morocco-makes-face-masks-compulsory-due-to-coronavirus-idUSKBN21O31E> Accessed May 8, 2020.

**Mozambique**.

S224. Mozambique confirms first coronavirus case. National Post. Available from: <https://nationalpost.com/pmn/health-pmn/mozambique-confirms-first-coronavirus-case> Accessed June 6, 2020.

S225. No author listed. Just In: Coronavirus. Mozambique announces first Covid-19 patient recovery. Club of Mozambique. April 4, 2020. Available from: <https://clubofmozambique.com/news/just-in-coronavirus-mozambique-announces-first-covid-19-patient-recovery-156979/> Accessed June 19, 2020.

S226. No author listed. Mozambique: Government Orders Wearing of Masks. allAfrica. April 9, 2020. Available from: <https://allafrica.com/stories/202004091000.html> Accessed May 30, 2020.

**Myanmar**.

S227. Myanmar confirms first two coronavirus cases. Straits Times. March 24, 2020. Available from: <https://www.straitstimes.com/asia/se-asia/myanmar-confirms-first-coronavirus-cases> Accessed June 28, 2020.

S228. Nachemson A. Fears of coronavirus catastrophe as Myanmar reports first death. Al Jazeera. April 1, 2020. Available from: <https://www.aljazeera.com/news/2020/06/500000-dead-coronavirus-live-updates-200628233313992.html> Accessed June 28, 2020.

S229. Dissemination of Preliminary Findings of Public Compliance Survey on Ministry of Health and Sports Guidelines for COVID-19 Prevention. Ministry of Health and Sports (Myanmar). June 3, 2020. Available from: <https://www.mohs.gov.mm/page/10385> Accessed July 14, 2020.

S230. Ministry of Health and Sports (Myanmar). Frequently Asked Questions on Different Types of Mask Use in COVID-19 Prevention and Control. April 5, 2020. Available from: <http://mohs.gov.mm/su/gejx3143GE> Accessed July 14, 2020.

S231. Htet KS. Myanmar State Counsellor makes own face mask. Myanmar Times. April 7, 2020. Available from: <https://www.mmtimes.com/news/myanmar-state-counsellor-makes-own-face-mask.html> Accessed July 15, 2020.

S232. No author listed. Myanmar Steps up Coronavirus Measures as Medical Profession Feels Risks. Radio Free Asia. April 16, 2020. Available from: <https://www.rfa.org/english/news/myanmar/medical-profession-04162020205725.html> Accessed July 10, 2020.

S233. Mya Kyaw S, Aye SM, Hlaing Win A, Hlaing Su S, Thida A. Awareness, perceived risk and protective behaviours of Myanmar adults on COVID-19. International Journal of Community Medicine and Public Health. 2020; 7:1627-36.

**Namibia**.

S234. No author listed. Use of face masks in public becomes mandatory in Namibia. Namibian Broadcasting Corporation. May 2, 2020. Available from: <https://www.nbc.na/news/use-face-masks-public-becomes-mandatory-namibia.30485> Accessed July 10, 2020.

**Nepal**.

S235. No author listed. Coronavirus: Students wear face masks to school in Nepal. Manila Bulletin Online. January 29, 2020. Available from: <https://www.youtube.com/watch?v=gjdykIOjqSU> Accessed June 10, 2020.

S236. Nepalis rush to buy face masks amidst coronavirus outbreak but there are none available. Kathmandu Post. February 3, 2020. Available from: <http://webcache.googleusercontent.com/search?q=cache:BLTdc-faXCkJ:https://kathmandupost.com/national/2020/02/03/nepalis-rush-to-buy-face-masks-amidst-coronavirus-outbreak-but-there-are-none-available&hl=en&gl=us&strip=1&vwsrc=0> Accessed June 10, 2020.

S237. Khatiwada SA, Poudel KR. After the outbreak of novel coronavirus in China, consumption of masks in Nepali markets has significantly increased, leading to their acute shortage. Rising Nepal Daily. February 8, 2020. Available from: <https://webcache.googleusercontent.com/search?q=cache:fz4AvW5wF3sJ:https://risingnepaldaily.com/main-news/masks-can-do-only-so-much-to-protect-us+&cd=28&hl=en&ct=clnk&gl=us> Accessed June 10, 2020.

S238. No author listed. Decisions of the High-Level Coordination Committee for the Prevention and Control of COVID-19 [Nepal]. March 25, 2020. Available from:

<https://webcache.googleusercontent.com/search?q=cache:Gkx0jxclr6MJ:https://us.nepalembassy.gov.np/decisions-of-the-high-level-coordination-committee-for-the-prevention-and-control-of-covid-19-2/+&cd=17&hl=en&ct=clnk&gl=us> Accessed June 10, 2020.

S239. Sijapati A. Protecting Nepal’s elderly from COVID-19. Nepali Times. March 25, 2020. Available from: <https://www.nepalitimes.com/banner/protecting-nepals-elderly-from-covid-19/> Accessed June 10, 2020.

S240. Sharma V, Ortiz MR, Sharma N. Risk and Protective Factors for Adolescent and Young Adult Mental Health Within the Context of COVID-19: A Perspective From Nepal. J Adolesc Health. May 20, 2020. Available from: https://www.ncbi.nlm.nih.gov/pmc/articles/PMC7237905/ Accessed June 10, 2020.

S241. Singh DR, Sunuwar DR, Karki K, Ghimire S, Shrestha N. Knowledge and Perception Towards Universal Safety Precautions During Early Phase of the COVID-19 Outbreak in Nepal. Journal of Community Health. May 13, 2020. Available from: https://link.springer.com/content/pdf/10.1007/s10900-020-00839-3.pdf Accessed June 10, 2020.

S242. Alam K, Palaian S, Shankar PR, Jha N. General public’s knowledge and practices on face mask use during the COVID-19 pandemic: a cross-sectional exploratory survey from Dharan, Nepal. Available from: https://assets.researchsquare.com/files/rs-42148/v1/6800c8ad-e059-4fe9-a56a-3587646bb2fc.pdf Accessed August 1, 2020.

**Netherlands.**

S243. Pascoe R. Dutch start easing coronavirus restrictions, face masks a must on public transport. Dutch News NL. May 6, 2020. Available from: <https://www.dutchnews.nl/news/2020/05/dutch-start-easing-coronavirus-restrictions-face-masks-a-must-on-public-transport/> Accessed July 10, 2020.

**New Zealand**.

S244. No author listed. COVID-19: Use of masks in the community. Ministry of Health. August 6, 2020. Available from: <https://www.health.govt.nz/our-work/diseases-and-conditions/covid-19-novel-coronavirus/covid-19-health-advice-general-public/covid-19-use-masks-community> Accessed August 8, 2020.

**New Caledonia**.

S245. No author listed. New Caledonia: Most COVID-19 restrictions to be lifted May 4. Garda. April 30, 2020. Available from: <https://www.garda.com/crisis24/news-alerts/337661/new-caledonia-most-covid-19-restrictions-to-be-lifted-may-4-update-4> Accessed July 10, 2020.

**Nicaragua**.

S246. No author listed. Sancionada vicepresidenta Rosario Murillo anuncia distanciamiento social y uso de mascarillas, tras criminalizar su uso. Noticias. April 28, 2020. Available from: <https://100noticias.com.ni/nacionales/100581-sancionada-rosario-murillo-anuncia-uso-mascarillas/> Accessed August 9, 2020.

**Niger**.

S247. No author listed. Le couvre-feu est levé à Niamey. Niamey et les 2 Jours. May 13, 2020. Available from: <https://www.niameyetles2jours.com/la-gestion-publique/sante/1305-5443-le-couvre-feu-est-leve-a-niamey> Accessed July 11, 2020.

**Nigeria**.

S248. Adebowale N. Coronavirus: Nigeria’s health minister recommends use of improvised face masks. Premium Times. April 14, 2020. Available from:

<https://www.premiumtimesng.com/news/headlines/387761-coronavirus-nigerias-health-minister-recommends-use-of-improvised-face-masks.html> Accessed June 10, 2020.

S249. Ogoina D. COVID-19: The Need for Rational Use of Face Masks in Nigeria. American Society of Tropical Medicine and Hygiene. May 15, 2020. Available from: <https://www.ajtmh.org/content/journals/10.4269/ajtmh.20-0433> Accessed June 10, 2020.

S250. Isah MB, Abdulsalam M, Bello A, Ibrahim MI, Usman A, Nasir A, Abdulkadir B, Usman AR, Matazu KI, Sani A, Shuaibu A. Corona Virus Disease 2019 (COVID-19): Knowledge, attitudes, practices (KAP) and misconceptions in the general population of Katsina State, Nigeria. medRxiv. 2020.

**North Macedonia**.

S251. No author listed. North Macedonia: Face masks required in public spaces April 23. Garda World. April 23, 2020. Available from: <https://www.garda.com/crisis24/news-alerts/335411/north-macedonia-face-masks-required-in-public-spaces-april-23-update-7> Accessed July 11, 2020.

**Norway**.

S252. No author listed. Norway Makes First Face Mask Recommendation Since Pandemic Began. VOA News. August 14, 2020. Available from: <https://www.voanews.com/covid-19-pandemic/norway-makes-first-face-mask-recommendation-pandemic-began> Accessed September 26, 2020.

**Oman**.

S253. No author listed. Supreme Committee bans all Eid celebrations, wearing masks mandatory. Oman Daily Observer. May 18, 2020. Available from: <https://www.omanobserver.om/supreme-committee-bans-all-eid-celebrations-wearing-masks-mandatory/> Accessed July 11, 2020.

**Pakistan**.

S254. No author listed. Pakistan makes face masks mandatory in public. CNN. May 31, 2020. Available from: <https://edition.cnn.com/world/live-news/coronavirus-pandemic-05-31-20-intl/h_1ea61f10e67d78e1644aa489f88ae7c4> Accessed July 11, 2020.

S255. Malik S. Knowledge of COVID-19 Symptoms and Prevention among Pakistani Adults: A Cross-sectional Descriptive Study. PsyArXiv Available from: https://psyarxiv.com/wakmz Accessed June 28, 2020.

S256. Mirza TM, Ali R, Musarrat Khan HM. The knowledge and perception of COVID-19 and its preventive measures, in public of Pakistan. Pak Armed Forces Med J 2020; 70 (2): 338-45.

**Palestine**.

S257. No author listed. Wearing "forced" masks and gloves in Palestine and penalties for violators. Sada News. May 5, 2020. Available from: <https://www.sadanews.ps/news/59116.html> Accessed July 11, 2020.

**Panama**.

S258. No author listed. Es ‘obligatorio’ usar mascarilla si va a salir de casa, según ministra consejera de Salud. TVN Noticias. April 7, 2020. Available from: <https://www.tvn-2.com/nacionales/Coronavirus-en-Panama-obligatorio-usar-mascarilla-salir-casa_0_5550944856.html> Accessed May 8, 2020.

**Papua New Guinea**.

S259. Kombra U. Department of Education [Papua New Guinea]. Office of the Secretary. Secretary’s Circular Instruction. No. 5 of 2020. April 24, 2020. Available from: <https://covid19.info.gov.pg/files/28042020/Secretary%20Circular%20Instruction%205%20of%202020.pdf.pdf> Accessed July 11, 2020.

**Paraguay**.

S260. Ministerio de Salud Publica y Bienestar Social. Tapabocas de tela para uso en locales cerrados. April 7, 2020. Available from: <https://www.mspbs.gov.py/portal/20722/tapabocas-de-tela-para-uso-en-locales-cerrados.html> Accessed June 17, 2020.

**Peru**.

S261. Aquino M. Mascarillas gratis: Perú decreta su uso obligatorio para enfrentar el coronavirus. Infobae April 3, 2020. Available from: <https://www.infobae.com/america/agencias/2020/04/03/mascarillas-gratis-peru-decreta-su-uso-obligatorio-para-enfrentar-el-coronavirus-3/> Accessed May 8, 2020.

**Philippines**.

S262. Lasco G. Why Face Masks Are Going Viral. Sapiens. February 7, 2020. Available from: <https://www.sapiens.org/culture/coronavirus-mask/> Accessed May 9, 2020.

**Poland**.

S263. Gawlowski J, Ptak A. Vending machines selling face masks appear on Warsaw streets. Reuters. April 10, 2020. Available from: <https://www.reuters.com/article/us-health-coronavirus-poland-vending/vending-machines-selling-face-masks-appear-on-warsaw-streets-idUSKCN21S1MJ> Accessed June 19, 2020.

S264. Matusiak Ł, Szepietowska M, Krajewski P, Białynicki-Birula R, Szepietowski JC. Inconveniences due to the use of face masks during the COVID-19 pandemic: a survey study of 876 young people. Dermatologic Therapy. May 14, 2020. Available from: <https://onlinelibrary.wiley.com/doi/pdf/10.1111/dth.13567?casa_token=XcjP5hoHNVQAAAAA:6W_DYS_Ks5ObyZffz2abviHXOoyahPPIMfW9C40ONQu0aVwr91Divct2fysDS48DYTldkdaDVgWxS7_x> Accessed June 19, 2020.

**Portugal**.

S265. Almeida H. Portugal May Require Use of Masks When It Eases Confinement. Bloomberg. April 27, 2020. Available from: <https://www.bloomberg.com/news/articles/2020-04-27/portugal-may-require-masks-in-public-when-it-eases-confinement> Accessed July 11, 2020.

**Qatar**.

S266. Qatar: Face masks required for several sectors and public spaces April 22. Garda World. April 23, 2020. Available from: <https://www.garda.com/crisis24/news-alerts/335421/qatar-face-masks-required-for-several-sectors-and-public-spaces-april-22-update-17> Accessed July 11, 2020.

**Réunion**.^S370^

**Romania**.

S267. No author listed. Masks compulsory in Romania when virus lockdown ends. MSN. April 22, 2020. Available from: <https://www.msn.com/en-xl/europe/top-stories/masks-compulsory-in-romania-when-virus-lockdown-ends/ar-BB132Efe> Accessed July 11, 2020.

**Russia**.

S268. No author listed. В России выявили первые два случая заражения коронавирусом. TASS. 31 January 2020. Available from: <https://tass.ru/obschestvo/7656549> Accessed June 26, 2020.

S269. YS234. Roache M. How Russia's Coronavirus Outbreak Became One of the World's Worst. Time. May 15, 2020. Available from: <https://time.com/5836890/russia-coronavirus/> Accessed July 11, 2020.

**Rwanda**.

S270. Tasamba J. COVID-19: Rwanda, DR Congo make mask wearing mandatory. Anadolu Agency. April 19, 2020. Available from: <https://www.aa.com.tr/en/africa/covid-19-rwanda-dr-congo-make-mask-wearing-mandatory/1810165> Accessed July 11, 2020.

**Saint Kitts & Nevis**.

S271. Nurse M. St. Kitts And Nevis Confirms Two COVID-19 Cases. Caricom Today. March 25, 2020. Available from: <https://today.caricom.org/2020/03/25/st-kitts-and-nevis-confirms-two-covid-19-cases/> Accessed August 2, 2020.

S272. Health Official Recommends Wearing Face Masks in Public to Slow COVID-19 Spread. The St. Kitts & Nevis Observer. April 3, 2020. Available from: <https://www.thestkittsnevisobserver.com/health-official-recommends-wearing-face-masks-in-public-to-slow-covid-19-spread/> Accessed July 11, 2020.

S273. Saint Christopher and Nevis Statutory Rules and Orders. No. 12 of 2020. Emergency Powers (COVID-19) (No. 4) Regulations. April 7, 2020. Available from: <https://www.covid19.gov.kn/wp-content/uploads/2020/04/SRO_12_of_2020.pdf> Accessed July 11, 2020.

**Saint Lucia**.

S274. No author listed. UK National Is Saint Lucia’s First Coronavirus Case. St. Lucia Times. March 13, 2020. Available from: <https://stluciatimes.com/uk-national-is-saint-lucias-first-coronavirus-case/> Accessed August 2, 2020.

S275. No author listed. All of St Lucia's COVID-19 patients have now recovered. Loop News. April 22, 2020. Available from: <https://www.loopslu.com/content/all-st-lucias-covid-19-patients-have-now-recovered> Accessed July 8, 2020.

S276. Belmar-George S. Press Statement by Chief Medical Officer, Dr. Sharon Belmar-George on the safe use of medical masks. Government of Saint Lucia. April 7, 2020. Available from: <https://www.facebook.com/watch/?v=263340921365573> Accessed July 8, 2020.

**Saint Vincent & Grenadines**.

See St. Vincent & Grenadines.

**San Marino**.

S277. No author listed. San Marino. Mascherine e guanti: quando devono essere indossati. Libertas. April 18, 2020. Available from: <http://www.libertas.sm/notizie/2020/04/18/san-marino-mascherine-e-guanti-quando-devono-essere-indossati.html> Accessed July 12, 2020.

**São Tomé & Príncipe**.^S372^

S278. Covid-19: São Tomé e Príncipe com quatro casos de infecção. Radio France Internationale (in Portuguese). April 6, 2020. Available from: <https://www.rfi.fr/pt/s%C3%A3o-tom%C3%A9-e-pr%C3%ADncipe/20200406-covid-19-s%C3%A3o-tom%C3%A9-e-pr%C3%ADncipe-com-quatro-casos-de-infec%C3%A7%C3%A3o> Accessed July 7, 2020.

**Saudi Arabia**.

S279. No author listed. Saudi Arabia announces first case of coronavirus. Arab News. March 3, 2020. Available from: <https://www.arabnews.com/node/1635781/saudi-arabia> Accessed June 20, 2020.

S280. Al-Khudair D.Saudi health ministry: Face masks alone do not protect from COVID-19. Arab News. April 28, 2020. Available from: <https://www.arabnews.com/node/1666456/saudi-arabia> Accessed July 12, 2020.

S281. Ahmad K. Saudi Arabia Introduces New Social Distancing Rules Harpers Bazaar. May 31, 2020. Available from: <https://www.harpersbazaararabia.com/culture/saudi-introduces-fine-for-not-wearing-a-face-mask-in-the-public-covid-19-coronavirus-rules> Accessed July 12, 2020.

S282. Alkhamees AA, Alrashed SA, Alzunaydi AA, Almohimeed AS, Aljohani MS. The psychological impact of COVID-19 pandemic on the general population of Saudi Arabia. Comprehensive Psychiatry. 2020; 102. Available from: https://doi.org/10.1016/j.comppsych.2020.152192 Accessed July 31, 2020.

S283. Begum F. Knowledge, Attitudes, and Practices towards COVID-19 among B. Sc. Nursing Students in Selected Nursing Institution in Saudi Arabia during COVID-19 Outbreak: An Online Survey. Saudi Journal of Nursing and Health Care. 2020;3(7):194-8.

**Senegal**.^S373^

**Serbia**.

S284. No author listed. Health and Travel Alert: April 29, 2020. U.S. Embassy in Serbia. April 29, 2020. Available from: <https://rs.usembassy.gov/health-and-travel-alert-april-29-2020/> Accessed July 12, 2020.

S285. Cvetković VM, Nikolić N, Radovanović Nenadić U, Öcal A, K Noji E, Zečević M. Preparedness and Preventive Behaviors for a Pandemic Disaster Caused by COVID-19 in Serbia. International Journal of Environmental Research and Public Health. 2020 Jun; 17(11):4124.

**Seychelles**.

S286. No author listed. Seychelles: People in Seychelles Asked to Wear Face Masks in Crowded Areas to Prevent COVID-19. Allafrica. June 9, 2020. Available from: <https://allafrica.com/stories/202006100130.html> Accessed July 12, 2020.

**Sierra Leone**.

S287. No author listed. Sierra Leone has confirmed its first case of coronavirus, president says. Reuters. March 31, 2020. Available from: <https://www.reuters.com/article/us-health-coronavirus-leone-idUSKBN21I1MY> Accessed June 6, 2020.

S288. No author listed. Sierra Leone announces three-day lockdown against coronavirus. Medical Xpress. April 1, 2020. Available from: <https://medicalxpress.com/news/2020-04-sierra-leone-three-day-lockdown-coronavirus.html> Accessed June 6, 2020.

S289. Grieco K, Yusuf Y, Meriggi N. Rapid country study: Sierra Leone. COVID-19 Series. May 2020. Available from: <https://maintainsprogramme.org/wp-content/uploads/29-May-FinalV-Maintains-Covid-Rapid-Country-Report-Sierra-Leone_Revised-6.pdf> Accessed June 28, 2020.

**Singapore**.

S290. Ministry of Health, Singapore. Updates on COVID-19 (Coronavirus Disease 2019) Local Situation. <https://www.moh.gov.sg/covid-19> Accessed April 4, 2020.

S291. Ministry of Health, Singapore. Official Update of COVID -19 Situation in Singapore. Available from: <https://experience.arcgis.com/experience/7e30edc490a5441a874f9efe67bd8b89> Accessed April 4, 2020.

S292. Sim D. Coronavirus: what’s behind Singapore’s U-turn on wearing masks? South China Morning Post. April 3, 2020. Available from: <https://www.scmp.com/week-asia/health-environment/article/3078399/coronavirus-whats-behind-singapores-u-turn-wearing> Accessed May 30, 2020.

**Sint Maarten**.

S293. No author listed. Lockdown op Sint Maarten met drie weken verlengd. Antilliaans Dagblad. April 19, 2020. Available from: <https://antilliaansdagblad.com/sint-maarten/21349-lockdown-op-sint-maarten-met-drie-weken-verlengd> Accessed July 8, 2020.

**Slovakia**.

S294. Štefúnová I. Slovensko pritvrdilo v boji s vírusom. Nosenie rúšok je povinné. Pravda. March 15, 2020. Available from: <https://spravy.pravda.sk/domace/clanok/545657-slovensko-pritvrdilo-v-boji-s-virusom-nosenie-rusok-je-povinne/> Accessed May 15, 2020.

S295. Public Health Authority, Slovakia. "Opatrenie Úradu verejného zdravotníctva Slovenskej republiky pri ohrození verejného zdravia" March 24, 2020. Available from:

<http://www.uvzsr.sk/docs/info/covid19/Opatrenie_UVZSR_povinnost_nosit_ruska_24032020.pdf> Accessed May 15, 2020.

**Slovenia**.

S296. STA. COVID-19 & Slovenia, Night 29 March: Movement Restrictions, Mandatory Masks, More Aid for Individuals. Total Slovenia News. March 29, 2020. Available from: <https://www.total-slovenia-news.com/politics/5951-covid-19-slovenia> Accessed May 8, 2020.

**Somalia**.

S297. Ahmed MA, Siewe Fodjo JN, Gele AA, Farah AA, Osman S, Guled IA, Ali AM, Colebunders R. COVID-19 in Somalia: Adherence to Preventive Measures and Evolution of the Disease Burden. Pathogens. 2020 Sep;9(9):735.

**South Africa**.

S298. Human Sciences Research Council. HSRC Responds to the COVID-19 Outbreak. Available from: <http://www.hsrc.ac.za/uploads/pageContent/11529/COVID-19%20MASTER%20SLIDES%2026%20APRIL%202020%20FOR%20MEDIA%20BRIEFING%20FINAL.pdf> Accessed July 1, 2020.

S299. Dr Zweli Mkhize recommends the widespread use of cloth masks. Republic of South Africa Health Department. April 10, 2020. Available from: <https://sacoronavirus.co.za/2020/04/10/dr-zweli-mkhize-recommends-the-widespread-use-of-cloth-masks/> Accessed May 8, 2020.

S300. No author listed. South Africa: Mandatory wearing of masks under partial easing of restrictions from May 1. April 26, 2020. Available from: <https://www.garda.com/crisis24/news-alerts/336291/south-africa-mandatory-wearing-of-masks-under-partial-easing-of-restrictions-from-may-1-update-12> Accessed July 6, 2020.

**South Korea**.

S301. No author listed. With fears of Coronavirus spreading, do face masks really work? Associated Press. Available from: <https://fox8.com/news/health/with-fears-of-coronavirus-spreading-do-face-masks-really-work/> Accessed June 6, 2020.

S302. Taylor K. Costco is selling out of surgical masks in South Korea, as the country battles the spread of the coronavirus. Business Insider. February 3, 2020. Available from: <https://www.businessinsider.com/costco-is-selling-out-of-surgical-masks-in-south-korea-2020-2> Accessed June 6, 2020.

S303. Kim ET. How South Korea Solved Its Face Mask Shortage: Neighborhood pharmacists and government intervention were the secret weapons. New York Times. April 1, 2020. Available from: <https://www.nytimes.com/2020/04/01/opinion/covid-face-maskshortage.html> Accessed April 17, 2020.

**South Sudan**.

S304. Ajack M. South Sudan 51st of 54 African nations to report virus case. AP News. April 5, 2020 Available from: <https://apnews.com/5307b01c4db913048387a3702aefbbe2> Accessed August 2, 2020.

S305. No author listed. South Sudan: COVID-19 Update, 20 April – 03 May 2020. Reliefweb. May 3, 2020. Available from: <https://reliefweb.int/report/south-sudan/south-sudan-covid-19-update-20-april-03-may-2020> Accessed July 12, 2020.

**Spain**.

S306. No author listed. First confirmed coronavirus case in Spain in La Gomera, Canary Islands. Outbreak News Today. February 3, 2020. Available from: <http://outbreaknewstoday.com/first-confirmed-coronavirus-case-in-spain-in-la-gomera-canary-islands-20628/> Accessed June 20, 2020.

S307. Alcuten J. Valencia confirma el primer muerto con coronavirus en España: un hombre de 69 años que falleció el 13 de febrero. 20 Minutos. March 3, 2020. Available from: <https://www.20minutos.es/noticia/4174137/0/primer-muerto-coronavirus-espana/> Accessed June 20, 2020.

S308. Sawer P. Spain to hand out free face masks for commuters to help return to work. The Telegraph. April 11, 2020. Available from: <https://www.telegraph.co.uk/news/2020/04/11/spain-hand-face-masks-allow-limited-return-work/> Accessed May 8, 2020.

**Sri Lanka**.

S309. No author listed. Sri Lanka adopts prevention measures after first coronavirus case. Outlook. January 29, 2020. Available from: <https://www.outlookindia.com/newsscroll/sri-lanka-adopts-prevention-measures-after-first-coronavirus-case/1720276> Accessed June 7, 2020.

S310. Sri Lanka makes wearing face masks mandatory when stepping out. Colombo Page. April 11, 2020. Available from: <http://www.colombopage.com/archive_20A/Apr11_1586618131CH.php> Accessed May 8, 2020.

**St. Vincent & Grenadines**.

S311. Keizer-Beache S. HEOC/COVID-19 Task Force Advisory: social (physical) distancing –spiritual and social wellbeing – Easter weekend. Ministry of Health, Wellness, and the Environment. Saint Vincent and the Grenadines. April 5, 2020. Available from: <http://health.gov.vc/health/images/PDF/stories/HEOC_COVID-19-advisory---Easter-2020---1.pdf> Accessed July 12, 2020.

S312. Keizer-Beache S. Health Services Subcommittee Advisory: Safe Use of Face Coverings – Masks. Ministry of National Security. St. Vincent and the Grenadines. April 26, 2020. Available from: <http://health.gov.vc/health/images/PDF/News_Release_HEALTH_SERVICES_COMMITTEE_MASK_ADVISORY_NEOC_7_FINAL.pdf> Accessed July 12, 2020.

**Sudan**.

S313. No author listed. Face mask and fruit prices soar in Sudan. Dabanga. March 16, 2020. Available from: <https://www.dabangasudan.org/en/all-news/article/face-mask-and-fruit-prices-soar-in-sudan>

S314. No author listed. Sudan Flash Update, 16 Mar 2020: Sudan creates two COVID-19 isolation centres in Khartoum State. Available from: <https://reliefweb.int/report/sudan/sudan-flash-update-16-mar-2020-sudan-creates-two-covid-19-isolation-centres-khartoum> Accessed May 26, 2020.

S315. Mousa KN, Saad MM, Abdelghafor MT. Knowledge, attitudes, and practices surrounding COVID-19 among Sudan citizens during the pandemic: an online cross-sectional study. Sudan Journal of Medical Sciences (SJMS). 2020; 15:32-45.

**Suriname**.

S316. No author listed. Breaking: Al 23 Covid-besmettingen in Suriname (update). Star Nieuws. May 31, 2020. Available from: <https://www.starnieuws.com/index.php/welcome/index/nieuwsitem/58911> Accessed July 12, 2020.

**Switzerland**.

S317. No author listed. Swiss parliament to debate compulsory mask requirement on Friday. The Local. June 17, 2020. Available from: <https://www.thelocal.ch/20200610/will-switzerland-introduce-a-mask-requirement> Accessed July 12, 2020.

**Syria**.

S318. Alhamid A, Aljarad Z, Alhamid A. Knowledge and behaviors towards COVID-19 among University of Aleppo students: an online cross-sectional survey. Available from: <https://www.medrxiv.org/content/10.1101/2020.07.11.20151035v1> Accessed July 31, 2020.

**Taiwan**.

S319. Blanchard B. Taiwan ups Chinese visitor curbs, to stop mask exports. Reuters. January 27, 2020. Available from: <https://www.reuters.com/article/us-china-health-taiwan/taiwan-ups-chinese-visitor-curbs-to-stop-mask-exports-idUSKBN1ZQ1C6> Accessed May 9, 2020.

S320. Chiu WT, Laporte RP, Wu J. Determinants of Taiwan’s Early Containment of COVID-19 Incidence. AJPH. 2020; 110(7): 943-4.

S321. Chiang CH, Chiang CH, Chiang CH. Maintaining mask stockpiles in the COVID-19 pandemic: Taiwan as a learning model. Infection Control & Hospital Epidemiology 2020; 1-2.

**Thailand**.

S322. No author listed. Bangkok residents grapple with shortage of N95 masks as smog continues to engulf city. Straits Times. January 16, 2019. Available from: <https://www.straitstimes.com/asia/se-asia/bangkok-residents-grapple-with-shortage-of-n95-masks-as-smog-continues-to-engulf-city> Accessed September 30, 2020.

S323. Thongthab S. Ministry of Public Health Enhances Disease Prevention and Control. The Bangkok Insight. January 31, 2020. Available from: <https://www.thebangkokinsight.com/282702/> Accessed May 9, 2020.

S324. Bureau of Information Office of the Permanent Secretary of MOPH [of Thailand]. Novel Coronavirus 2019, January 28, 2020 News Report. Available from: <https://pr.moph.go.th/?url=pr/detail/2/04/137810/> Accessed June 6, 2020.

S325. Bureau of Information Office of the Permanent Secretary of MOPH [of Thailand]. Novel Coronavirus (2019-nCoV) Report, January 31, 2020. Available from: <https://pr.moph.go.th/?url=pr/detail/2/04/137947/> Accessed June 6, 2020.

S326. Bureau of Information Office of the Permanent Secretary of MOPH [of Thailand]. Novel Coronavirus (2019-nCoV) News Report, January 30, 2020. Available from: <https://pr.moph.go.th/?url=pr/detail/2/04/137895/> Accessed June 6, 2020.

S327. Doung-ngern P, Suphanchaimat R, Panjagampatthana A, Janekrongtham C, Ruampoom D, Daochaeng N, Eungkanit N, Pisitpayat N, Srisong N, Yasopa O, Plernprom P. Associations between wearing masks, washing hands, and social distancing practices, and risk of COVID-19 infection in public: a cohort-based case-control study in Thailand. medRxiv. Accessed June 19, 2020.

**Timor-Leste**.

S328. No author listed. East Timor confirms first case of coronavirus: health ministry. Reuters. March 21, 2020. Available from: <https://www.reuters.com/article/us-health-coronavirus-timor/east-timor-confirms-first-case-of-coronavirus-health-ministry-idUSKBN2180BI> Accessed August 2, 2020.

S329. No author listed. Health Alert – U.S. Embassy Dili, Timor-Leste (April 7, 2020). April 7, 2020. Available from: <https://tl.usembassy.gov/health-alert-u-s-embassy-dili-timor-leste-april-7-2020/> Accessed July 12, 2020.

**Togo**.

S330. No author listed. Coronavirus: Le Togo avance progressivement vers l'option du "Masque pour tous". Togo First. April 20, 2020. Available from: <https://www.togofirst.com/fr/sante/2004-5370-coronavirus-le-togo-avance-progressivement-vers-loption-du-masque-pour-tous> Accessed July 12, 2020.

**Trinidad & Tobago**.

S331. No author listed. Wear Masks. Trinidad Express Newspaper. April 5, 2020. Available from: <https://trinidadexpress.com/newsextra/wear-masks/article_4167a0d4-7765-11ea-99b6-8b1c79a31427.html> Accessed May 9, 2020.

S332. Naraynsingh V, Harnanan D, Maharaj R, Naraynsingh R. COVID-19 in the West Indies: Trinidad and Tobago Experience. Journal of Lumbini Medical College. 2020; 8(1): 1-2. Available from: https://www.jlmc.edu.np/index.php/JLMC/article/view/347 Accessed June 28, 2020.

**Tunisia**.

S333. No author listed. La Tunisie décide l’obligation du port du masque pour toute la population. GNet News. April 7, 2020. Available from: <https://news.gnet.tn/la-tunisie-decide-lobligation-du-port-du-masque-pour-toute-la-population/> Accessed May 30, 2020.

**Turkey**.

S334. No author listed. Coronavirus: Turkey imposes curfew on youth, shuts borders of 31 cities. Middle East Eye. April 3, 2020. Available from: <https://www.middleeasteye.net/news/coronavirus-turkey-erdogan-youth-under-curfew-restrictions-curb-pandemic> Accessed May 9, 2020.

**Turks and Caicos**.

S335. Cartwright-Robinson S. Premier’s National COVID-19 Address. Turks and Caicos. April 30, 2020. Available from: <https://www.gov.tc/moh/coronavirus/news/147-premier-s-national-covid-19-address-30-april-2020> Accessed July 14, 2020.

**Uganda**.

S336. No author listed. Museveni: Uganda’s lockdown will ease, but facemasks will be required by all. CGTN Africa. May 1, 2020. Available from: <https://webcache.googleusercontent.com/search?q=cache:pmS86NKyGyQJ:https://africa.cgtn.com/2020/05/01/museveni-ugandas-lockdown-will-ease-but-facemask-mandate-will-remain/+&cd=20&hl=en&ct=clnk&gl=us> Accessed July 14, 2020.

**Ukraine**.

S337. No author listed. Ukraine tightens restrictions to fight coronavirus spread. Reuters. April 3, 2020. Available from: <https://www.reuters.com/article/us-health-coronavirus-ukraine-measures/ukraine-tightens-restrictions-to-fight-coronavirus-spread-idUSKBN21L24L> Accessed June 24, 2020.

S338. No author listed. За шашлики у парку та відсутність маски на вулиці поки не штрафуватимуть, - МВС. Zik. March 30, 2020. Available from: <https://zik.ua/news/ludyna/za_shashlyky_u_parku_ta_vidsutnist_masky_na_vulytsi_poky_ne_shtrafuvatymut_mvs_963838> Accessed July 25, 2020.

**United Arab Emirates**.

S339. Nandkeolyar KH. Coronavirus in UAE: Four of a family infected. Gulf News. January 29, 2020. Available from: <https://gulfnews.com/uae/health/coronavirus-in-uae-four-of-a-family-infected-1.1580273983681> Accessed June 20, 2020.

S340. Venkataraman V. Coronavirus: UAE Ministry of Health Warns Against Usage Of Masks. Curly Tales. February 29, 2020. Available from: <https://curlytales.com/coronavirus-uae-ministry-of-health-warns-against-usage-of-masks/> Accessed June 20, 2020.

S341. No author listed. Health Alert: U.S. Embassy Abu Dhabi and U.S. Consulate General Dubai (March 28, 2020). Available from: <https://ae.usembassy.gov/health-alert-u-s-embassy-abu-dhabi-and-u-s-consulate-general-dubai-march-28-2020/> Accessed June 20, 2020.

**United Kingdom**.

S342. Ball T. Hunt for contacts of coronavirus-stricken pair in York. The Sunday Times. January 31, 2020. Available from: <https://www.thetimes.co.uk/article/hunt-for-contacts-of-coronavirus-stricken-pair-in-york-dh363qf8k> Accessed June 20, 2020.

S343. No author listed. Coronavirus: Cover faces in some public areas, people in England told. BBC. May 11, 2020. Available from: <https://www.bbc.com/news/uk-52620556> Accessed July 14, 2020.

S344. No author listed. Face coverings to be made compulsory on public transport in England. June 4, 2020. Available from: <https://www.theguardian.com/world/2020/jun/04/face-masks-to-be-made-compulsory-on-public-transport-in-england> Accessed July 14, 2020.

**United States**.

S345. Ritter Z, Brenan M. New April Guidelines Boost Perceived Efficacy of Face Masks. Gallup. May 13, 2020. Available from: <https://news.gallup.com/poll/310400/new-april-guidelines-boost-perceived-efficacy-face-masks.aspx> Accessed June 1, 2020.

S346. Tri-County Health Department. Mask-wearing Observational Data. Percentage of people observed wearing a mask. Adams, Arapahoe, and Douglas Counties. Available from: <https://public.tableau.com/profile/adam.anderson#!/vizhome/TCHD_COVID19_TempCaseDashboard_06162020/Dashboard1> Accessed August 11, 2020.

S347. Holshue ML, DeBolt C, Lindquist S, Lofy KH, Wiesman J, Bruce H, Spitters C, Ericson K, Wilkerson S, Tural A, Diaz G. First case of 2019 novel coronavirus in the United States. New England Journal of Medicine. 2020; 382: 929-936.

S348. United States Centers for Disease Control. Recommendation Regarding the Use of Cloth Face Coverings, Especially in Areas of Significant Community-Based Transmission. Available from: <https://www.cdc.gov/coronavirus/2019-ncov/preventgetting-sick/cloth-face-cover.html> Accessed April 3, 2020.

S349. Beckage B, Buckley T, Beckage ME. Prevalence of mask wearing in northern Vermont in response to SARS-CoV-2. medRxiv. 2020.

S350. Haischer MH, Beilfuss R, Hart MR, Opielinski L, Wrucke D, Zirgaitis G, Uhrich TD, Hunter SK. Who is wearing a mask? Gender-, age-, and location-related differences during the COVID-19 pandemic. medRxiv. 2020.

**Uruguay**.

S351. No author listed. Informe de situación en relación al coronavirus COVID-19 en Uruguay del 10/4/20. Ministerio de Salud Publica. April 10, 2020. Available from: <https://www.gub.uy/sistema-nacional-emergencias/comunicacion/comunicados/informe-situacion-relacion-coronavirus-covid-19-uruguay-del-10420> Accessed July 14, 2020.

**Uzbekistan**.

S352. No author listed. Uzbekistan confirms first coronavirus case – govt. Reuters. March 15, 2020. Available from: <https://www.reuters.com/article/health-coronavirus-uzbekistan/uzbekistan-confirms-first-coronavirus-case-govt-idUSL8N2B802F> Accessed June 10, 2020.

S353. COVID-19 Information. US Embassy in Uzbekistan. May 1, 2020. Available from: <https://uz.usembassy.gov/covid-19-information/> Accessed May 9, 2020.

**Venezuela**.

S354. AFP. Coronavirus: Maduro tells Venezuelans to make their own masks. March 17, 2020. Available from: <https://www.youtube.com/watch?v=EjTsjJNCAuc> Accessed May 15, 2020.

S355. Berwick A, Nava M, Kinosian S. Venezuela confirms coronavirus cases amid public health concerns. Reuters. March 14, 2020. Available from: <https://www.physiciansweekly.com/venezuela-confirms-coronavirus-cases/> Accessed May 15, 2020.

S356. Valderrama S. Venezuelans sew homemade face masks amid coronavirus quarantine. Reuters. March 20, 2020. Available from: <https://www.reuters.com/article/us-health-coronavirus-venezuela-masks/venezuelans-sew-homemade-face-masks-amid-coronavirus-quarantine-idUSKBN2171W2> Accessed May 9, 2020.

S357. No author listed. Venezuela confirms first coronavirus death: official. Reuters. March 26, 2020. Available from: <https://www.reuters.com/article/us-health-coronavirus-venezuela-death-idUSKBN21D3Q3> Accessed July 15, 2020.

S358. Charles J, Tavel J, Wyss J, Gamez Torres N. Coronavirus update: Some of the measures taken by Latin American, Caribbean nations. Miami Herald. March 24, 2020. Available from: <https://web.archive.org/web/20200325195723/https://www.miamiherald.com/news/nation-world/world/americas/haiti/article241249651.html> Accessed July 7, 2020.

**Vietnam**.

S359. Taylor K. I've been traveling in Asia for 3 weeks amid the deadly coronavirus outbreak, and actually catching the virus is far from my biggest fear. Business Insider. February 18, 2020. Available from: <https://www.businessinsider.com/travel-in-asia-during-coronavirus-outbreak-2020-2#so-for-now-my-travels-continue-17> Accessed May 9, 2020.

S360. Minh A. Coronavirus fears spur sales of face masks. VN Express. January 27, 2020. Available from: <https://e.vnexpress.net/news/business/economy/coronavirus-fears-spur-sales-of-face-masks-4047111.html> Accessed January 6, 2020.

S361. Vu K, Nguyen P, Pearson J. After aggressive mass testing, Vietnam says it contains coronavirus outbreak. Reuters. April 29, 2020. Available from: <https://www.reuters.com/article/us-health-coronavirus-vietnam-fight-insi/after-aggressive-mass-testing-vietnam-says-it-contains-coronavirus-outbreak-idUSKBN22B34H> Accessed June 13, 2020.

S362. No author listed. All in Vietnam must wear face mask in public places for coronavirus prevention. The Star. March 16, 2020. Available from: <https://www.thestar.com.my/news/regional/2020/03/16/all-in-vietnam-must-wear-face-mask-in-public-places-for-coronavirus-prevention> Accessed September 24, 2020.

S363. Nguyen NP, Hoang TD, Tran VT, Vu CT, Siewe Fodjo JN, Colebunders R, Dunne MP, Vo VT. Preventive behavior of Vietnamese people in response to the COVID-19 pandemic. medRxiv. 2020. Available from: https://www.medrxiv.org/content/10.1101/2020.05.14.20102418v1 Accessed June 15, 2020.

S364. Pham TQ, Rabaa M, Duong LH, Dang TQ, Dai Tran Q, Quach HL, Hoang NA, Phung DC, Ngu ND, Tran AT, La NQ. The first 100 days of SARS-CoV-2 control in Vietnam. medRxiv. 2020.

**Yemen**.

S365. Kadi HO. Yemen is free of COVID-19. International Journal of Clinical Virology. 2020: 32-3. Available from: <http://www.yemenuniversity.com/ar/content/uploads/2020/05/ijcv-aid1012.pdf> Accessed June 30, 2020.

**Zambia**.

S366. Chisenga O, Mbulo E. Wear masks to mitigate COVID-19-Chilufya. The Mast. April 5, 2020. Available from: <https://www.themastonline.com/2020/04/05/wear-masks-to-mitigate-covid-19-chilufya/> Accessed June 18, 2020.

S367. Center for International Health, Education, and Biosecurity. University of Maryland. COVID-19 Clinical Guidance and Mask Making in Zambia. Available from: <http://ciheb.org/NEWS/COVID-19-Clinical-Guidance-and-Mask-Making-in-Zambia/> Accessed June 18, 2020.

S368. U.S. Embassy Lusaka Zambia. Health Alert: Zambia, Wearing Of Masks In Public Now Mandatory. April 16, 2020. Available from: <https://www.osac.gov/Country/Zambia/Content/Detail/Report/251986cc-38ee-4e4c-ad78-1875ef77459e> Accessed June 18, 2020.

**Zimbabwe**.

S369. Dirani TH. Zimbabwe Will Now Arrest Anyone Not Wearing Face Mask In Public During Lockdown. iHarare. May 3, 2020. Available from: <https://iharare.com/people-not-wearing-masks-face-arrest/> Accessed July 14, 2020.

**Multiple Regions**.

S370. No author listed. Réouverture des plages, quatorzaine Outre-mer, port du masque: Édouard Philippe détaille le plan de deconfinement. Franceinfo. May 7, 2020. Available from: <https://la1ere.francetvinfo.fr/coronavirus-edouard-philippe-detaille-le-plan-de-deconfinement-a-quatre-jours-du-11-mai-830390.html> Accessed July 11, 2020.

S371. Perrotta D, Grow A, Rampazzo F, Cimentada J, Del Fava E, Gil-Clavel S, Zagheni E. Behaviors and attitudes in response to the COVID-19 pandemic: Insights from a cross-national Facebook survey. medRxiv. 2020.

S372. U.S. Embassy in Gabon. COVID-19 Information for Gabon and São Tomé and Príncipe. April 23, 2020. Available from: https://web.archive.org/web/20200426105023/https://ga.usembassy.gov/u-s-citizen-services/coronavirus-update/ Accessed May 30, 2020.

S373. Herman T, Maarek E, Wilde N, Adao F, Abousaada S. COVID-19: Initial responses of certain African countries. Africa Notes. April 30, 2020. Available from: <https://www.lexology.com/library/detail.aspx?g=9ddb5508-c7eb-4519-b2d5-1eeae40268c8> Accessed July 7, 2020.
